# Steering perovskite precursor solutions for multijunction photovoltaics

**DOI:** 10.1038/s41586-024-08546-y

**Published:** 2024-12-23

**Authors:** Shuaifeng Hu, Junke Wang, Pei Zhao, Jorge Pascual, Jianan Wang, Florine Rombach, Akash Dasgupta, Wentao Liu, Minh Anh Truong, He Zhu, Manuel Kober-Czerny, James N. Drysdale, Joel A. Smith, Zhongcheng Yuan, Guus J. W. Aalbers, Nick R. M. Schipper, Jin Yao, Kyohei Nakano, Silver-Hamill Turren-Cruz, André Dallmann, M. Greyson Christoforo, James M. Ball, David P. McMeekin, Karl-Augustin Zaininger, Zonghao Liu, Nakita K. Noel, Keisuke Tajima, Wei Chen, Masahiro Ehara, René A. J. Janssen, Atsushi Wakamiya, Henry J. Snaith

**Affiliations:** 1 Clarendon Laboratory, Department of Physics, University of Oxford, Oxford, UK; 2 Institute for Chemical Research, Kyoto University, Gokasho Uji-city, Kyoto, Japan; 3 Research Center for Computational Science, Institute for Molecular Science, Okazaki, Japan; 4 Polymat, University of the Basque Country – UPV/EHU, Donostia-San Sebastian, Spain; 5 Wuhan National Laboratory for Optoelectronics, Huazhong University of Science and Technology (HUST), Wuhan, China; 6 Hubei Optics Valley Laboratory, Wuhan, China; 7 Molecular Materials and Nanosystems, Eindhoven University of Technology, Eindhoven, The Netherlands; 8 Institute for Complex Molecular Systems, Eindhoven University of Technology, Eindhoven, The Netherlands; 9 National Thin-Film Cluster Facility for Advanced Functional Materials, Department of Physics, University of Oxford, Oxford, UK; 10 RIKEN Center for Emergent Matter Science (CEMS), Wako, Saitama, Japan; 11 Instituto Universitario de Ciencia de los Materiales (ICMUV), Universitat de València, Paterna, Spain; 12 Institut für Chemie, Humboldt-Universität zu Berlin, Berlin, Germany; 13 Dutch Institute for Fundamental Energy Research, Eindhoven, The Netherlands

**Keywords:** Devices for energy harvesting, Chemical physics, Solar cells, Solar cells

## Abstract

Multijunction photovoltaics (PVs) are gaining prominence owing to their superior capability of achieving power conversion efficiencies (PCEs) beyond the radiative limit of single-junction cells^1–8^, for which improving narrow-bandgap (NBG) tin–lead perovskites is critical for thin-film devices^9^. Here, with a focus on understanding the chemistry of tin–lead perovskite precursor solutions, we find that Sn(ii) species dominate interactions with precursors and additives and uncover the exclusive role of carboxylic acid in regulating solution colloidal properties and film crystallization and ammonium in improving film optoelectronic properties. Materials that combine these two functional groups, amino acid salts, considerably improve the semiconducting quality and homogeneity of perovskite films, surpassing the effect of the individual functional groups when introduced as part of separate molecules. Our enhanced tin–lead perovskite layer allows us to fabricate solar cells with PCEs of 23.9%, 29.7% (certified 29.26%) and 28.7% for single-junction, double-junction and triple-junction devices, respectively. Our 1-cm^2^ triple-junction devices show PCEs of 28.4% (certified 27.28%). Encapsulated triple-junction cells maintain 80% of their initial efficiencies after 860 h maximum power point tracking (MPPT) in ambient. We further fabricate quadruple-junction devices and obtain PCEs of 27.9% with the highest open-circuit voltage of 4.94 V. This work establishes a new benchmark for multijunction PVs.

## Abstract (web summary)

Understanding the chemistry of perovskite precursor solutions enables improved film optoelectronic properties, allowing the fabrication of multijunction solar cells achieving power conversion efficiencies beyond the radiative limit of single-junction cells.

## Main

Integrating several absorber bandgaps into tandem PVs allows for more effective solar energy conversion, thanks to minimized carrier thermalization losses^4^. With tremendous efforts invested in the field, the leading efficiency of monolithic ‘two-terminal’ perovskite-on-silicon tandem cells is 34.6% (ref. ^10^), with full-scale industrial modules certified at 26.9% (ref. ^11^). Parallel to this, tandem PV technology has also provided high efficiencies of 24.2% (ref. ^12^), 24.9% (ref. ^13^), 25.2% (ref. ^14^) and 30.1% (ref. ^15^) (highest literature report 28.5% (ref. ^16^)) for perovskite-on-Cu(InGa)Se_2_, perovskite-on-CuInSe_2_, organic-on-perovskite and ‘all-perovskite’ counterpart devices, respectively. Among these material combinations, all-perovskite tandems stand out for their superior ability to integrate multijunction cells monolithically^17–21^, thanks to the exceptional bandgap tunability of perovskite materials^22,23^. On the basis of the principle of detailed balance^8^, the double-junction, triple-junction and quadruple-junction cells show maximum theoretical efficiencies of about 45%, 52% and 56%, respectively, under 1-sun irradiance^24,25^. A further increase in the number of integrated junctions beyond four, may, however, increase the levelized cost of electricity^26^, as the added fabrication costs may not justify the slight improvement in efficiency, resulting in a longer payback time. In terms of maximizing energy yield under real-world operation, the optimum number of junctions is around five^24^. Targeting four or five junctions should ultimately lead to the highest energy-yielding PVs.

For all-perovskite double-junction and multijunction cells, further advancement is largely hindered by the challenging but necessary tin–lead perovskite material (*E*_g_ ≈ 1.25 eV), owing to the facile oxidation of Sn(ii) to Sn(iv) and the difficult-to-control crystallization dynamics^27^. The field has extensively investigated suppressing Sn(ii) oxidation using various additives^28–36^ or non-oxidizing solvents^37^ and improving the performance through surface modifications^9^. By contrast, studies on precursor solution chemistry and their impacts on the crystallization process and film properties are limited. We have previously found that an amino acid salt glycine hydrochloride could preferentially accumulate at the bottom interface, improving device hole extraction^38^. This strategy has been widely used for fabricating efficient single-junction and tandem cells^9^, whereas understanding the core mechanism and how to further adapt this approach to make further gains is underexplored. To understand the role of amino acid salts further and decouple the effect of functional groups from a solution chemistry perspective, herein we select three structurally similar molecules that individually contain ammonium, carboxyl or both motifs on a single molecule, that is, phenethylammonium chloride (PEA), 3-phenylpropionic acid (PPA) and l-phenylalanine hydrochloride (PhA), respectively.

## Solution chemistry

**Fig. 1:**
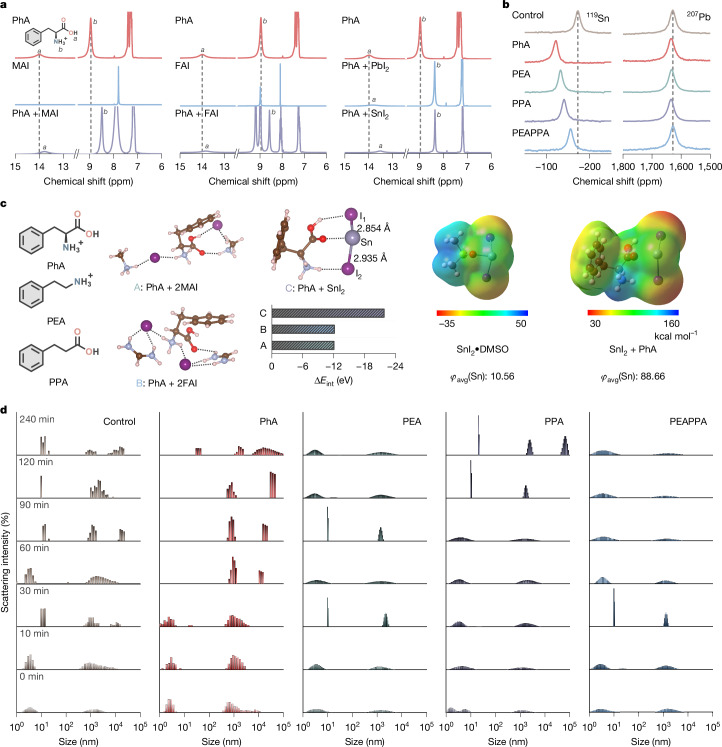
Chemistry of Sn–Pb perovskite precursor solutions. **a**, ^1^H NMR spectra of PhA in combination with different perovskite precursors dissolved in a solvent mixture of DMSO-*d*_6_ and DMF-*d*_7_ (1:3, v:v). **b**, ^119^Sn NMR and ^207^Pb NMR spectra showing Sn(ii) and Pb(ii) signals in perovskite precursor solutions containing different additives. **c**, Left, chemical structure of PhA, PEA and PPA. Middle, DFT-calculation-generated potential chemical interaction configurations for PhA with MAI, FAI and SnI_2_ species, along with the bar plot of calculated interaction energies (Δ*E*_int_) for A, B and C species. Right, Gaussian-calculated electrostatic potentials (*φ*) are shown for SnI_2_•DMSO adduct and SnI_2_ + PhA. The colour bar from red to blue marks the increase of electropositivity. **d**, DLS results for perovskite precursor solutions measured at different points after preparation.

We use solution nuclear magnetic resonance (NMR) to study perovskite precursor solutions containing PhA, PEA and PPA (Fig. 1 and Supplementary Figs. 1–5). First, we combine PhA with different perovskite precursor components individually. The combination with A-site organic components, that is, MAI and FAI, leads to upfield shifts for the protons in the ammonium and carboxyl groups of PhA (Fig. 1a). The shielding of these protons originates from a slightly higher electronic density around them, implying the presence of H-bonding of PhA, which could be with both perovskite ammonium components and iodide ions. The lower shift found when in the presence of CsI indicates that PhA—and amino acid salts in general—establish favourable interactions with ammonium-based halide precursors (Supplementary Fig. 3). This is probably because of the addition of H-bonding formed between –C=O of PhA and protons from the ammonium cations compared with the inorganic Cs^+^, in which only ionic interactions are present. When PhA is added, the signal at 8.99 ppm, integrating for the four FA ammonium protons, splits into two peaks at 8.98 and 9.19 ppm. These signals correspond to the *cis* and *trans* protons, with respect to the C-bound proton, observable owing to the diamagnetic anisotropy present in conjugated systems. We interpret this as a strengthened conjugation and/or interrupted rapid proton transfer between two –NH_2_ groups of FA^+^ owing to the establishment of PhA–FAI interactions. We also observe the same behaviour after the addition of PhA to the FAPbI_3_ solution (Supplementary Fig. 4). In the case of the FASnI_3_ solution, however, this splitting is already present before introducing the additives, indicating a stronger interaction between components in Sn-based perovskite precursors in comparison with FAPbI_3_ solutions. Mixed Sn–Pb perovskite precursor solutions show the two signals of FA ammonium protons in ^1^H NMR (Supplementary Fig. 5). We then investigate the interactions of PhA with the metal-based precursors. In the presence of metal halides PbI_2_ and SnI_2_, the upfield shift of PhA ammonium protons further increased. The carboxyl signal also shows a variation, notably higher for the case of SnI_2_. These results indicate that Sn-related chemistry dominates precursor pre-organization in this mixed-metal solution.

We carry out ^119^Sn and ^207^Pb NMR experiments, in which we observe invariable Pb^2+^ signals but considerable downfield shifts for Sn^2+^ signals when in the presence of ammonium and/or carboxyl (Fig. 1b). This effect could be attributed to the inert-pair effect of Pb^2+^ with the outermost atomic 6s orbital remaining unshared and the steric activity (more accessible) of the SnX_2_ (X: halides) compounds according to the valence-shell electron-pair repulsion theory. When adding ammonium and carboxylic acid together, denoted as PEAPPA, to the system, we observe a reduced chemical shift for the Sn(ii) signal compared with the PEA and PPA cases. By contrast, the effects from both functional groups are not masked but summed when combined in a sterically confined structure as an amino acid salt (PhA), suggesting separate and synergistically strengthened roles of each functional group. A chemical downfield shift for Sn^2+^ species indicates a reduced electron density on these nuclei, suggesting that strong electron-donating species in solution, for example, dimethyl sulfoxide (DMSO), are complexing the metal cation to a lesser degree. We thus speculate that the pre-forming of the H-bonding interaction generated between PEA and PPA weakens the interactions of each other towards the Sn-based species. However, presenting both groups at the same site with a confined geometry will suppress the H-bonding interaction and reduce the entropic cost, enabling strong interactions. Relativistic effects from the combination of Sn/Pb and heavy halogen atoms contribute to chemical shifts and are influenced by several factors, including solvent type and concentration. In this study, we keep these external parameters as constant as possible between the samples, so that we can interpret the chemical shifts to result from changes in the coordination environment of tin cations, as corroborated by the clear shifts in ^1^H NMR. Overall, these results indicate the ability of both functional groups to interact with perovskite precursors in solution, and amino acid structures offer the possibility of interaction with several components in the solution and will, on average, be more closely co-located favourably around perovskite precursor complexes. We speculate that this arrangement of molecular components with cation/ligand exchange dynamics around Sn-centred complexes will delay their assembling into perovskite. Accordingly, the larger effects on Sn^2+^ over Pb^2+^ by amino acid salts make these functionalities ideal for modifying the environment of Sn species and colloidal components in solution to control their heterogeneous nucleation (Supplementary Fig. 6).

To corroborate the NMR results, we carry out density functional theory (DFT) calculations (Fig. 1c and Supplementary Fig. 6). When modelling PhA with two MAI and FAI species, the structures show interaction energies of −12.05 and −12.13 eV, respectively, mainly originating from the H-bonding interaction, as well as the electrostatic interaction. The interaction energy for PhA with SnI_2_ is −21.79 eV with the H-bonding and –C=O⋯Sn interactions manifested. The SnI_2_•DMSO shows an interaction energy of −22.56 eV, suggesting a strong complexing. In SnI_2_ + PhA, two I atoms show distances of 2.854 and 2.935 Å to the Sn centre, with I_2_ slightly deflected by the sterically confined ammonium group of the amino acid terminal, decreasing the electron density on Sn. We further calculate the electrostatic potentials of the SnI_2_•DMSO and SnI_2_ + PhA species and estimate that the electrostatic potential of the Sn in SnI_2_ + PhA is more positive compared with the SnI_2_•DMSO adduct, suggesting a reduced electron density of Sn. Accordingly, these results rationalize the downfield shift of the ^119^Sn NMR with the addition of amino acid salt, ammonium, as well as carboxyl into the precursor solutions.

We conduct dynamic light scattering (DLS) measurements to study the colloidal properties of precursor solutions (Fig. 1d). We observe separate hydrodynamic size distributions that originate from differences in size-dependent Brownian motion in all cases. For the control solution, the colloidal particles evolve to a slightly increased size in the first 30 min and then the distribution of particle sizes widens. For the PhA solution, we observe no considerable size increase and/or change in distribution variations in the first 30 min. From 30 to 60 min, the solution notably evolves into a system dominated by particles with a narrow distribution of larger sizes, and it becomes stable from 60 to 120 min. By contrast, the ammonium PEA solution maintains a broadened distribution of particle sizes except for the distinctive emergence of narrow distributions at 30-min and 90-min points. For the PPA system, we observe a maintained largely broadened size distribution of the colloidal particles in the first 90 min, followed by a substantially narrowed size distribution from 120 min. For the PEAPPA solution, we observe broadened size distributions throughout the entire period, with a narrow distribution occurring only at 30 min. We conclude that the stable chemical interactions built in the solution system between the amino acid salt and precursor materials, especially Sn(ii)-based species, manifestly contribute to the colloidal particle stability and homogeneity, subsequently benefiting the nucleation.

## Morphology, crystallinity and electronic properties

**Fig. 2:**
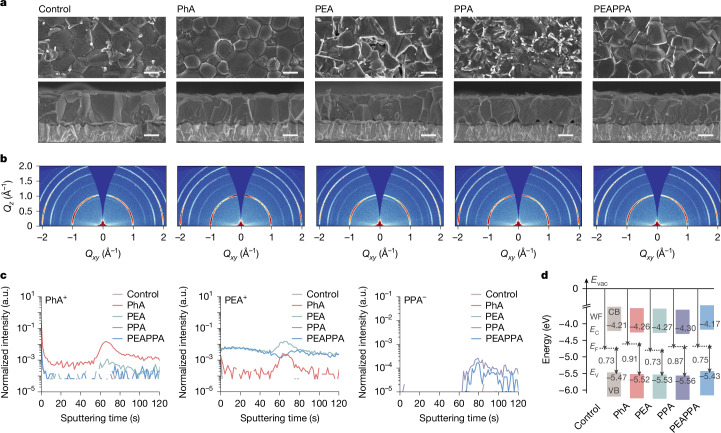
Morphology, crystallinity and electronic properties of Sn–Pb perovskite films. **a**, Top view and cross-sectional SEM images of control, PhA, PEA, PPA and PEAPPA perovskite films fabricated on PEDOT:PSS-coated FTO glass substrates. Scale bars, 500 nm. **b**, 2D GIWAXS patterns examined at a grazing incidence angle of 0.4° for perovskite films. The halo at the scattering vector of 1 and 2 Å^−1^ comes from the diffraction of (100) and (200) peaks, respectively. The patterns are presented on the same scale. The partial scattering ring at *q* = 0.9 Å^−1^ originates from residual PbI_2_ in films. The corresponding 1D XRD patterns are also provided in Supplementary Fig. 13. **c**, ToF-SIMS depth profile of PhA^+^, PEA^+^ and PPA^−^ ions for control and modified films. **d**, Energy-level diagram of perovskite films. Fermi level (*E*_F_) and valence band (VB) maximum (*E*_V_) are shown aligned at the vacuum level (*E*_vac_). Conduction band (CB) minimum (*E*_C_) is calculated from *E*_V_ + *E*_gap_ (bandgap, 1.26 eV). The UPS spectra are given in Supplementary Fig. 22. a.u., arbitrary units.

As observed in scanning electron microscopy (SEM) images, some deposits are visible between the perovskite grain boundaries of the control films (Fig. 2a), which are associated with SnF_2_ deposits^39^ and are less prominent in PhA films. From the cross-sectional view, both the control and PhA films mostly maintained monolithic grains with an average thickness of 850 ± 40 and 860 ± 80 nm, respectively (Supplementary Fig. 7). We further investigate minor variations in amino acid structure and deposit films with 4-fluoro-phenylalanine hydrochloride (4FPhA) and 4-methyl-phenylalanine hydrochloride (4MePhA). In general, we observe similar phenomena as PhA (Supplementary Figs. 8–10), indicating a generic role the amino acid salt plays on the morphology of perovskite films, with only weak influence from extra non-protic substituents on the phenyl core. Notably, the exposure of different facets of perovskite grains seems to occur on increasing the concentration of amino acid salts, which we interpret to be caused by changed facet surface energy, suggesting the successful attachment of molecules to grain surfaces.

The PEA films form dendritic structures at the top surfaces with increased heterogeneity and ‘wrinkled’ surfaces, resulting in increased thickness deviation, 950 ± 450 nm (Fig. 2a and Supplementary Fig. 11). For PPA films, we observe a considerably increased number of bright deposits at surfaces primarily along grain boundaries. Furthermore, we find ‘meso-voids’ at buried interfaces, despite maintaining good thickness uniformity, 870 ± 60 nm. Notably, as viewed by the eye from the glass side of films, these voids are apparent as a ‘haziness’ to the substrate (Supplementary Fig. 12). With a mixture of ammonium and carboxyl, PEAPPA, more needle-shaped species are formed and monolithic grains are less well maintained, with less evident voids at the bottom region and fewer hazy spots, giving a thickness of 940 ± 190 nm. These results indicate that the ammonium and carboxyl moieties in the single molecule led to superior film homogeneity and morphology compared with films modified solely with the ammonium or carboxyl as well as their mixture.

We conduct one-dimensional (1D) X-ray diffraction (XRD) and two-dimensional (2D) grazing-incidence wide-angle X-ray scattering (GIWAXS) measurements (Fig. 2b and Supplementary Fig. 13). From the XRD results, we observe greater than threefold enhancement in the (100) peak intensity of PhA films compared with the control. The PEA films show a similar peak intensity as the control, whereas the PPA film is close to that of the PhA. The PEAPPA films show a slightly reduced peak intensity compared with PPA films but substantially higher than the control. Interrogating the 2D GIWAXS results, we find that the PhA films exhibit reflections in concentrated regions of the q-space as compared with the control. With the sole addition of ammonium, the PEA films show a slightly enhanced scattering at the direction of about 30° out of the Qxy plane. However, the carboxyl gives the films a preferential orientation in both in-plane and out-of-plane directions. This leads us to conclude that the carboxyl group plays a critical structure-directing role in modifying the growth of perovskite crystals, whereas the ammonium contributes weakly.

We use time-of-flight secondary-ion mass spectrometry (ToF-SIMS) to track the distribution of additive ions, that is, PhA^+^, PEA^+^ and PPA^−^ (Fig. 2c). The results show that introduced PhA^+^ cations are mainly distributed at the perovskite surface regions, with a particular evident accumulation at the buried interface. By contrast, PEA^+^ cations are found to be much more homogeneously distributed. Note that the PEA detected from PhA films is most probably attributed to PhA fragments without –COOH, caused by the primary ion beam excitation. Notably, we observe a higher intensity from the PPA molecule at the bottom region of PPA-containing films, whereas the detection limit of this technique means that we cannot exclude the presence of the molecule at grain boundaries and top surfaces. We infer that the preferential accumulation of amino acid ligands at the bottom region of films may be governed by the carboxyl moiety of molecules with less contribution from the ammonium.

We have performed DFT calculations to study the possible defect passivation effect (Supplementary Figs. 14–21). When the PhA molecule is applied to iodide vacancies centred with Sn and Pb, that is, V_I(Sn)_ and V_I(Pb)_, the systems are stabilized with energies of −1.60 (V_I(Sn)_) and −1.74 eV (V_I(Pb)_). The newly established Sn–O and Pb–O covalent bonds give lengths of 2.905 and 3.039 Å, respectively. The substitution of –F and –CH_3_ in PhA slightly increases resultant binding energies. PhA can also coordinate with A-site vacancies (V_(A)_) by means of the ammonium group, with energies of about −1.28 eV. Similarly, the PEA and PPA molecules also show individual ability to passivate A-site and X-site vacancies. Apart from further stabilization with PhA, as for the single V_I(Sn/Pb)_ defect, the bulky amino acid terminal of PhA slightly deforms the lattice. Thus, we construct a two-defect system, containing one A-site vacancy accompanied by one adjacent iodide vacancy. PhA^+^ is modelled to the A-site vacancy while the undercoordinated Sn/Pb atoms form ionic bonding with chloride from PhA, enabling considerably decreased energies ranging from −2.27 to −2.80 eV. This suggests that these two-defect systems can be greatly stabilized by PhA and the substitution effect of –F and –CH_3_ is like the trend observed in single-defect systems. The superior passivation effect of amino acid salts largely originates from the numerous behaviours of functional units, which allow these molecules to passivate both donor and acceptor types of defect in a mutually reinforced manner. The effects are also reflected in other film electronic properties (Fig. 2d, Supplementary Figs. 22 and 23 and Supplementary Table 1).

## Optoelectronic properties

**Fig. 3:**
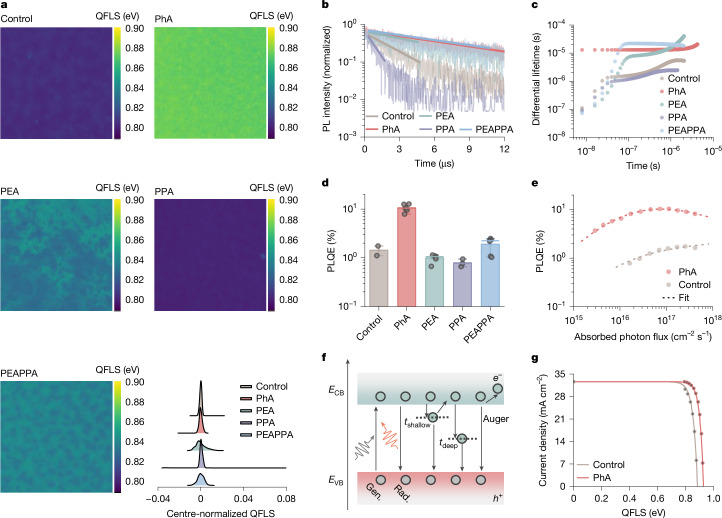
Optoelectronic property characterizations of Sn–Pb perovskite films. **a**, QFLS mapping calculated from 2 mm × 2 mm PLQE images for the perovskite films recorded at *V*_OC_ conditions under illumination (equivalent to 1-sun intensity for a 1.26-eV bandgap) with the set-up reported previously^47^. The centre-normalized QFLS are plotted to evaluate spatial QFLS variations. **b**, TRPL of the films. The samples are illuminated from the glass side with a 635-nm centre wavelength laser (4.9 nJ cm^−2^ pulse^−1^). **c**, The differential lifetime *τ*_diff_ (*t*). **d**, PLQE of different perovskite films illuminated with a 532-nm laser at a 1-sun equivalent intensity for a 1.26-eV bandgap. Data are mean ± s.e.m. **e**, Intensity-dependent PLQE of control and PhA films. **f**, Generation and recombination pathways in a two-trap level SRH model. *t*_shallow_ and *t*_deep_ represent shallow-level and deep-level carrier trapping states, respectively. The detrapping and Auger processes are also presented. **g**, Pseudo *J*–*V* curves reconstructed from intensity-dependent PLQE measurements.

We map out the quasi-Fermi level splitting (QFLS) by calculating the imaged absolute photoluminescence quantum efficiency (PLQE; Fig. 3a). Compared with the control, the PhA films present substantially increased QFLS over the entire examined region. For PEA films, the image suggests an enhanced QFLS, whereas the homogeneity is worse as quantified by the broadening of the peak in centre-normalized QFLS plots. The PPA films show reduced QFLS values but a comparable QFLS homogeneity with the control. When the film is modified with a mixture of ammonium and carboxyl, PEAPPA, we observe increased heterogeneity compared with the control, despite an improvement in QFLS, indicating a greater variation in the optoelectronic quality.

The interpretation of photoluminescence (PL) transient decay curves gives PL lifetimes of 2.2, 9.5, 6.5, 0.7 and 9.4 µs for control, PhA, PEA, PPA and PEAPPA films, respectively (Fig. 3b). The PEAPPA films show an ultra-long lifetime similar to that of PhA. Extremely long PL lifetimes have historically been interpreted to indicate very low amounts of trap-assisted recombination and hence defect densities. As recently highlighted^40^, however, ultra-long PL lifetimes probably originate from a population of carriers trapped in shallow-level defect states, which cause delayed PL when released from traps back into the bands. These carriers are expected to dominate the population in Sn–Pb perovskites after photoexcitation^41^, making the single lifetime value less physically meaningful. We then calculate the differential lifetime (*τ*_diff_) from the time-resolved photoluminescence (TRPL) decays^42,43^ (Fig. 3c). After the pulse, we observe a fast increase in *τ*_diff_ for control, PEA, PPA and PEAPPA films. By contrast, the *τ*_diff_ remains relatively constant for PhA films, with no initial short-lifetime component. Higher-order recombination processes such as Auger or radiative recombination are unlikely to greatly contribute to this difference in early-time *τ*_diff_, as we apply a low laser fluence for measurements and do not expect modified films to have markedly different high-order recombination rates. Instead, the lack of faster initial recombination for PhA films would be consistent with reduced trap-filling at the start of the decay, which could be because of either a lower density or carrier capture cross-section of traps. The PPA films show similar *τ*_diff_ evolution as the control, whereas PEAPPA films are closer to PEA films with a longer decay time. This suggests that the PEA predominantly governs the optoelectronic property of PEAPPA films.

We conduct macroscopic PLQE measurements under 1-sun equivalent intensity (Fig. 3d). The best PLQE value for PhA films is 12.7%, with an average value of 10.6 ± 1.35%, consistent with the reduced trap-assisted recombination suggested by TRPL results. By contrast, the best PLQE for control, PEA, PPA and PEAPPA films are about one order of magnitude smaller, with values of 1.75%, 1.14%, 0.94% and 2.48%, respectively.

We fit the PLQE results measured at a range of excitation intensities with a Shockley–Read–Hall type model with one shallow and one deep electron trap^44^ (Fig. 3e,f) as a function of absorbed photons, allowing *k*_trap,shallow_ and *k*_trap,deep_ to vary between two samples (Supplementary Table 2). On the basis of fitting, the *k*_trap,shallow_ values decrease from 2.3 × 10^6^ to 9.6 × 10^4^ s^−^^1^ and the *k*_trap,deep_ values decrease from 4.7 × 10^4^ to 1.4 × 10^4^ s^−^^1^ comparing PhA samples with control. We expect this reduction in trapping rate to result from the reduced density of both deep and shallow defects. This is consistent with the constant and high *τ*_diff_ observed in PhA films, characteristic of both reduced trap-filling at the beginning of the TRPL decay and reduced emission from shallow traps at later times of the decay, with the latter inferred from the smaller *τ*_diff_ than some of the other samples at longer times.

Using intensity-dependent PLQE results, we generate pseudo current density–voltage (p*J*–*V*) curves (Fig. 3g). The curves suggest that PhA-films-based devices could generate a pseudo-*V*_OC_ of 0.93 V (94% of the radiative limit at 1.26-eV bandgap^45^) and a pseudo-fill factor (FF) of 0.87 (p*V*_OC_ × pFF is about 93% of the limit), which are considerably higher than the values of the control with p*V*_OC_ of 0.88 V and pFF of 0.85. Assuming a generally achievable current density obtained from actual cells, 32.5 mA cm^−^^2^, the pseudo-PCE for control and PhA devices is 24.7% and 26.5%, respectively.

## PV device performance

**Fig. 4:**
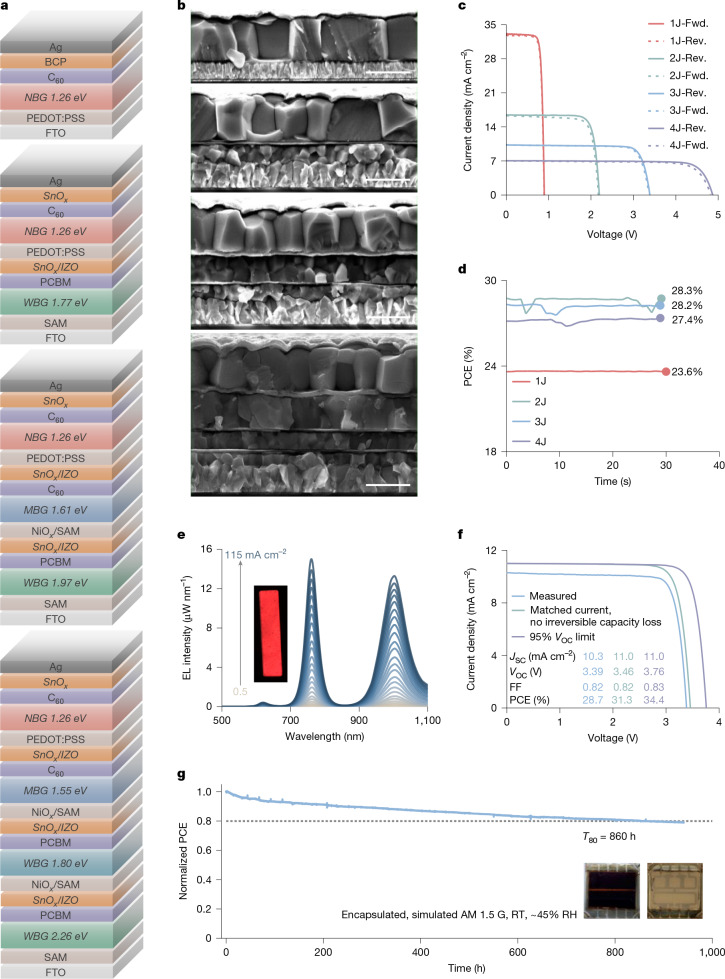
Solar cell devices. **a**, Device configuration (top to bottom). **b**, Cross-sectional SEM images (top to bottom). Scale bars, 1 µm. *J*–*V* curves (**c**) and the steady-state output (**d**) of optimized single-junction (1J), double-junction (2J), triple-junction (3J) and quadruple-junction (4J) cells. ‘Rev.’ and ‘Fwd.’ represent reverse (*V*_OC_ to *J*_SC_) and forward (*J*_SC_ to *V*_OC_) *J*–*V* scan directions, respectively. Details of the device stacks are provided in Methods. The performance for the 0.25-cm^2^ tandem cells is shown here, whereas the results for 1-cm^2^ tandems are provided in Supplementary Fig. 41. **e**, EQE_EL_ spectra of one representative triple-junction cell measured as a function of injected current density ranging from 0.5 to 115 mA cm^−2^. The emission peaks of WBG (1.97 eV), MBG (1.61 eV) and NBG subcells are centred at about 620, 762 and 1,005 nm, respectively. Inset, photograph of the emission from a 1-cm^2^ cell under a forward bias of about 3.5 V. **f**, Experimental and reconstructed *J*–*V* curves of the triple-junction device from the simulated EQE at different optical conditions and assuming that a *V*_OC_ of 95% detailed-balance limit is achieved in each subcell. **g**, MPPT stability of an encapsulated triple-junction device, measured in ambient air at room temperature (RT) with a relative humidity (RH) of about 45% under simulated AM 1.5 G illumination. Insets, photographs of the aged cell with the top contact of Cr (2.5 nm)/Au (20 nm)/Ag (100 nm). The initial PCE of the triple-junction cells is 26.5%, with *J*–*V* curves provided in Supplementary Fig. 59.

We fabricate single-junction devices with structures shown in Fig. 4a,b. We first examine the effect and generic role of amino acid salts, that is, PhA, 4FPhA and 4MePhA, with accordingly modified PV devices. Compared with control devices, the amino-acid-salt-based cells show consistently improved PCEs that mainly originate from the boosted *V*_OC_ (Supplementary Figs. 24–29), benefiting partially from slightly suppressed sub-bandgap states (Supplementary Fig. 30). Also, PhA devices outperform PEA, PPA and PEAPPA cells (Supplementary Figs. 31 and 32). We thus obtain the best PCE value of 23.9% from the forward *J*–*V* scan (*V*_OC_: 0.90 V, *J*_SC_: 33.07 mA cm^−2^, FF: 0.80), with the best *V*_OC_ of 0.91 V (Fig. 4c,d and Supplementary Figs. 33–36) and steady-state PCE of 23.4% (Supplementary Fig. 37). The highest external quantum efficiency (EQE) value of PhA devices reaches 92.5% at approximately 680 nm (Supplementary Fig. 38), with an integrated *J*_SC_ of 32.7 mA cm^−2^. Under ‘shelf-storage’ conditions (International Summit on Organic and Hybrid Photovoltaics Stability (ISOS)-D-1)^46^ (Supplementary Fig. 39), the unencapsulated control, PhA, 4FPhA and 4MePhA devices maintain 82%, 84%, 87% and 93% of their initial performances, respectively, after 2,200 h.

We fabricate double-junction and triple-junction devices after optimizing a series of neat Pb perovskites. Schematics of the precise layers used in devices and their SEM images are shown in Fig. 4a,b. Device parameters for the champion cells are provided in Table 1. When scanned from *V*_OC_ to *J*_SC_, the double-junction cells show PCE values of up to 29.2% and 28.4% for 0.25-cm^2^ and 1-cm^2^ devices, respectively (Fig. 4c,d and Supplementary Figs. 40 and 41). The best *V*_OC_ obtained for this type of device is 2.22 V, close to the summed *V*_OC_ of single-junction perovskite solar cells, 2.25 V (Supplementary Figs. 42 and 43). The *J*_SC_ estimated by integrating EQE spectra is 16.5 and 15.9 mA cm^−2^ for wide-bandgap (WBG) and NBG subcells, respectively (Supplementary Fig. 44). For triple-junction cells, the 0.25-cm^2^ and 1-cm^2^ devices also show high PCE values of up to 28.7% and 28.4% (27.28% certified by the Japan Advanced Institute of Science and Technology (AIST), respectively, with the best *V*_OC_ of 3.46 V, slightly lower than the summed voltage 3.54 V (Fig. 4c,d and Supplementary Figs. 41 and 45). The *J*_SC_ estimated by integrating the EQE spectra is 11.5, 9.1 and 10.5 mA cm^−^^2^ for the WBG, middle-bandgap (MBG) and NBG subcells, respectively (Supplementary Fig. 46). Our measured *J*_SC_ of 10.3 mA cm^−2^ is extremely close to the value of 10.2 mA cm^−^^2^ from the AIST, giving a great level of confidence in the accuracy of our in-house measurements. We note that our Sn–Pb perovskite fabrication recipe is also highly reliable and reproducible (Supplementary Figs. 47 and 48), having been reproduced in laboratories at Oxford, Kyoto and Wuhan. Double-junction cells fabricated in Wuhan have been verified by an external laboratory (SIMIT, China), demonstrating a certified PCE of 29.76% (steady-state 29.26%; Supplementary Fig. 49). As a proof-of-concept, the 0.25-cm^2^ and 1-cm^2^ quadruple-junction devices show PCEs of up to 27.9% and 27.4%, respectively, with the best *V*_OC_ of 4.94 V (Fig. 4c,d and Supplementary Figs. 41, 50 and 51). Compared with previous reports, our tandem cells demonstrate superior efficiencies across all fabricated junctions, particularly when accounting for our large cell area (Supplementary Fig. 52).

**Table 1:** PV parameters of the best-performing multijunction cells

Cells	Device area (cm^2^)	Scan direction^a^	*V*_OC_ (V)	*J*_SC_ (mA cm^−^^2^)	FF	PCE (%)	Steady-state PCE (certified, %)^b^	Best *V*_OC_ (V)
Double junction	0.25	Rev.	2.20	16.4	0.81	29.2	28.3	2.22
		Fwd.	2.18	16.3	0.78	27.7		
	1	Rev.	2.18	16.6	0.79	28.4	28.2	2.21
		Fwd.	2.16	16.5	0.77	27.3		
Triple junction	0.25	Rev.	3.39	10.3	0.82	28.7	28.2	3.46
		Fwd.	3.37	10.3	0.81	28.2		
	1	Rev.	3.42	10.3	0.81	28.4	28.0 (27.28)	3.46
		Fwd.	3.41	10.3	0.79	28.0		
Quadruple junction	0.25	Rev.	4.87	7.0	0.81	27.9	27.4	4.94
		Fwd.	4.83	7.0	0.77	26.3		
	1	Rev.	4.90	7.6	0.73	27.4	26.9	4.90
		Fwd.	4.86	7.6	0.72	26.8		

^a^‘Rev.’ and ‘Fwd.’ represent reverse (*V*_OC_ to *J*_SC_) and forward (*J*_SC_ to *V*_OC_) *J*–*V* scan directions, respectively.

^b^The certified PCE of double-junction cells with the 0.0498-cm^2^ active area is 29.26%.

We select triple-junction cells to measure the electroluminescence external quantum efficiency (EQE_EL_) (Fig. 4e, Supplementary Fig. 53 and Supplementary Table 3). Under an injection current density of about 9.4 mA cm^−2^, the device shows EQE_EL_ of up to 1.95%, with values of 0.01%, 0.48% and 1.46% from 1.97-eV, 1.61-eV and 1.26-eV subcells, respectively. Notably, the EL-derived QFLS values are very close to those measured for individual single-junction and integrated triple-junction devices, suggesting that only a small *V*_OC_ loss of about 30 mV is introduced in interconnecting layers of the multijunction device stack. With this *V*_OC_ loss omitted and a matched current, the triple-junction cells should generate greater than 31% PCEs (Fig. 4f and Supplementary Figs. 54–57). The potential PCE of triple-junction devices can be greater than 34% under the context of a successful gain of 95% radiative voltage of each subcell. We note that our 1.26-eV, 1.55-eV, 1.61-eV, 1.77-eV and 1.80-eV cells achieve more than 90% of the *V*_OC_ radiative limit (Supplementary Fig. 58). Although 1.97-eV and 2.26-eV cells show record-low voltage losses, they remain more loss than narrower-gap junctions and hence should be the future focus.

We conduct MPPT at room temperature (ISOS-L-1)^46^ (Fig. 4g and Supplementary Fig. 59), giving a *T*_80_ of 860 h for triple-junction cells. When the cells are stressed under 85 °C in dark conditions (ISOS-D-2)^46^, they exhibit reasonably good stability, maintaining about 70% on average of their initial efficiencies after more than 1,200 h (Supplementary Figs. 60 and 61). However, when cells are stressed under more accelerated conditions, 85 °C and 1 sun at *V*_OC_ (ISOS-L-2)^46^, we observe a rapid degradation, with *T*_80_ as short as 5 h. These results indicate that, although our multijunction cells are reasonably stable operating at ambient conditions and when heated in the dark, further improvements are required to reach suitable stability under combined temperature and light. Overall, our work represents a milestone, in which large-area triple-junction cells closely match the efficiency of double-junction cells. We expect triple-junction or quadruple-junction cells to lead the efficiency race for thin-film tandem devices shortly.

## Methods

### Materials

Unless otherwise stated, all materials were used as received without further purification. Methylammonium iodide (MAI, >99.0%), formamidinium iodide (FAI, >98.0%) and formamidinium bromide (FABr, >99.99%) were purchased from Greatcell Solar Materials. Bathocuproine (BCP, >99.0%), lead iodide (PbI_2_, 99.99%, trace metals basis), lead(ii) bromide (PbBr_2_, >98.0%), lead(ii) chloride (PbCl_2_, >99.5%), methylamine hydrobromide (MABr, >98%), methylamine hydrochloride (MACl, >98.0%), piperazine (anhydrous, PP, >98.0%), l-phenylalanine (Phe, >98.0%), 4-methyl-phenylalanine (4MePhe, >98.0%), 2-phenylethylamine hydrochloride (PEA, >98.0%), PPA (>98.0%), [2-(3,6-dimethoxy-9H-carbazol-9-yl)ethyl]phosphonic acid (MeO-2PACz), 2-phenylethylamine hydroiodide (PEAI, >98.0%) and hydrochloride acid solution (35–37 wt% in water) were purchased from Tokyo Chemical Industry Co., Ltd. (TCI). Caesium iodide (CsI, 99.999%, metals basis) was purchased from Alfa Aesar. Ammonium thiocyanate (NH_4_SCN, 99.99% trace metals basis), tin fluoride (SnF_2_, 99%), tin iodide (SnI_2_, beads, 99.99%, trace metals basis), Sn(0) powder (<45 μm particle size, 99.8% trace metals basis), 4-fluoro-d-phenylalanine hydrochloride (4FPhA, 99%), C_60_ pyrrolidine tris-acid (97%), propane-1,3-diammonium iodide (PDAI_2_, >98%), poly(ethyleneimine) solution (PEI, 50 wt% in H_2_O) and poly(methyl methacrylate) (PMMA, >98%) were purchased from Sigma-Aldrich Co., Ltd. (Sigma-Aldrich). Poly(3,4-ethylenedioxythiophene):poly(styrene sulfonate) (PEDOT:PSS) aqueous solution (Clevios PVP AI 4083) was purchased from Heraeus Co., Ltd. Fullerene C_60_ (sublimed, 99.99%) was purchased from ATR Company. [6,6]-Phenyl-C_61_-butyric acid methyl ester (PC_61_BM, >99%) was purchased from Ossila. (2-(4-(bis(4-methoxyphenyl)amino)phenyl)-1-cyanovinyl)phosphonic acid (MPA-CPA) and (4-(3,6-diiodo-9H-carbazol-9-yl)butyl)phosphonic acid (I-4PACz) were purchased from Dyenamo AB. NiO_*x*_ nanoparticles were purchased from Avantama. Dehydrated dimethylsulfoxide (DMSO) and propan-2-ol (IPA) were purchased from FUJIFILM Wako Pure Chemical Co., Ltd. or Sigma-Aldrich. Dehydrated *N,N*-dimethylformamide (DMF), toluene, anisole and chlorobenzene were purchased from Kanto Chemical Co., Inc. or Sigma-Aldrich. Methanol and diethyl ether for synthesis were purchased from FUJIFILM Wako Pure Chemical Co., Ltd. or Nacalai Tesque, Inc. All of these solvents were degassed by Ar gas bubbling for 1 h and further dried with molecular sieves (3 Å) in an Ar-filled (at Kyoto) glovebox (H_2_O, O_2_ < 0.1 ppm) before use. No treatment was applied for the solvents used at Oxford. Tetrakis(dimethylamino) tin(iv) was purchased from Pegasus Chemicals Limited. Indium zinc oxide (IZO) and SiO_2_ sputtering targets (99.995%) were purchased from Angstrom Engineering.

### Synthesis of amino acid salts

PhA was prepared by dissolving 2.5 g (15 mM) of Phe in 50 ml of methanol. While the solution was being stirred, 3.0 g (2.0 eq, 30 mM) of hydrochloride acid solution (35–37 wt% in water) was added. The solution was stirred for 1 h at 0 °C to ensure a complete reaction and, subsequently, the solvent was removed using a rotary evaporator. Purification was accomplished by recrystallization in methanol and diethyl ether, affording PhA white powder (2.38 g, yield 78%). The resulting PhA powder was dried at 70 °C under vacuum for 4 h and then transferred to an Ar-filled or N_2_-filled glovebox. The synthesis procedure and the product yield of 4MePhA are similar to that of PhA described above.

The left-handed configuration was mainly used in the study, as the chirality of the amino acid showed no considerable impact on the device performance in our study, whereas some interesting chiral spacers may determine the packing configuration of low-dimensional perovskite units that could affect the device performance^48^. We also note that the amino acid in a zwitterionic form could also work for improving the *V*_OC_ of the PV devices, whereas the FF shows lower values than the case using the protonated amino acid, that is, amino acid salts. Also, the solubility of the amino acid is much lower than the amino acid salts in most of the solvents, for example, DMSO and DMF, and even the solvent with high polarity, for example, water, because of the strong intermolecular H-bonding and ionic attractions generated between the –COO^−^ and –NH_3_^+^ terminals of the amino acid. We thus recommend using amino acid salts but not amino acids in their unprotonated zwitterionic form. The sterically confined configuration and the distance between the ammonium and carboxylic acid groups in the amino acid salts is also critical, as a long distance between them could lead to the formation of low-dimensional perovskite phases with the ammonium group intercalating into the lattice and the whole molecule serving as a quantum wall spacer. Low-dimensional perovskite phases show a larger insulating nature than the 3D perovskites, decreasing the carrier mobility of the 3D perovskite-dominated films applied for the PV cells.

On the basis of previous studies^49–52^, modifiers with the aromatic moiety should, in general, outperform the modifiers with the aliphatic moiety. We thus select PhA to extend our previous glycine hydrochloride research^38^. Also, using PhA as the amino acid salt allows us to compare with the ammonium-only analogue PEA—one of the most applied additives in the perovskite research—in our Sn–Pb perovskites and the carboxylic-acids-only analogue, PPA, to decouple the role of ammonium and carboxylic acid moieties in the amino acid salt additives.

### Fabrication of perovskite thin films

#### 1.26-eV bandgap mixed tin–lead perovskites

The perovskite film was prepared in an Ar-filled (at Kyoto) or N_2_-filled (at Oxford) glovebox (H_2_O, O_2_ < 0.1 ppm). The Cs_0.1_FA_0.6_MA_0.3_Sn_0.5_Pb_0.5_I_3_ perovskite precursor solution was prepared by mixing CsI (48.06 mg, 0.185 mmol), FAI (190.89 mg, 1.11 mmol), MAI (88.23 mg, 0.555 mmol), SnI_2_ (344.58 mg, 0.925 mmol), PbI_2_ (426.43 mg, 0.925 mmol), SnF_2_ (14.50 mg, 0.0925 mmol) and NH_4_SCN (2.82 mg, 0.037 mmol) in a solvent mixture of 0.25 ml DMSO and 0.75 ml DMF to reach a concentration of 1.85 M for the control samples. 0.5 mg ml^−1^ Sn(0) powder was added to the perovskite precursor solution to scavenge the potential Sn(iv) introduced by oxidation of Sn(ii)^53^. For the PhA samples, 3 mol% of PhA (11.19 mg, 0.0555 mmol) was added to the control materials. 3 mol% of PEA (8.75 mg, 0.0555 mmol) was added to the control materials for the PEA samples. For the PPA samples, 3 mol% of PPA (8.33 mg, 0.0555 mmol) was added to the control materials. For the PEAPPA samples, 3 mol% of PEA (8.75 mg, 0.0555 mmol) and PPA (8.33 mg, 0.0555 mmol) were added to the control materials. The precursor solutions were stirred at 45 °C for about 40 min and filtered through a 0.20-μm PTFE filter before use. To spin-coat the films, 200 μl of the room-temperature precursor solution was applied to the substrate. A two-step spin-coating programme was used. The first step was 1,000 rpm for 10 s with an acceleration of 200 rpm s^−1^ and the second was 4,000 rpm for 40 s with a ramp-up of 1,000 rpm s^−1^. Room-temperature chlorobenzene (400 and 500 μl for 25 × 25 mm^2^ (at Kyoto) and 30 × 30 mm^2^ (at Oxford) substrates, respectively) was used as the antisolvent. The chlorobenzene was quickly dripped onto the surface of the spinning substrate over an interval of 1 s during the second spin-coating step 20 s before the end of the procedure. The substrate was then immediately annealed on a 100 °C hotplate for 10 min, followed by annealing at 65 °C for more than 10 min to avoid glovebox vapour ingress of the as-prepared films, then the films were cooled to room temperature for the following processes. The post-treatment was done by dripping the PPCPTA (piperazine and C_60_ pyrrolidine tris‐acid) solution dynamically while spinning the as-prepared perovskite films, an approach we developed previously^54^. The spin-coating process was set at 4,000 rpm for 20 s with an acceleration of 1,333 rpm s^−1^. Following spin-coating, the films were immediately annealed again at 100 °C for about 5 min.

#### 1.55-eV bandgap neat lead perovskites

For the hole-selective layer, NiO_*x*_ (diluted in 1:10 ethanol, v:v) was spin-coated in the air at 3,000 rpm for 30 s (3 s acceleration to 3,000 rpm) without post-treatment. The hole-selective layer was deposited with the mixture of MeO-2PACz (0.25 mg ml^−1^) and I-4PACz (0.25 mg ml^−^^1^) solution in IPA by spin-coating inside an N_2_-filled glovebox and the substrates were annealed at 105 °C for 10 min. To deposit the perovskite film with the main composition of Cs_0.05_FA_0.90_MA_0.05_PbI_2.95_Br_0.05_, 1.5 M perovskite precursor solution was prepared by mixing CsI, FAI, MABr and PbI_2_ in DMF:DMSO (4:1, v:v) mixed solvent subject to the stoichiometric ratio. Further 2 mol% PbI_2_ and 5 mol% MACl were added to the precursor as additives for better film quality^55^. The perovskite precursor (200 μl) was spin-coated at 4,000 rpm for 50 s (5 s acceleration to 4,000 rpm). 270 μl anisole was dropped on the film 20 s before the end of the spinning programme. The film was immediately annealed at 105 °C for 20 min. For the interfacial passivation layer, a solution, with 0.5 mg EDAI_2_ and 0.5 mg PEAI added to 1 ml IPA and chlorobenzene (v:v, 1:1) mixed solvent, was spin-coated onto the as-prepared perovskite films and followed up with 3 min of thermal annealing under 105 °C.

#### 1.61-eV bandgap neat lead perovskites

For the hole-selective layer, NiO_*x*_ (diluted in 1:10 ethanol, v:v) was spin-coated in the air at 3,000 rpm for 30 s (3 s acceleration to 3,000 rpm) without any post-treatment. 1 mg ml^−^^1^ MPA-CPA solution in ethanol was spin-coated inside an N_2_-filled glovebox at 3,000 rpm for 30 s (3 s acceleration to 3,000 rpm) and the substrates were annealed at 100 °C for about 10 min. To deposit the perovskite film with the main composition of Cs_0.1_FA_0.9_Pb(I_0.85_Br_0.15_)_3_, 1.5 M perovskite precursor solution was prepared by mixing CsI, FAI, PbBr_2_ and PbI_2_ in DMF:DMSO (4:1, v:v) mixed solvent subject to the stoichiometric ratio. The perovskite precursor (200 μl) was spin-coated at 1,000 rpm for 10 s (2 s acceleration to 1,000 rpm) and 5,000 rpm for 30 s (2 s acceleration to 5,000 rpm). 160 μl anisole was dropped on the film 5 s before the end of the spinning programme. The film was immediately annealed at 100 °C for 10 min. For the interfacial passivation layer, a solution, with 0.5 mg EDAI_2_ and 1 mg PEAI added to 1 ml IPA, was spin-coated onto the as-prepared perovskite films and followed up with 5 min thermal annealing under 100 °C.

#### 1.77-eV bandgap neat lead perovskites

For the hole-selective layer, NiO_*x*_ (diluted in 1:10 ethanol, v:v) was spin-coated in the air at 3,000 rpm for 30 s (3 s acceleration to 3,000 rpm) without any post-treatment. 1 mg ml^−^^1^ MPA-CPA solution in ethanol was spin-coated inside an N_2_-filled glovebox at 3,000 rpm for 30 s (3 s acceleration to 3,000 rpm) and the substrates were annealed at 100 °C for about 10 min. To deposit the perovskite film with the main composition of Cs_0.2_FA_0.8_Pb(I_0.6_Br_0.4_)_3_, 1.2 M perovskite precursor solution was prepared by mixing CsI, FAI, PbBr_2_ and PbI_2_ in DMF:DMSO (4:1, v:v) mixed solvent subject to the stoichiometric ratio. The perovskite precursor (200 μl) was spin-coated at 4,000 rpm for 32 s (4 s acceleration to 4,000 rpm). 160 μl anisole was dropped on the film 8 s before the end of the spinning programme. The film was immediately annealed at 100 °C for 15 min. For the interfacial passivation layer, a solution, with 0.5 mg EDAI_2_ and 1 mg PEAI added to 1 ml IPA, was spin-coated onto the as-prepared perovskite films and followed up with 5 min thermal annealing under 100 °C.

#### 1.80-eV bandgap neat lead perovskites

For the hole-selective layer, NiO_*x*_ (diluted in 1:10 ethanol, v:v) was spin-coated in the air at 3,000 rpm for 30 s (3 s acceleration to 3,000 rpm) without any post-treatment. 1 mg ml^−^^1^ MPA-CPA solution in ethanol was spin-coated inside an N_2_-filled glovebox at 3,000 rpm for 30 s (3 s acceleration to 3,000 rpm) and the substrates were annealed at 100 °C for about 10 min. To deposit the perovskite film with the main composition of Cs_0.15_FA_0.85_Pb(I_0.55_Br_0.45_)_3_, 1.2 M perovskite precursor solution was prepared by mixing CsI, FAI, PbBr_2_ and PbI_2_ in DMF:DMSO (4:1, v:v) mixed solvent subject to the stoichiometric ratio. The perovskite precursor (200 μl) was spin-coated at 1,000 rpm for 10 s (2 s acceleration to 1,000 rpm) and 5,000 rpm for 30 s (2 s acceleration to 5,000 rpm). 160 μl anisole was dropped on the film 5 s before the end of the spinning programme. The film was immediately annealed at 100 °C for 10 min. For the interfacial passivation layer, a solution, with 0.5 mg EDAI_2_ and 1 mg PEAI added to 1 ml IPA, was spin-coated onto the as-prepared perovskite films and followed up with 5 min thermal annealing under 100 °C.

#### 1.97-eV bandgap neat lead perovskites

For the hole-selective layer, NiO_*x*_ (diluted in 1:10 ethanol, v:v) was spin-coated in the air at 3,000 rpm for 30 s (3 s acceleration to 3,000 rpm) without any post-treatment. 0.5 mg ml^−^^1^ Me-4PACz solution in ethanol was spin-coated inside an N_2_-filled glovebox at 3,000 rpm for 30 s (3 s acceleration to 3,000 rpm) and the substrates were annealed at 100 °C for about 10 min. To deposit the perovskite film with the main composition of Cs_0.15_FA_0.85_Pb(I_0.33_Br_0.67_)_3_, 0.8 M perovskite precursor solution was prepared by mixing CsI, FAI and PbBr_2_ in DMF:DMSO (4:1, v:v) mixed solvent subject to the stoichiometric ratio. Further 0.5 mol% PDAI_2_, PbCl_2_ and MACl were added to the precursor as additives for better film quality, as we recently reported^20^. The perovskite precursor (200 μl) was spin-coated at 1,000 rpm for 10 s (2 s acceleration to 1,000 rpm) and 5,000 rpm for 30 s (2 s acceleration to 5,000 rpm). 170 μl anisole was dropped on the film 5 s before the end of the spinning programme. The film was immediately annealed at 100 °C for 10 min. For the interfacial passivation layer, a solution, with 0.5 mg EDAI_2_ and 1 mg PEAI added to 1 ml IPA, was spin-coated onto the as-prepared perovskite films and followed up with 5 min thermal annealing under 100 °C.

#### 2.26-eV bandgap neat lead perovskites

For the hole-selective layer, NiO_*x*_ (diluted in 1:10 ethanol, v:v) was spin-coated in the air at 3,000 rpm for 30 s (3 s acceleration to 3,000 rpm) without any post-treatment. 0.5 mg ml^−^^1^ Me-4PACz solution in ethanol was spin-coated inside an N_2_-filled glovebox at 3,000 rpm for 30 s (3 s acceleration to 3,000 rpm) and the substrates were annealed at 100 °C for about 10 min. To deposit the perovskite film with the main composition of FAPbBr_3_, 0.9 M perovskite precursor solution was prepared by mixing FABr and PbBr_2_ in DMF:DMSO (4:1, v:v) mixed solvent subject to the stoichiometric ratio. The perovskite precursor (200 μl) was spin-coated at 1,000 rpm for 10 s (2 s acceleration to 1,000 rpm) and 5,000 rpm for 30 s (2 s acceleration to 5,000 rpm). 160 μl anisole was dropped on the film 5 s before the end of the spinning programme. The film was immediately annealed at 100 °C for 10 min. For the interfacial passivation layer, a solution, with 0.5 mg EDAI_2_ and 1 mg PEAI added to 1 ml IPA, was spin-coated onto the as-prepared perovskite films and followed up with 5 min thermal annealing under 100 °C.

### Fabrication of solar cell devices

Unless otherwise stated, all of the vacuum-based depositions were processed with the National Thin-Film Cluster Facility for Advanced Functional Materials (NTCF) assembled by Angstrom Engineering Inc. The Picosun model R200 Advanced Plasma atomic layer deposition (ALD) chamber was connected and controlled by a main cluster operation system. The ALD system was vacuum-pumped by a two-stage Edwards iXH610 pumping system and the process pressure was usually maintained at 5 mbar. The background carrier gas for the ALD system was N_2_ (99.9999%). The ALD system was equipped with two room-temperature sources for liquid-based precursors (typically, deionized water and trimethylaluminium (TMA, Pegasus) were loaded) in Pico 100 containers and two heatable sources in Pico 200 (TDMASn, Pegasus) and Pico 300 (InCp, EpiValence) liquid-based precursor containers. 20 nm SnO_*x*_ was grown at the ALD chamber using the combination of the TDMASn and deionized water precursors with a pulse time set at 1.4 and 1.6 s for TDMASn(iv) and H_2_O, respectively. The SnO_*x*_ layer deposition for neat lead-based films was conducted under the reactor temperature of 100 °C for 150 cycles, whereas 85 °C and 120 cycles were used for the layer on the mixed Sn–Pb perovskites. Sputtering deposition of the IZO interconnection layer was conducted under the process pressure of 4 × 10^−^^3^ mbar. The flow rates of 18 and 0.4 sccm (standard cubic centimetres per minute) were applied for Ar and O_2_, respectively, to generate the plasma for deposition at room temperature. The plasma was ignited with DC power under a pulse frequency of 20 kHz. Sputtering deposition of the SiO_*x*_ layer was carried out under the process pressure of 4 × 10^−^^3^ mbar at room temperature using a SiO_2_ target with the flow of Ar at the rate of 18 sccm.

### Single-junction mixed tin–lead perovskite solar cells

Glass/FTO substrates (10 Ω sq^−1^, AGC Inc.) were etched with zinc powder and HCl (6 M in deionized water) and consecutively cleaned for 15 min in an ultrasonic bath in water, acetone and detergent solution (Semico Clean 56, Furuuchi chemical), water and isopropanol, followed by drying with an air gun and finally plasma treatment. For the devices fabricated at Oxford, the patterned glass/FTO substrates (15 Ω sq^−^^1^, Latech Scientific Supply Pte. Ltd. or 10 Ω sq^−^^1^, AGC Inc.) were used. The PEDOT:PSS hole-transport layer was fabricated from an aqueous dispersion (without diluting), which was filtered through a 0.45-μm PVDF filter and then spin-coated on the FTO substrate using a spin programme of 10 s at 500 rpm followed by 30 s at 4,000 rpm. The films were then annealed in air at 140 °C for 20 min. After transferring to an Ar-filled (at Kyoto) or N_2_-filled (at Oxford) glovebox (H_2_O, O_2_ < 0.1 ppm), the substrates were degassed at 140 °C for 30 min. The perovskite layer was fabricated on PEDOT:PSS following the above-mentioned procedure, whereas the concentration of the precursor solution was increased to 1.9 M for cells fabricated at Oxford, as per the tandem cells fabricated. The samples were moved under Ar or N_2_ to a vacuum deposition chamber, in which 20 nm of C_60_ (deposition rate 0.01 nm s^−^^1^) and 8 nm of BCP (deposition rate 0.01 nm s^−^^1^) were deposited by thermal evaporation. The top electrode was prepared by depositing 100 nm of silver (Ag) through a shadow mask. The deposition rate for Ag was first set as 0.005 nm s^−^^1^ to reach 5 nm, then raised to 0.01 nm s^−^^1^ to reach 20 nm and finally raised to 0.08 nm s^−^^1^ to reach the target thickness. The cells fabricated at Kyoto for the EQE measurements were encapsulated with the AFTINNOVA-EF FD20 film (Ajinomoto Fine-Techno Co., Inc.)-pasted glass by heating at 65 °C for 5 min. The deposition rate for Cr and Au was 0.005 and 0.01 nm s^−^^1^, respectively. For Cr-containing cells, we note that cleaning the Cr bar to remove the oxidized species before each deposition is critical for the success of device fabrication.

### Tandems

When optimizing all perovskite tandem cells, the choice and optimization of the recombination layer between the subcells is critical. It should be highly transparent, enable maximum forward transmission and minimum reflection of sub-bandgap light transmitted through the previous cell, be suitably conductive to enable low loss recombination of electrons and holes from the adjacent subcells and be robust to the processing of subsequent cells, while protecting adjacent layers. Typically, for the high-efficiency two-junction tandem cells, a recombination layer stack comprises tin-oxide (SnO_*x*_) deposited from ALD and a very thin layer of metallic gold clusters (nominal 1 nm), followed by PEDOT:PSS. This works effectively between a WBG Pb-based and NBG Sn–Pb perovskite cells. However, there is substantial visible and near-infrared parasitic absorbance in both the gold clusters and the PEDOT:PSS and, if the second (or third) cell is a Pb-based cell, which will be the case for a triple junction or quadruple junction, then PEDOT:PSS is not the hole-transport material of choice. Here we have investigated a range of options for the recombination layer and find that a simple double layer of ALD-grown SnO_*x*_ followed by a thin (about 10 nm) layer of sputter-coated indium zinc oxide acts as a highly efficient, ‘universal recombination layer’. We also note that the diamine-based and diammonium-based post-treatment is very effective and serves as ‘universal post-treatment’ in improving the performance of the seven different perovskite absorbers applied in this study, which also verifies our previous studies^9,20,27,38,54,56,57^. Note that, the PEDOT:PSS layer deposited for the tandem cell fabrication was spin-coated with a diluted PEDOT:PSS dispersion (with two times the volume using IPA). The films were then annealed under 105 °C in the air for about 10 min, and another 10 min at 105 °C inside the glovebox before the deposition of narrow bandgap Sn–Pb perovskite films.

### Double junction

For all-perovskite double-junction solar cells, the perovskite absorbers were prepared as previously described. Between WBG and NBG perovskite subcells, after depositing PCBM, PEI solution (0.025 wt% diluted in IPA) was spin-coated at 4,000 rpm for 30 s (2,000 rpm s^−^^1^ acceleration) without any post-processing. 20 nm of ALD-grown SnO_*x*_ was then deposited on top and followed by sputtering of 10 nm IZO. Shadow masks were used to ensure that only active areas were covered with IZO to avoid shunt losses. Instead of BCP, 20 nm of ALD-grown SnO_*x*_ was deposited on top of NBG perovskite and C_60_.

For the double-junction cells fabricated at HUST, the NBG and WBG subabsorbers were fabricated with the recipe described above. For the interconnecting layer, C_60_ (18 nm), ALD-grown SnO_*x*_ (about 30 nm) and Au (about 0.8 nm) were deposited sequentially under vacuum. The layers above the NBG subabsorber were composed of C_60_ (25 nm), BCP (5 nm) and Ag (100 nm).

### Triple junction

For all-perovskite triple-junction solar cells, the perovskite absorbers were prepared as previously described. Between WBG and MBG perovskite subcells, after depositing PCBM, PEI solution (0.025 wt% diluted in IPA) was spin-coated at 4,000 rpm for 30 s (2,000 rpm s^−^^1^ acceleration) without post-processing. 20 nm of ALD-grown SnO_*x*_ was then deposited on top and followed by sputtering of 10 nm IZO. Between MBG and NBG perovskite subcells, 20 nm of ALD-grown SnO_*x*_ was deposited on C_60_ and followed by sputtering of 10 nm of IZO before spin-coating PEDOT:PSS in the air. Shadow masks were used to ensure that only active areas were covered with IZO to avoid shunt losses. Instead of BCP, 20 nm of ALD-grown SnO_*x*_ was deposited on top of NBG perovskite and C_60_.

### Quadruple junction

For all-perovskite quadruple-junction solar cells, the perovskite absorbers were prepared as previously described. Between WBG (2.26 eV) and WBG (1.80 eV) perovskite subcells, after depositing PCBM, PEI solution (0.025 wt% diluted in IPA) was spin-coated at 4,000 rpm for 30 s (2,000 rpm s^−^^1^ acceleration) without post-processing. 20 nm of ALD-grown SnO_*x*_ was then deposited on top and followed by sputtering of 10 nm IZO. Between WBG (1.80 eV) and MBG perovskite subcells, after depositing PCBM, PEI solution (0.025 wt% diluted in IPA) was spin-coated at 4,000 rpm for 30 s (2,000 rpm s^−^^1^ acceleration) without any post-processing. 20 nm of ALD-grown SnO_*x*_ was then deposited on top and followed by sputtering of 10 nm IZO. Between MBG and NBG perovskite subcells, 20 nm of ALD-grown SnO_*x*_ was deposited on C_60_ and followed by sputtering of 10 nm of IZO before spin-coating PEDOT:PSS in the air. Shadow masks were used to ensure that only active areas were covered with IZO to avoid shunt losses. Instead of BCP, 20 nm of ALD-grown SnO_*x*_ was deposited on top of NBG perovskite and C_60_.

In Fig. 4b, the thickness of the NBG absorber in the single-junction cells is about 930 nm. The thickness of the WBG and NBG subabsorbers in the double-junction cells is about 350 and 970 nm, respectively. The thickness of the WBG, MBG and NBG subabsorbers in the triple-junction cells is about 290, 550 and 940 nm, respectively. The thickness of the WBG (2.26 eV), WBG (1.80 eV), MBG and NBG subabsorbers in the quadruple-junction cells is about 250, 370, 740 and 900 nm, respectively.

All types of cell were encapsulated with a cover glass and ultraviolet (UV)-activated adhesive (Eversolar AB-341, Everlight Chemical Industrial Co.), which was cured under a UV LED lamp (peak emission at 365 nm) for 3 min inside an N_2_-filled glovebox (H_2_O, O_2_ < 0.1 ppm) before any related characterization under ambient conditions. Before the glass encapsulation, a 300-nm on-site encapsulation SiO_*x*_ layer^58^ was deposited on the top of the devices fabricated with the Cr (2.5 nm)/Au (90 nm) top electrode that was subjected to the 85 °C ageing tests.

### Characterizations

PLQE of samples was determined according to the method in ref. ^59^. Samples were placed inside an integrating sphere and excited from the substrate side with a 657-nm continuous-wave laser excitation source (Thorlabs) at 70.9 mW cm^−^^2^ (equivalent to 1 sun for a 1.26-eV bandgap) with a large spot size of 0.15 cm^2^. The resulting PL signal was collected using a fibre bundle (Ocean Optics QR600 7 SR125BX) coupled with a spectrometer (QE Pro, Ocean Optics). Three different spots were measured on each substrate. For intensity-dependent PLQE measurements, the same measurements were collected on one spot over a wide range of fluences from about 0.1 to 150 mW cm^−^^2^.

We applied a stray light correction to recorded PL spectra by subtracting the spectrum of the excitation laser (attenuated to the correct intensity according to the fraction of laser absorbed by the sample). PL emission spectra with a signal-to-noise ratio below 2 were not used. Furthermore, the detector used was not able to effectively detect PL above 1,050 nm, which means that, for the 1.26-eV bandgap samples used here, we were only able to map slightly more than half of the PL emission peak. To correct this, we fit the PL measurements to a pseudo-Voigt function and used the resulting peaks to calculate PLQE (fits were only used if *R*^2^ > 0.8 was achieved).

QFLS was calculated according to the following equation^60^:

$${\rm{QFLS}}={{\rm{QFLS}}}_{{\rm{rad}}}+{k}_{{\rm{B}}}T{\rm{ln}}({\rm{PLQE}})$$

in which *k*_B_ is the Boltzmann constant and *T* is the absolute temperature.

In the SRH model, the change in the density of free electrons, free holes and trapped electrons owing to an electron trap can be described by equations (1)–(3). Here *β*_n_ and *β*_p_ represent the electron and hole capture coefficients, respectively, *N*_t_ the trap density, *n*_t_ the density of trapped carriers, *N*_C_ and *N*_V_ the density of states at the conduction and valence bands, respectively, *E*_CB_ and *E*_VB_ the energies of the conduction and valence bands, respectively, and *E*_T_ the energy of the trap state transition. The first term in each equation describes the trapping rate and the second term describes the detrapping rate.

1 $${R}_{\text{n-trapping}}=-{\beta }_{{\rm{n}}}n({N}_{{\rm{t}}}-{n}_{{\rm{t}}})+{\beta }_{{\rm{n}}}\left({N}_{{\rm{C}}}{{\rm{e}}}^{\frac{{E}_{{\rm{T}}}-{E}_{{\rm{CB}}}}{kT}}\right){n}_{{\rm{t}}}$$

2 $${R}_{\text{p-trapping}}=-{\beta }_{{\rm{p}}}p{n}_{{\rm{t}}}+{\beta }_{{\rm{p}}}\left({N}_{{\rm{V}}}{{\rm{e}}}^{\frac{{E}_{{\rm{VB}}}-{E}_{{\rm{T}}}}{kT}}\right)({N}_{{\rm{t}}}-{n}_{{\rm{t}}})$$

3 $$\frac{{\rm{d}}{n}_{{\rm{t}}}}{{\rm{d}}t}={R}_{\text{p-trapping}}-{R}_{\text{n-trapping}}$$

We assume that the doping density *p*_0_ in this material (approximately 10^14^ cm^−3^ when photoexcited charge densities are expected to be around 10^14^–10^15^ cm^−^^3^) is sufficiently low such that n = p = Δ*n* is valid. Further assumptions leading to the model used for fitting were as in ref. ^40^ but are briefly detailed below.

At a steady state, the hole and electron trapping rates are equal. The equation resulting from this can be rearranged to solve for the trap occupancy *n*_t_, which is then used to determine the defect-mediated recombination rate given in equation (4). Here the lifetimes are used to denote ${\tau }_{{\rm{p}}}=\frac{1}{{\beta }_{{\rm{p}}}{N}_{{\rm{T}}}}$ and ${\tau }_{{\rm{n}}}=\frac{1}{{\beta }_{{\rm{n}}}{N}_{{\rm{T}}}}$. As detrapping is only expected to be substantial in one direction (to the VB or to the CB), we can simplify equation (4) to equation (5) for an electron trap.

4 $${R}_{{\rm{SRH}}}=\frac{{n}^{2}}{\left(n+{N}_{{\rm{C}}}{{\rm{e}}}^{\frac{{E}_{{\rm{T}}}-{E}_{{\rm{CB}}}}{kT}}\right){\tau }_{{\rm{n}}}+\left(n+{N}_{{\rm{C}}}{{\rm{e}}}^{\frac{{E}_{{\rm{T}}}-{E}_{{\rm{CB}}}}{kT}}\right){\tau }_{{\rm{p}}}}$$

5 $${R}_{{\rm{SRH}}}=\frac{{n}^{2}}{\left(n+{N}_{{\rm{C}}}{{\rm{e}}}^{\frac{{E}_{{\rm{T}}}-{E}_{{\rm{CB}}}}{kT}}\right){\tau }_{{\rm{n}}}+n{\tau }_{{\rm{p}}}}$$

For a deep (mid-gap) trap, we make further simplifications by considering the detrapping rate to be negligible. For a deep trap, the recombination rate thus simplifies to:

6 $${R}_{{\rm{SRH}}}=\frac{n}{{\tau }_{{\rm{n}}}+{\tau }_{{\rm{p}}}}$$

The total recombination rate resulting from one shallow electron trap and one deep trap can hence be described by equation (7). Assuming equal rates of electron and hole trapping for both the shallow and the deep traps leads to equation (8).

7 $${R}_{{\rm{SRH}}}=\frac{n}{{\tau }_{{\rm{deep,n}}}+{\tau }_{{\rm{deep,p}}}}+\frac{{n}^{2}}{\left(n+{N}_{{\rm{C}}}{{\rm{e}}}^{\frac{{E}_{{\rm{T}}}-{E}_{{\rm{CB}}}}{kT}}\right){\tau }_{{\rm{shallow,n}}}+n{\tau }_{{\rm{shallow,p}}}}$$

8 $${R}_{{\rm{SRH}}}=\frac{n}{{\tau }_{{\rm{deep}}}}+\frac{{n}^{2}}{{\tau }_{{\rm{shallow}}}\left(\frac{1}{2}{N}_{{\rm{C}}}{{\rm{e}}}^{\frac{{E}_{{\rm{T}}}-{E}_{{\rm{CB}}}}{kT}}+n\right)}$$

Computational studies have indicated that the expected trap depths in Sn–Pb perovskites range from 0.05 to 0.35 eV (relatively shallow compared with other perovskite compositions)^41^, but precise trap depths and associated electron and hole capture coefficients have not been determined. We hence use a model that contains a trap to represent both ‘extremes’—one relatively deep electron trap (0.35 eV) and one very shallow hole trap (0.05 eV).

Intensity-dependent PLQE fitting was performed by modelling the recombination as below, then solving for the steady-state carrier density at each excitation density. The background hole density in the lead–tin perovskite used here is expected to lie around 10^14^ cm^−3^, hence we expect to be in the high injection regime for all fluences used here.

9 $$\frac{{\rm{d}}n}{{\rm{d}}t}=0=G-{k}_{{\rm{trap,deep}}}n-{k}_{{\rm{trap,shallow}}}\frac{{n}^{2}}{2n+{n}_{1}}-{k}_{{\rm{rad,ext}}}{n}^{2}-{k}_{{\rm{aug}}}{n}^{3}$$

Here *G* is the generation rate of charge carriers obtained from the laser intensity, spot size and perovskite thickness, *k*_rad,ext_ is the external radiative bimolecular recombination rate and *k*_aug_ is the Auger recombination rate. Trap-assisted recombination is modelled through one deep trap, from which carriers cannot be emitted, and one shallow trap, from which carriers can be emitted before recombining. *k*_trap_ is the trapping rate into the trap and *n*_1_ is related to the energetic depth of the trap (Δ*E*_t_) through

10 $${n}_{1}={N}_{{\rm{C}}}{{\rm{e}}}^{-\Delta {E}_{{\rm{t}}}/{k}_{{\rm{B}}}T}$$

*N*_C_ is the density of states in the conduction band and is assumed to be 10^18^ cm^−^^3^ here. The equilibrium carrier concentration under the continuous illumination obtained by solving equation (9), *n*_eq_, is then used to calculate the PLQE according to

11 $${{\rm{PLQE}}}_{{\rm{int}}}=\frac{{k}_{{\rm{rad}}}{{n}_{{\rm{eq}}}}^{2}}{{k}_{{\rm{rad}}}{{n}_{{\rm{eq}}}}^{2}+{k}_{{\rm{trap,deep}}}{{n}_{{\rm{eq}}}}^{2}+{k}_{{\rm{trap,shallow}}}\frac{{{n}_{{\rm{eq}}}}^{2}}{2{n}_{{\rm{eq}}}+{n}_{1}}+{k}_{{\rm{aug}}}{{n}_{{\rm{eq}}}}^{2}}$$

In accordance with the previous report^61^, the measured external PLQE is then described by

12 $${{\rm{PLQE}}}_{{\rm{ext}}}=\frac{{P}_{{\rm{esc}}}{{\rm{PLQE}}}_{{\rm{int}}}}{(1-{{\rm{PLQE}}}_{{\rm{int}}})+({P}_{{\rm{esc}}}{{\rm{PLQE}}}_{{\rm{int}}})}$$

in which *P*_esc_ is the escape probability of a photon generated within the perovskite material. We solve these equations iteratively for each generation rate to extract the parameters from the data. Fitting was performed using the Levenberg–Marquardt algorithm in limit and uncertainties were calculated by inverting the second derivative matrix of fit quality for each variable parameter.

TRPL measurements were collected by exciting samples from the substrate side with a 635-nm laser head (LDH-635, PicoQuant GmbH) pulsed at a frequency of 10 kHz with a fluence of 4.9 nJ cm^−^^2^ pulse^−1^ and a spot size of 2.43 × 10^−3^ cm^2^. The TRPL signal was collected from the same side with a time-correlated single-photon-counting set-up (FluoTime 300, PicoQuant GmbH using a TimeHarp 260 for photon counting). The emission was individually attenuated to keep the detector pile-up rate under 5%.

The resulting data were processed by subtracting a background determined by the mean signal intensity during the 40 ns before the excitation pulse and normalizing. The tails of the resulting decays were fitted with a stretched exponential decay between 300 ns and the time at which the signal-to-noise ratio dropped below 2.

Differential lifetime over time was plotted by first fitting the decays using a superposition of five exponential decays to smooth the data. The differential lifetime was then calculated according to the following equation^43^:

$${\tau }_{{\rm{diff}}}={\left(-\frac{1}{2}\frac{{\rm{d}}{\rm{ln}}({\rm{PL}})}{{\rm{d}}t}\right)}^{-1}$$

The QFLS images were obtained using the same set-up as our previous report^47^, which is also demonstrated with the scheme below. The sample was optically excited with a 450-nm LED and electronically contacted by a sourcemeter.

The intensity of the LED that corresponds to an equivalent 1-sun illumination was determined by measuring the current on a fully fabricated device at a short circuit and varying the intensity until this current matched that measured on our calibrated solar simulator. The PL images were captured on an Andor Zyla CMOS image sensor. A long-pass filter was used to stop the excitation light.

The PLQE was estimated by imaging the excitation light incident on a white reference plate, placed on the focal plane of the camera, without a filter. We use this as a dividing factor for the PLQE, considering the wavelength-dependent response of the camera lenses and the image sensor itself. For each measurement, the illumination was held for 30 s before measurement, to allow for the PL to stabilize.

### Calculation of the *V*_OC_ loss based on EQE_EL_ measurements

EQE_EL_ of the triple-junction cells was measured on a home-built EQE_EL_ platform at room temperature, in which an Ocean Insight QE Pro Spectrometer and an integrating sphere coupled with fibre were used for the EL spectra collection and a Keithley 2400 was used to drive the devices. The system was calibrated by a standard light source (HL-3P-INT-CAL radiometrically calibrated tungsten halogen light source) for the absolute spectral response of the spectrometer. The EQE_EL_ values of all three junctions were calculated from the associated emission peaks.

$${\rm{EQE}}({\rm{EL}})=\frac{{N}_{{\rm{photon(V)}}}}{{N}_{{\rm{charges(V)}}}}\times 100 \% =\int \frac{{\Phi }_{\lambda }\lambda e}{hcJA}{\rm{d}}\lambda \times 100 \% $$

in which *N*_photo_ and *N*_charges_ are emitted photons and injected charge carriers, respectively; *Φ*_*λ*_ (µw nm^−^^1^) is spectral flux, *h* is the Plank constant (6.626 × 10^−^^34^ J s), *c* is the speed of light in vacuum (2.998 × 10^8^ m s^−^^1^), *J* (mA cm^−^^2^) is the current density and *e* is the elementary charge (1.602 × 10^−^^19^ C). The working area of the device is *A* (m^2^).

The *V*_OC_ loss values were calculated following the equation

$${\Delta V}_{{\rm{OC,nrad}}}=-\frac{{k}_{{\rm{B}}}T}{q}{\rm{ln}}({{\rm{EQE}}}_{{\rm{EL}}}({J}_{{\rm{SC}}}))$$

in which *q* is the elementary charge and *k*_B_ is the Boltzmann constant. EQE_EL_(*J*_SC_) is the EQE_EL_ (ratio of emitted photons to the injected charge carriers) of the solar cell at an injected current density that is the same as the *J*_SC_ of the triple-junction cells under the simulated AM 1.5 G illumination.

### TPC

Transient photoconductivity (TPC) was measured using a homebuilt set-up. Interdigitated gold electrodes spaced 300 and 500 μm apart were evaporated onto the sample on a glass substrate and used to bias the sample with a weak electric field of <0.01 V μm^−^^1^. The sample was illuminated from the substrate side by a 10-Hz pulsed laser at 650 nm with power densities <1 mW cm^−^^2^. Discrete neutral density filters were used to change the excitation intensity over an area of 0.25 cm^2^. The excitation pulse used during the TPC experiment had a pulse width of 3.7 ns and was used as an optical trigger for a digital oscilloscope (Tektronix DPO3054), which measures the voltage across a terminal resistor. More details about the technique can be found in our previous report^62^.

### Extracting mobility from TPC

A more detailed explanation has been reported in our previous report^62^. In short, the sum mobility (Σ*μ*) can be estimated from the photoconductivity through

$${\sigma }_{{\rm{photo}}}(t=0)=e\phi \Sigma \mu {N}_{0},$$

in which *e* is the elementary charge, *N*_0_ is the excitation charge carrier density and *Φ* is the fraction of free charge carriers. The last of these needs to be estimated to obtain Σ*μ*. This is done by taking both recombination during the laser pulse as well as exciton formation at higher excitation intensities into account. The corrected Σ*μ* is then largely independent of excitation intensity. We report the median mobilities of six intensities of two samples each for the control and PhA cases. We find a much lower in-plane mobility for the modified case.

SEM at Kyoto was performed with a Hitachi S8010 ultra-high-resolution scanning electron microscope (Hitachi High-Tech Corporation) under the accelerating voltage of 2 kV for the experiments (cross and top view of the perovskite films) conducted in Kyoto. The cross-section images of the devices were recorded by a FEI Quanta 3D FEG microscope at Oxford. A 10-kV electron beam with a spot size of 2 and a secondary electron detector were used. A typical working distance was about 11 mm and dwell time for single-pass image acquisition was 5 μs. Focusing and alignment were done away from imaged areas to minimize electron-beam-induced damage.

*J–V* curves for the devices fabricated at Kyoto were measured in an N_2_-filled glovebox (H_2_O, O_2_ < 0.1 ppm) with an OTENTO-SUN-P1G solar simulator (Bunkoukeiki Co., Ltd.). The light intensity of the illumination source was calibrated using a standard silicon photodiode. Each device was measured with a 10-mV voltage step and a 100-ms time step (that is, scan rate of 0.1 V s^−^^1^) using a Keithley 2400 sourcemeter. The active area of the device was defined by an optical mask, 0.0985 cm^2^ for the usual devices. Steady-state power output measurement was performed by holding the device at the voltage of the maximum power point, as determined by *J–V* characterization, and monitoring the current density over the period examined.

For the devices fabricated at Oxford, *J*–*V* and maximum power point measurements (in turbo mode with four cells measured in parallel) were measured using four Keithley 2400 series sourcemeters in the ambient environment under both simulated sunlight (AM 1.5 G irradiance generated by a Wavelabs SINUS-220 simulator) and in the dark (for the dark *J*–*V*). The active area of the solar cell was masked with a black-anodized metal aperture to either 0.25 or 1 cm^2^, within a light-tight holder. The *J*–*V* characteristics were taken from a reverse scan (that is, from forward bias to short circuit) followed by a forward scan (that is, from short circuit to forward bias) at a scan rate of 300 mV s^−^^1^. Subsequently, active MPPT measurements using a gradient descent algorithm were performed for the projected period to obtain the MPPT efficiency as the steady-state performance. The intensity of the solar simulator was set periodically such that the short-circuit current density from a WPVS reference cell (monocrystalline silicon solar cell, provided and certified by Fraunhofer ISE) matched its 1-sun certified value. The mismatch factor, estimated for the test cells, calibration cells and simulator, was estimated to be less than 1% and, hence, was not applied^63^.

EQE spectra for single-junction devices fabricated at Kyoto were measured with a Bunkoukeiki SMO-250III system equipped with a Bunkoukeiki SM-250 diffuse reflection unit (Bunkoukeiki Co., Ltd.). The incident light intensity was calibrated with a standard SiPD S1337-1010BQ silicon photodiode.

For the devices fabricated at Oxford, we measured the EQE using a homebuilt set-up. To determine the EQE, the photocurrent spectrum of the device under test was divided by that of a calibrated Si reference cell (Newport) of a known EQE. The light illumination first goes through a monochromator (Princeton Instruments SP2150) with a filter wheel (Princeton Instruments FA2448) and is followed by being chopped with an optical chopper (Thorlabs MC2000B) at 280 Hz and focused onto the measured cell with a smaller spot size than the solar cell area (as defined by the metallic top contact). The amplitude of the resulting AC signal was measured with a lock-in amplifier (Stanford Research Systems SR830) as the voltage drop across a 50-Ω resistor connected in series with the solar cell under measurement. For double-junction tandem devices, we measured the EQE response from the WBG subcell by biasing the NBG subcell to its open-circuit voltage conditions with an LED light at the wavelength of 850 nm. The response of the NBG subcell was recorded by biasing the WBG subcell to its open-circuit voltage conditions with an LED light at the wavelength of 455 nm. For triple-junction dells, we measured the EQE response from the WBG subcell by biasing the MBG and NBG subcells to their open-circuit voltage conditions with two LED lights at wavelengths of 740 and 850 nm. The response of the MBG subcell was recorded by biasing the WBG and NBG subcells to their open-circuit voltage conditions with two LED lights at wavelengths of 455 and 850 nm. The response of the NBG subcell was recorded by biasing the WBG and MBG subcells to their added open-circuit voltage conditions with two LED lights at wavelengths of 455 and 740 nm. For quadruple-junction dells, we measured the EQE response from the WBG1 (2.26 eV) subcell by biasing the WBG2, MBG and NBG subcells to their open-circuit voltage conditions with three LED lights at wavelengths of 585, 740 and 850 nm. The response of the WBG2 subcell was recorded by biasing the WBG1, MBG and NBG subcells to their open-circuit voltage conditions with three LED lights at wavelengths of 455, 740, and 850 nm. The response of the MBG subcell was recorded by biasing the WBG1, WBG2 and NBG subcells to their open-circuit voltage conditions with three LED lights at wavelengths of 455, 585 and 850 nm. The response of the NBG subcell was recorded by biasing the WBG1, WBG2 and MBG subcells to their added open-circuit voltage conditions with three LED lights at wavelengths of 455, 585 and 740 nm. LED light sources were purchased from Mightex. The measurements were conducted under ambient conditions.

To determine the equivalent short-circuit current density under 1-sun irradiance from the EQE measurements, the overlap integral of the AM 1.5 photon flux (*φ*_AM1.5_) spectrum with the EQE was calculated. Explicitly, this is given by

$${J}_{{\rm{SC}}}=q{\int }_{0}^{\infty }{\rm{d}}\lambda {\rm{EQE}}(\lambda ){\varphi }_{{\rm{AM1.5}}}(\lambda )$$

in which *q* is the elementary charge and *λ* is the wavelength.

Ultraviolet photoelectron spectroscopy (UPS) was performed with a photoelectron spectroscopy system (PHI 5000 VersaProbe II, ULVAC-PHI, Inc.) with He I excitation (21.22 eV). A −5.0 V bias was applied to the samples. The chamber base pressure was approximately 1 × 10^−6^ Pa. Samples were transferred from the glovebox to the UPS chamber without air exposure.

X-ray photoelectron spectroscopy (XPS) was performed with a photoelectron spectroscopy system (PHI 5000 Versa Probe II, ULVAC-PHI, Inc.). Monochromated Al Kα (1,486.6 eV) radiation with an operating power of 50 W (15 kV voltage) was used in all of the XPS measurements. The diameter of the analysed area was 200 μm. The take-off angle was 45° to the substrate. For the measurement of each atomic element, pass energy of 46.95 eV and 0.050-eV energy steps were used. Four different positions of the samples were analysed and obtained spectra were averaged to improve the signal-to-noise ratio. No sample charging was observed during all of the measurements.

XRD and 2D GIWAXS measurements were performed on a Rigaku SmartLab equipped with a goniometer-mounted 2D hybrid pixel array detector (HyPix-3000) with a rotating Cu Kα source (*λ* = 1.5406 Å). Perovskite films were deposited on the top of PEDOT:PSS with glass/FTO as substrates to mimic the growth conditions in full devices and covered with a thin film of spin-coated PMMA to prevent direct exposure to air. For GIWAXS, samples were mounted on a 2D XRD attachment head with a knife-edge to prevent air scatter, positioned directly behind a pinhole to reduce sample footprint broadening, with a beam stop for the direct beam. The sample-to-detector distance was 65 mm, the incidence angle was set to 0.4°, the detector 2*θ* angle was 13.5° and the total collection time was 10 min. Detector images were then resampled into *Q*-space using scripts based on the pyFAI and pygix libraries^64^.

#### NMR spectra

^1^H NMR spectra were recorded on a Bruker Avance 400 spectrometer (400 MHz). ^119^Sn and ^207^Pb NMR spectra were recorded on a Bruker Avance III 500 NMR spectrometer (500 MHz). The NMR chemical shifts are reported in ppm relative to the residual protons of DMSO-*d*_6_ (*δ* = 2.54 ppm) and –HC=O proton of DMF-*d*_*7*_ at 7.95 ppm. The metal NMR set-up was calibrated with measured shielding constant of tetramethyl metal reference compounds. The samples were prepared by dissolving the subjected content(s) into 0.6 ml DMSO-*d*_6_ and DMF-*d*_7_ mixed solvent with a concentration of 1.85 M. After filtration through a 0.20-μm PTFE filter, the solutions were injected into NMR tubes for testing.

DLS was conducted with an ELSZ-2000 particle size analyser (Otsuka Electronics Co., Ltd.). The perovskite precursor solution preparation was conducted in an Ar-filled glovebox (H_2_O, O_2_ < 0.1 ppm). The 1.85 M Cs_0.1_FA_0.6_MA_0.3_Sn_0.5_Pb_0.5_I_3_ perovskite precursor solution was prepared as a foundation reference. To prepare the PhA, PEA, PPA and PEAPPA samples, 3 mol% of the additives, that is, PhA, PEA, PPA and PEA, together with PPA, with respect to the total amount of SnI_2_ and PbI_2_, were respectively added to the foundation precursor materials to form these modified 1.85 M solutions. As stated in the perovskite preparation section, the precursor solution was stirred at 45 °C for 30 min and filtered through a 0.20-µm PTFE filter before being subject to the DLS tests. 2.0 ml perovskite precursor solution for each condition was loaded in a clean quartz cuvette for measurement (Ar-sealed). The DLS measurement was conducted at 0, 10, 30, 60, 90, 120 and 240 min after the sample preparation for each condition. The refractive indices and viscosity of the solvent were 1.4405 and 1.1005, respectively, based on the DMF and DMSO with a volume ratio of 3:1. CONTIN analysis mode was applied.

ToF-SIMS measurements were carried out using a ToF-SIMS 5 (IONTOF GmbH) operated in the high-lateral-resolution mode (burst alignment mode). A 60-keV Bi_3_^2+^ primary ion beam with a pulse width of 3 ns was used for data acquisition. Primary ion dose density was maintained at less than approximately 5 × 10^11^ ions cm^−^^2^ in each measurement cycle to prevent sample damage from the irradiation of the primary ion beam. For sample etching, a 10-keV Ar_1200_^+^ gas cluster ion beam with a centre size of approximately 1,200 atoms was used as a sputtering ion beam. Depth profiles of treated ingredients (or ions) were reconstructed from the raw data after data acquisition. Also, mass spectra and depth profiles with high mass resolution were acquired from an area of 200 µm × 200 µm by using high-mass-resolution mode (bunching mode). A low-energy electron flood gun was used for charge compensation. The films were loaded under an N_2_ atmosphere.

For 85 °C ageing, encapsulated devices fabricated with the Cr (2.5 nm)/Au (90 nm) top electrode were subjected to an oven with the temperature set at 85 °C for thermal ageing. For the dark ageing, the ageing oven was put inside a glovebox (O_2_ < 10 ppm, H_2_O < 0.1 ppm). For the light ageing, the ageing chamber for storing the encapsulated samples was air-cooled with the temperature controlled at 85 °C (measured using a black standard temperature control unit positioned next to the test cells). During the ageing period, the relative humidity in the laboratory (at about 20 °C) was monitored at about 45%.

#### MPPT test

The operating stability tests for MPPT were carried out under simulated AM 1.5 G illumination (Class ABA Newport solar simulator (LSH-7320)) with 1-sun intensity using a homebuilt LabVIEW-based MPPT script and a Keithley 2401 sourcemeter to track the cell performance in ambient conditions (temperature approximately 25 °C, RH 30–50%). No UV filter was applied during operation but we do note that this simulator generates very little UV light. The spectrum of the solar simulator can be accessed at: https://web.archive.org/web/20240502204722/https://www.newport.com/mam/celum/celum_assets/np/resources/DS-092001_MiniSol_LED_Solar_Simulator_Datasheet.pdf?3.

Highly sensitive EQE measurements used the light from an Osram 64655 HLX 250 W tungsten halogen lamp mechanically chopped at 333 Hz passing appropriate sorting filters (OD > 5) and dispersed using an Oriel Cornerstone 260 monochromator. The cell was kept in an electrically insulated nitrogen-filled container. The response was recorded using a Stanford Research SR570 preamplifier and a Stanford Research SR830 lock-in amplifier. Calibration was performed using reference Si and InGaAs detectors. The measured highly sensitive EQE spectra were scaled to normal EQE data.

Optical simulations and optimizations were performed using a custom-made program^65^, written in Python, based on the ‘tmm’ transfer-matrix modelling Python module^66^. The optical constants for each of the layers in the device stacks were obtained from our previous studies^20,67^.

### Computational methods

As learnt from the previous single-crystal results^68^, the Sn(ii) and Pb(ii) in perovskite prepared with FA_0.5_MA_0.5_Sn_0.5_Pb_0.5_I_3_-subjected composition show equal occupation at the B-site of the ABX_3_ structure. This indicates that the Sn(ii) and Pb(ii) in the Sn–Pb perovskites are more likely to assemble their octahedral structures in a way that all of the Sn(ii) are surrounded by Pb(ii), and vice versa. We constructed the Cs_0.125_FA_0.625_MA_0.25_Sn_0.5_Pb_0.5_I_3_ A–I-terminated (001) surface (about 25 × 25 Å^2^) with a vacuum of 20 Å to separate neighbouring surfaces in the *z* direction. DFT calculations were performed by the Perdew–Burke–Ernzerhof generalized gradient approximation and the projector augmented wave as implemented in the Vienna Ab initio Simulation Package (VASP)^69–73^. The DFT-D3 method was used for the van der Waals correction^74^. Dipole corrections were included for the slab calculations. The plane-wave cut-off energy of 400 eV was used. The Brillouin zone was sampled using a single gamma point. The energy and force convergence criteria were set to 10^−5^ eV and 0.03 eV Å^−1^, respectively. The binding energies (*E*_b_) of adsorbate molecules with the perovskite surface were calculated as *E*_mol/pvsk_ − *E*_pvsk_ − *E*_mol_, in which *E*_mol/pvsk_, *E*_pvsk_ and *E*_mol_ are the total energies of the adsorption system, the perovskite system and adsorbate molecules, respectively.

Calculations on the configuration of MAI, FAI and SnI_2_ with amino acid salt molecules are performed by the ωB97X-D functional^75^ combined with the 6-311G(2d,p)^76–78^ (for C, N, O, and H atoms) and LANL2DZ^79^ (for Sn and I atoms) basis sets. Optimized structures were confirmed as local minima through vibrational frequency analysis. Interaction energies were calculated using the following equation: Δ*E*_int_ = *E*(MAI/FAI/SnI_2_ + amino acid) − (*E*(MA/FA/Sn) + *E*(I/I/2I) + *E*(amino acid)), considering the ionic nature of the species. The above calculations were carried out using the Gaussian 16 suite of programs version A.03 (ref. ^80^). The electrostatic potentials were calculated at the level of ωB97X-D/6-311G(2d,p) (LANL2DZ for Sn and I) and corresponding values were obtained by Multiwfn^81^.

### Reporting summary

Further information on research design is available in the Nature Portfolio Reporting Summary linked to this article.

## Online content

Any methods, additional references, Nature Portfolio reporting summaries, source data, extended data, supplementary information, acknowledgements, peer review information; details of author contributions and competing interests; and statements of data and code availability are available at 10.1038/s41586-024-08546-y.

## Supplementary information

Supplementary Information Supplementary Information, including Supplementary Figs. 1–61, Supplementary Tables 1–3 and Supplementary References.

Reporting Summary

## Data Availability

The data supporting the findings of this study are available from the corresponding author on request.
