# Supplementary material for: Steering perovskite precursor solutions for multijunction photovoltaics
Source: Nature. 2024 Dec 23;639(8053):93–101. doi: 10.1038/s41586-024-08546-y (PMC11882461; doi:10.1038/s41586-024-08546-y)
Supplement: Supplementary file 1 — Supplementary Information, including Supplementary Figs. 1–61, Supplementary Tables 1–3 and Supplementary References. [file 41586_2024_8546_MOESM1_ESM.pdf]

nature portfolio

https://doi.org/10.1038/s41586-024-08546-y

# Supplementary information

# Steering perovskite precursor solutions for multijunction photovoltaics

In the format provided by the authors and unedited

**Supporting information**

# Steering perovskite precursor solutions for multijunction photovoltaics

Shuaifeng Hu,*,†,a,b Junke Wang,†,a Pei Zhao,a,c Jorge Pascual,b,d Jianan Wang,e,f Florine Rombach,a Akash Dasgupta,a Wentao Liu,b Minh Anh Truong,b He Zhu,e,f Manuel Kober-Czerny,a James N. Drysdale,a Joel A. Smith,a Zhongcheng Yuan,a Guus J. W. Aalbers,g Nick R. M. Schipper,g Jin Yao,h Kyohei Nakano,i Silver-Hamill Turren-Cruz,b,j André Dallmann,k M. Greyson Christoforo,a James M. Ball,a David P. McMeekin,a Karl-Augustin Zaininger,a Zonghao Liu,e,f Nakita K. Noel,a Keisuke Tajima,i Wei Chen,e,f Masahiro Ehara,c René A. J. Janssen,g,l Atsushi Wakamiya,*,b and Henry J. Snaith*,a

[a]Clarendon Laboratory, Department of Physics, University of Oxford, Oxford OX1 3PU, U.K.

[b]Institute for Chemical Research, Kyoto University, Gokasho, Uji, Kyoto 611-0011, Japan.

[c]Research Center for Computational Science, Institute for Molecular Science, Okazaki, 444-8585, Japan.

[d]Polymat, University of the Basque Country UPV/EHU, 20018 Donostia-San Sebastian, Spain.

[e]Wuhan National Laboratory for Optoelectronics, Huazhong University of Science and Technology (HUST), Luoyu Road 1037, Wuhan, 430074, China.

[f]Optics Valley Laboratory, Hubei 430074, China.

[g]Molecular Materials and Nanosystems and Institute for Complex Molecular Systems, Eindhoven University of Technology, P.O. Box 513, 5600 MB, Eindhoven, The Netherlands.

[h]National Thin Film Cluster Facility for Advanced Functional Materials, Department of Physics, University of Oxford, Oxford OX1 3PU, U.K.

[i]RIKEN Center for Emergent Matter Science (CEMS), Wako, Saitama 351-0198, Japan.

[j]Instituto de Ciencia de los Materiales (ICMUV), Universitat de Valencia, 46980 Paterna, Spain.

[k]Humboldt Universität zu Berlin, Institut für Chemie, AG NMR, Germany.

[l]Dutch Institute for Fundamental Energy Research, Eindhoven, The Netherlands.

[†]These authors contributed equally.

**Corresponding authors:** shuaifeng.hu@physics.ox.ac.uk, wakamiya@scl.kyoto-u.ac.jp, henry.snaith@physics.ox.ac.uk

**Keywords:** solar cell • perovskite • tandem • solution chemistry • crystallisation • defect passivation • amino acid

**Liquid-state NMR**

We first investigate additive-specific differences in the solution chemistry of the perovskite precursor solution components. The solutions are prepared with a narrow bandgap (~1.26 eV) mixed Sn–Pb perovskite composition of $Cs_{0.1}FA_{0.6}MA_{0.3}Pb_{0.5}Sn_{0.5}I_3$ (MA = methylammonium, FA = formamidinium), as reported in our previous work[1-3], with DMF and DMSO in a volume ratio of 3:1 as the solvents at a concentration of 1.85 M. In addition, 10 mol% $SnF_2$, with respect to $SnI_2$, and 2 mol% $NH_4SCN$, with respect to the total amount of the B-site precursor ($SnI_2$ + $PbI_2$), are added to the precursor solutions serving as the "baseline additives"[1-3].

The nature of the iodoplumbate and iodostannate species that are present within these perovskite precursor solutions will impact the crystallization process and evolution into thin films. Different solution characterization techniques can probe how additives interact with precursor materials and affect metal-centred complexes[4]. Solution nuclear magnetic resonance (NMR) spectroscopy, as a non-destructive technique, can be used to characterize the chemical profile of ammonium, carboxylic acid, and amino acid functional groups by probing $^{1}H$ nuclei. We can further analyse the electromagnetic environment of tin and lead with measurements on $^{119}Sn$ and $^{207}Pb$ nuclei, particularly useful for perovskites, and offering unique insights into the specific interactions that these components and functional groups establish.

**Supplementary Fig. S1** shows PhA in combination with $NH_4SCN$ additive. We can see that the coupling of ammonium cation $NH_4^+$ protons with $^{14}N$ splits the peak at 7.8 ppm into the common triplet observed for this molecule. Apart from this, we found no significant shift of any type, concluding that there is no major interaction between the additives, also possibly due to the low concentrations in which they are used. **Supplementary Fig. S2** shows PhA in combination with $SnF_2$ additive. We can see that all signals broaden, such as ammonium ones that slightly shift. Meanwhile, the signal from the carboxylic acid group proton disappears. This is probably caused by fluoride anions promoting proton exchange with protic functional groups ammonium and carboxylic acid. In addition, the tendency of the carboxylic group to coordinate $Sn^{2+}$ cations may also promote the deprotonation of this functionality, making it not detectable in the NMR spectra.

The iodide content does not determine these variations in the characteristics of these protons. All individual components are mixed with PhA in the relative proportions used for perovskite thin film deposition (see Experimental Details section). If iodide concentration had a direct effect on the electronic environment of PhA protons, we would have observed different chemical shift values for MAI- and FAI-containing solutions (the latter is more concentrated). Therefore, the availability of these ions is not influencing the extent of the shift, as the coordinative bonds of PhA appear to saturate already at very low concentrations. In the same way, we would expect a slight contribution from the presence of chloride when adding different salts. However, as in the case of **Fig. 1b**, we obtain very different behaviours separately for Sn and Pb and within Sn when using different additives containing the same amount of chloride. Therefore, though some influence from chloride

should not be fully neglected, the role of the additives is evident, and the mechanism proposed according to our observations is independent of this potential effect from chloride.

For solutions containing the full perovskite composition (**Supplementary Fig. S5**), the carboxylic acid signal widened and shifted upfield over 2 ppm, an effect that the acid-only component PPA also presented, with a 0.7 ppm shift. PhA and PEA present strong shifts of their ammonium protons of 0.78 ppm and 0.98 ppm, respectively, when in combination with perovskite components.

Together with the discussion in the main text, the NMR observations highlight the higher reactivity of Sn-based precursors and their leading role in determining solution characteristics compared to the Pb-based analogues in the same system. Thus, understanding and controlling the chemical environment of Sn-based species will be particularly critical for altering the properties of Sn-containing perovskites.

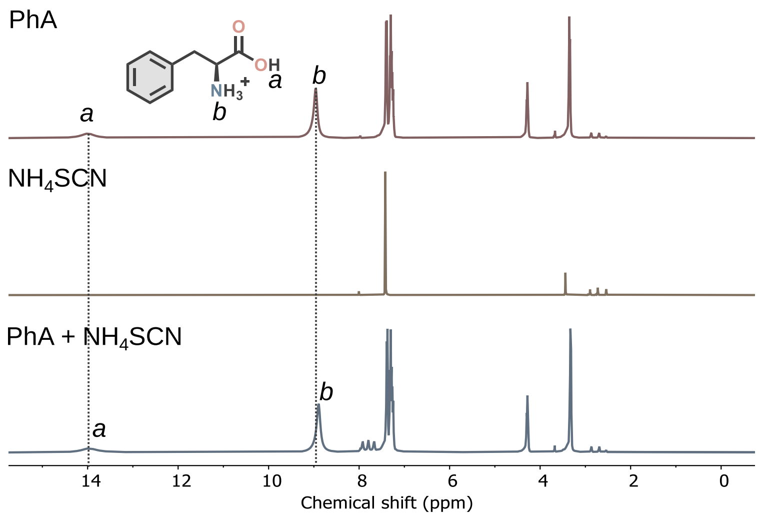


**Supplementary Fig. S1:** $^1$H NMR spectra of PhA, $NH_4SCN$, and PhA + $NH_4SCN$ dissolved in DMSO-$d_6$ and DMF-$d_7$ (v:v, 1:3) mixed solvent.

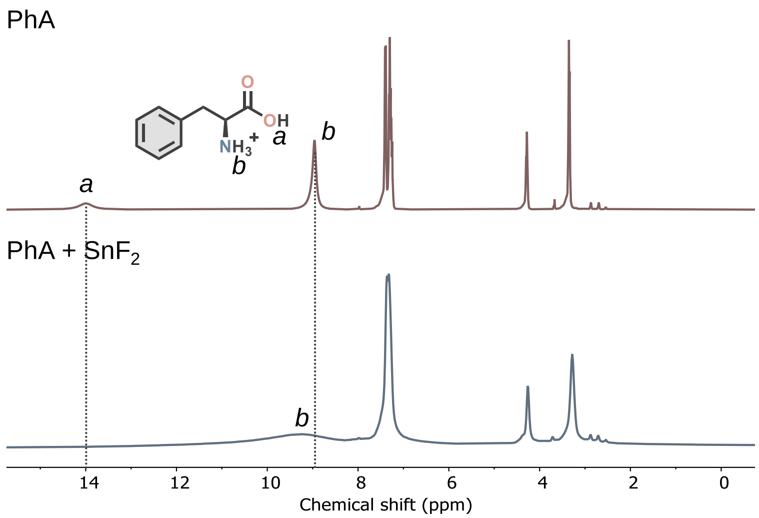


**Supplementary Fig. S2:** $^1$H NMR spectra of PhA and PhA + $SnF_2$ dissolved in DMSO-$d_6$ and DMF-$d_7$ (v:v, 1:3) mixed solvent.

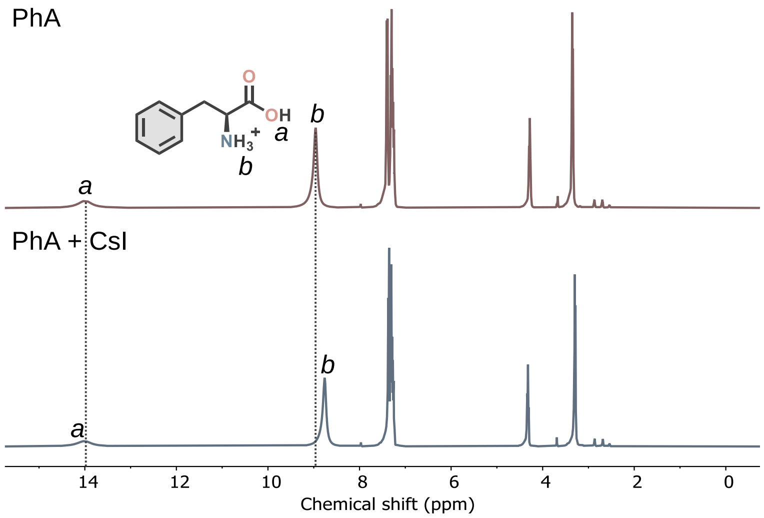


**Supplementary Fig. S3:** $^1$H NMR spectra of PhA and PhA + CsI dissolved in DMSO-$d_6$ and DMF-$d_7$ (v:v, 1:3) mixed solvent.

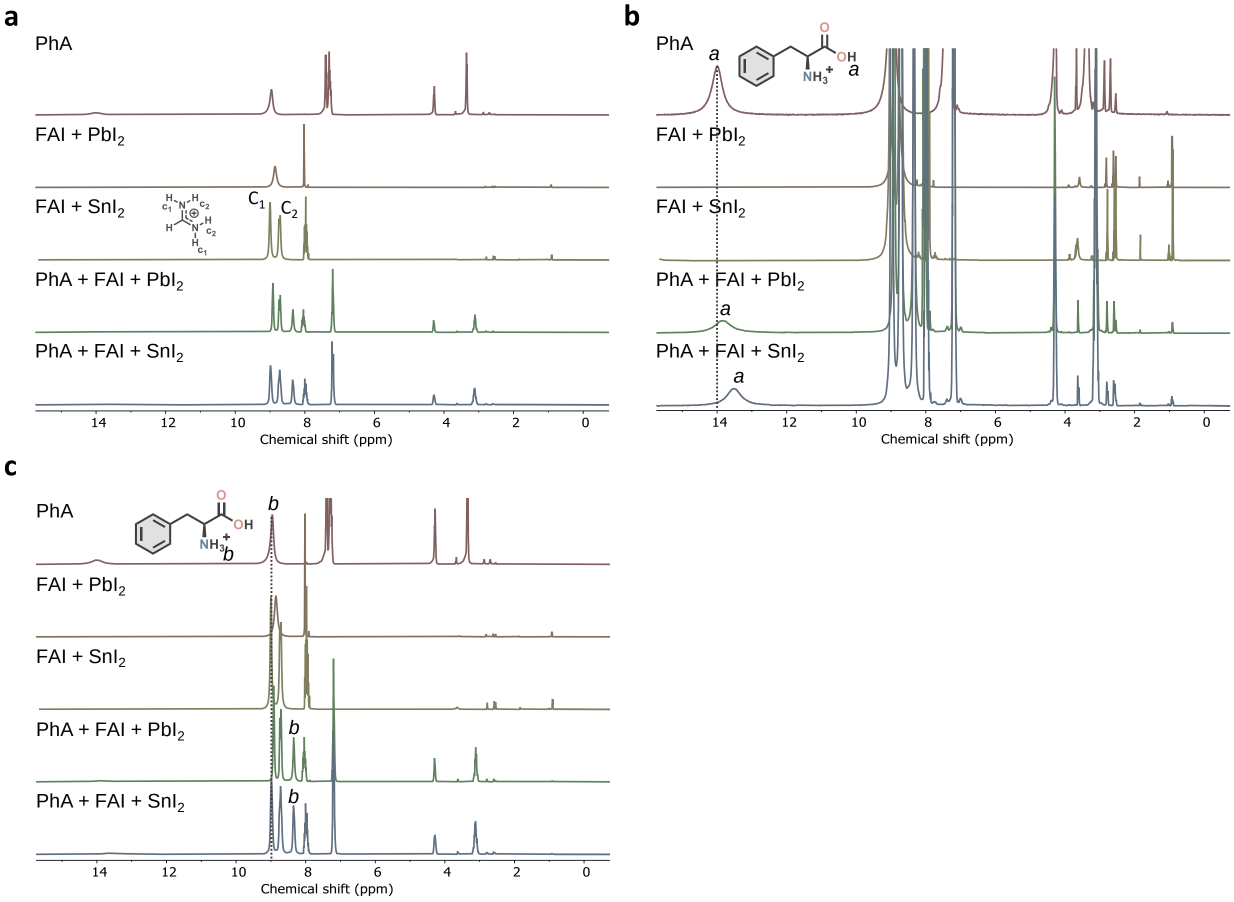


**Supplementary Fig. S4: a,** $^1$H NMR spectra of PhA, FAI + $PbI_2$, FAI + $SnI_2$, PhA + FAI + $PbI_2$, and PhA + FAI + $SnI_2$ dissolved in DMSO-$d_6$ and DMF-$d_7$ (v:v, 1:3) mixed solvent. Magnified show of **a** to present the **b,** carboxylic acid, and **c,** ammonium protons of PhA.

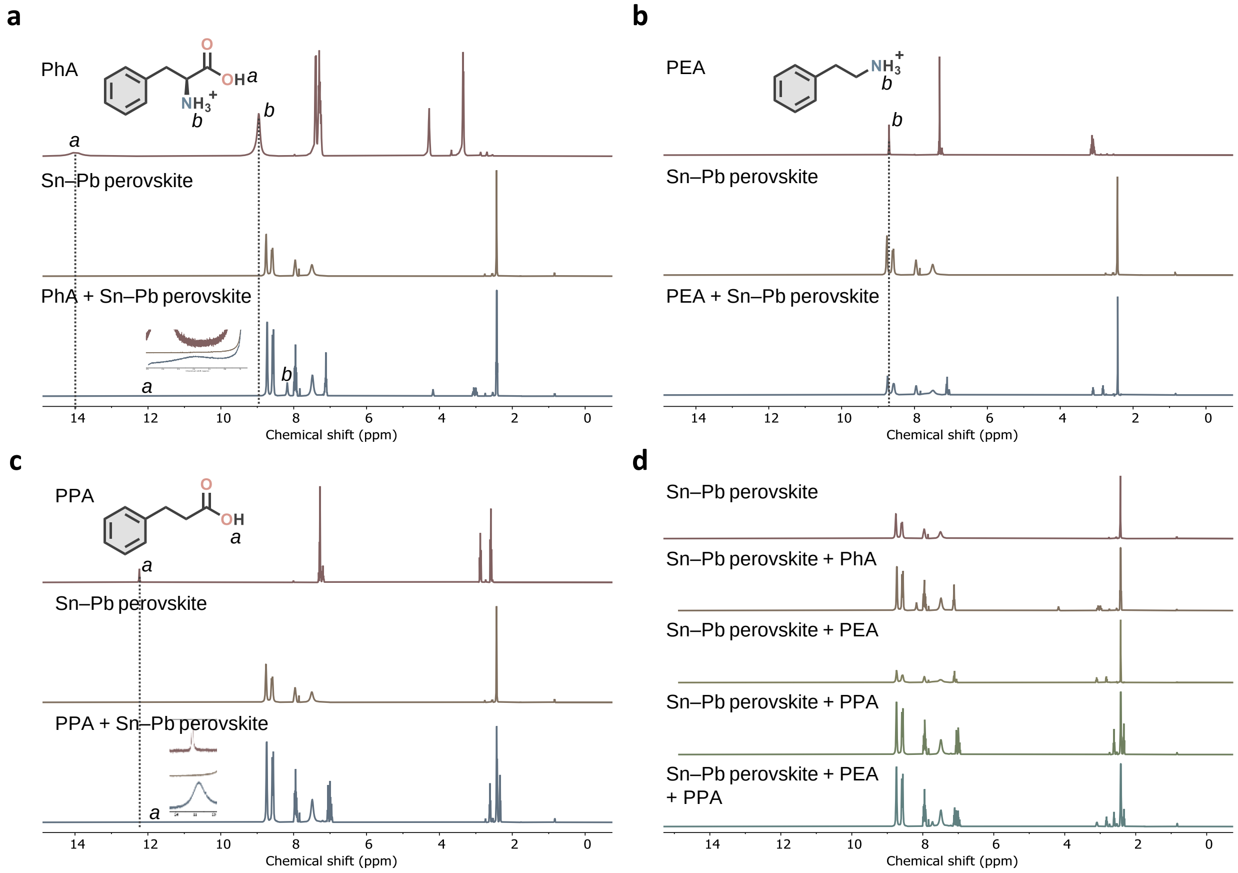


**Supplementary Fig. S5:** $^{1}$H NMR spectra of **a,** PhA, mixed Sn–Pb perovskite precursor, and PhA + mixed Sn–Pb perovskite precursor, (insert provides the magnified show for the proton signals) **b,** PEA, mixed Sn–Pb perovskite precursor, and PEA + mixed Sn–Pb perovskite precursor, **c,** PPA, mixed Sn–Pb perovskite precursor, and PPA + mixed Sn–Pb perovskite precursor, (insert provides the magnified show for the proton signals) and **d,** mixed Sn–Pb perovskite precursor individually with PhA, PEA, PPA, and PEAPPA dissolved in DMSO-$d_6$ and DMF-$d_7$ (v:v, 1:3) mixed solvent. For ease of comparison, the spectra of the materials are presented repeatedly.

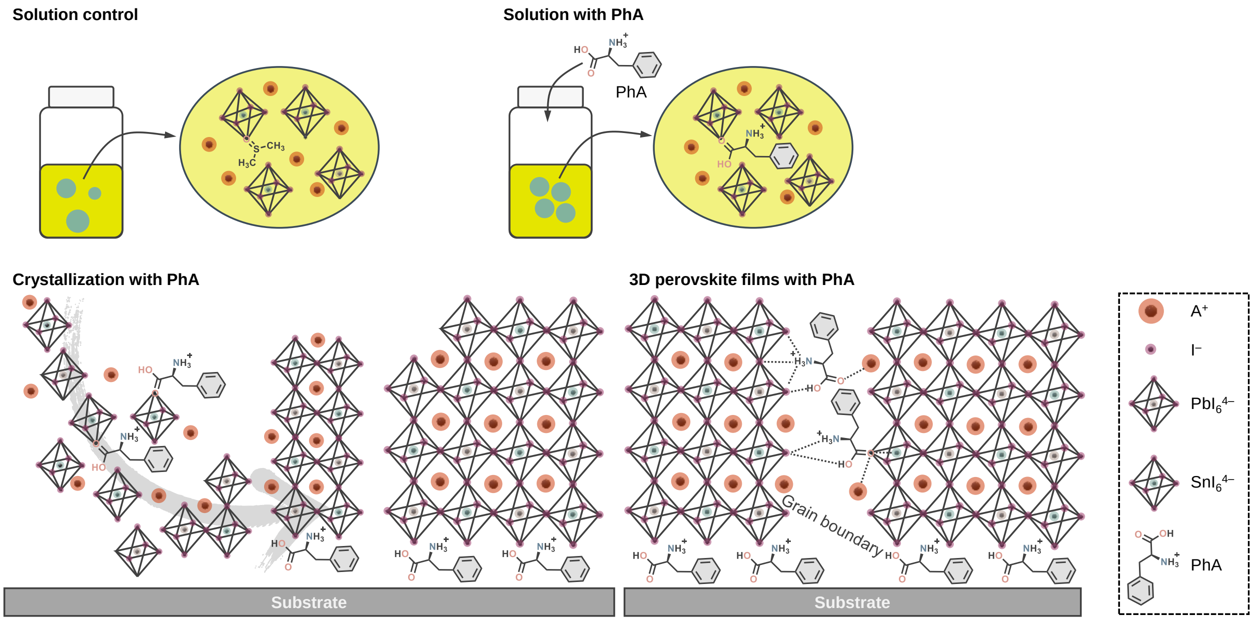


**Supplementary Fig. S6:** Schematic illustration of the impact of PhA during the solution stage, as well as its possible interactions and location during crystallization and in the as-crystalised perovskite films. As discussed in the main text, Sn-containing species assemble to a higher degree in the perovskite precursor solution system due to the rapid reaction of the Sn(II) precursor with the A-site cation precursors. Upon the addition of PhA, the interactions between the Sn(II) species and the DMSO solvent are partially replaced by PhA, leading to the formation of colloidal particles with more uniform and larger sizes, as suggested by solution characterizations, NMR and DLS, and DFT calculations. When using the PhA solution for film deposition, the crystallization process is altered by newly introduced chemical interactions, such as coordination and hydrogen bonding. This modification slows down the initial crystallization dynamics of the Sn(II)-based compounds during the ion exchange process, providing a better balance between the dynamics of Sn(II) and Pb(II) within the crystalline domains. Ultimately, this could result in atomically homogeneous 3D Sn–Pb perovskites after annealing.

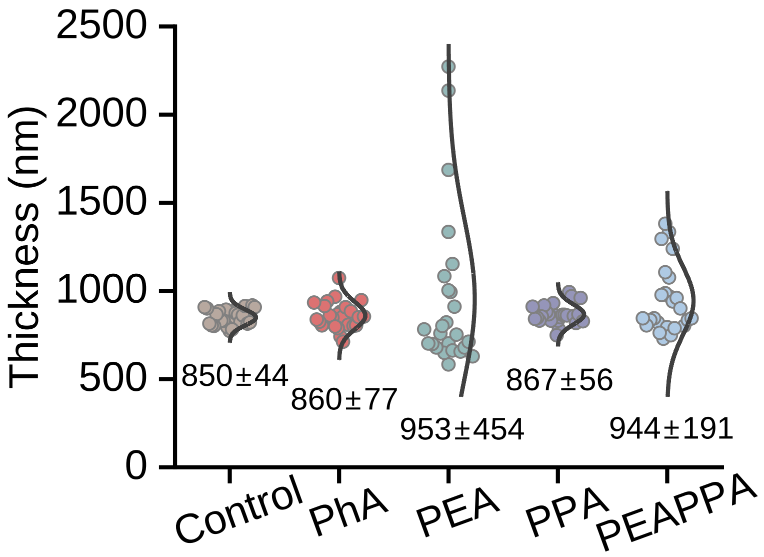


**Supplementary Fig. S7:** Distribution of the thickness of perovskite films fabricated on the FTO/PEDOT:PSS substrate. 25 different points were taken from five different cross-sectional SEM images of the devices fabricated under each condition.

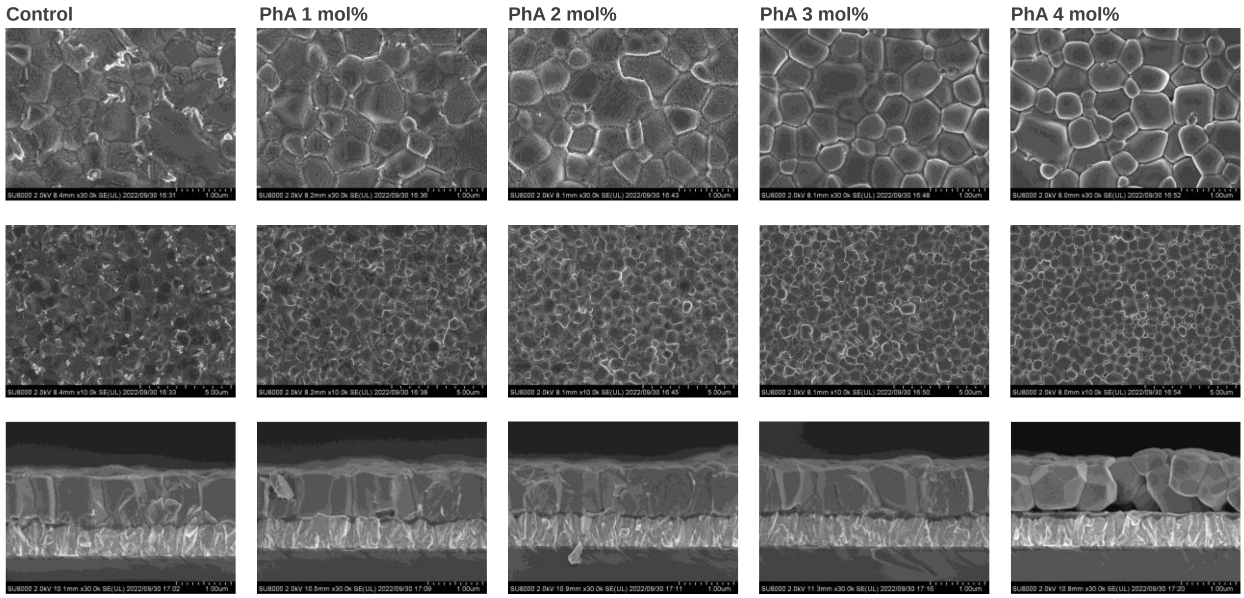


**Supplementary Fig. S8:** Top-view and cross-sectional SEM images of the perovskite films fabricated on PEDOT:PSS-coated FTO/glass substrates with no PhA (control) and 1, 2, 3, and 4 mol% of PhA added into the precursor solution.

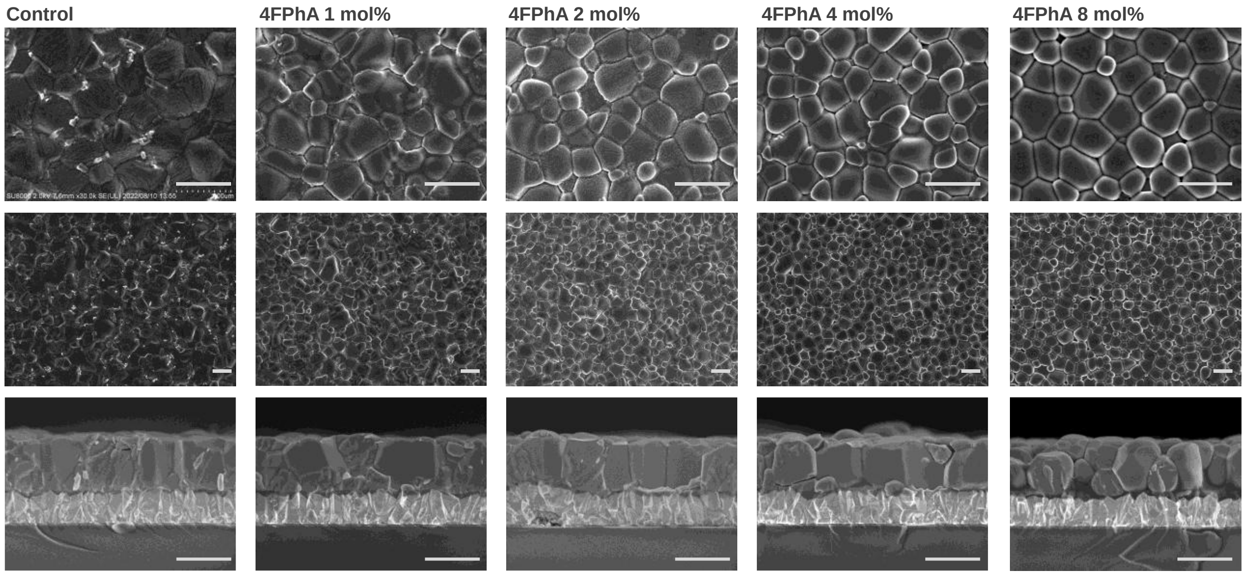


**Supplementary Fig. S9:** Top-view and cross-sectional SEM images of the perovskite films fabricated on PEDOT:PSS-coated FTO/glass substrates with no 4FPhA (control) and 1, 2, 4, and 8 mol% of 4FPhA added into the precursor solution. Scale bar: 1 μm.

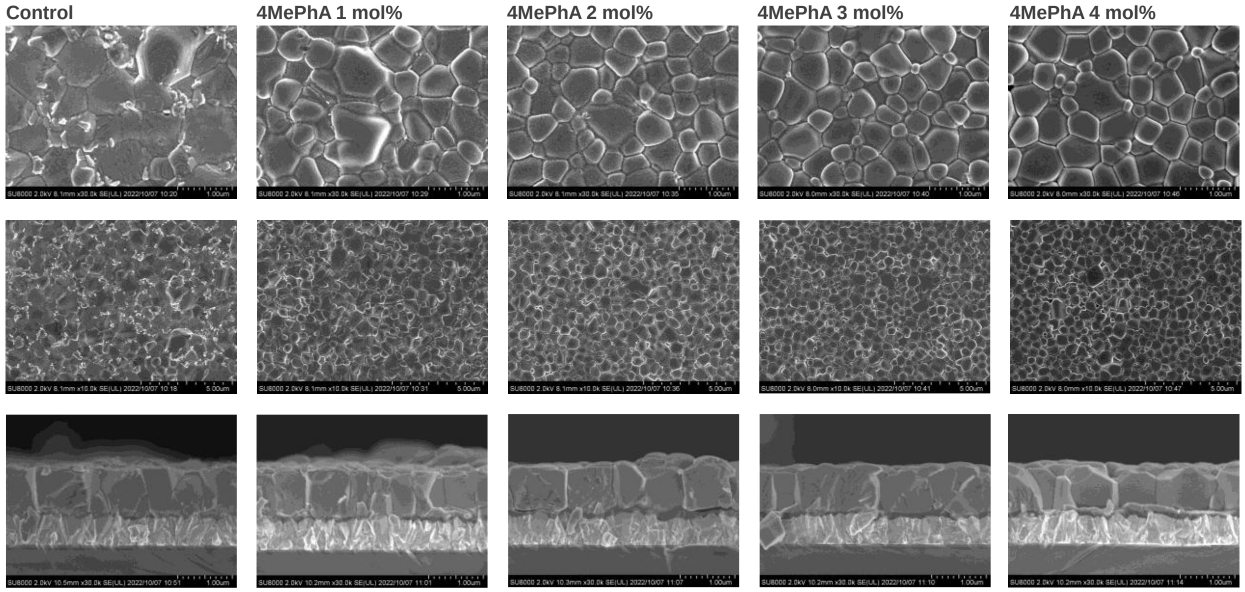


**Supplementary Fig. S10:** Top-view and cross-sectional SEM images of the perovskite films fabricated on PEDOT:PSS-coated FTO/glass substrates with no 4MePhA (control) and 1, 2, 3, and 4 mol% of 4MePhA added into the precursor solution.

a

b

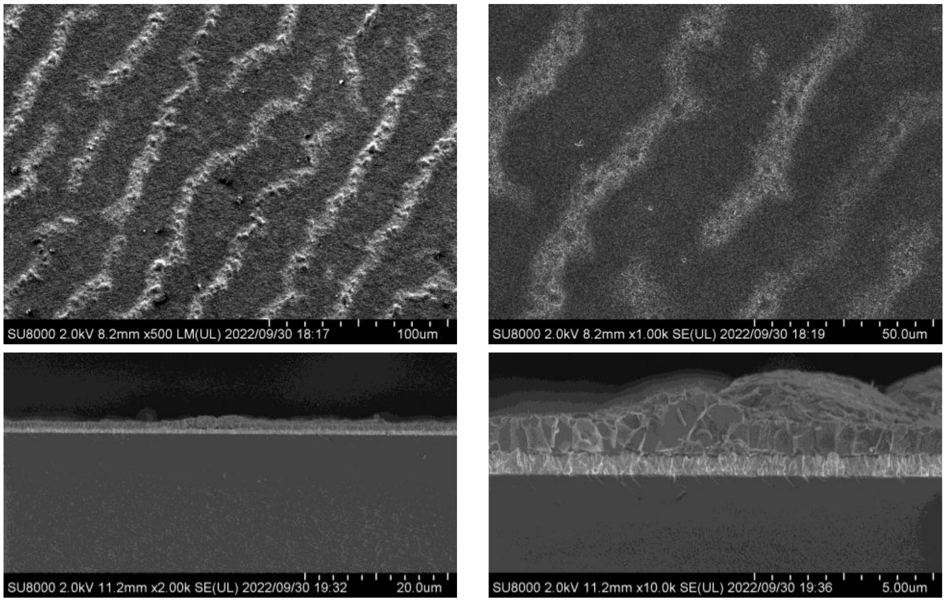


**Supplementary Fig. S11: a,** Top-view and **b,** cross-sectional SEM images of the PEA-treated perovskite films fabricated on PEDOT:PSS-coated FTO/glass substrates.

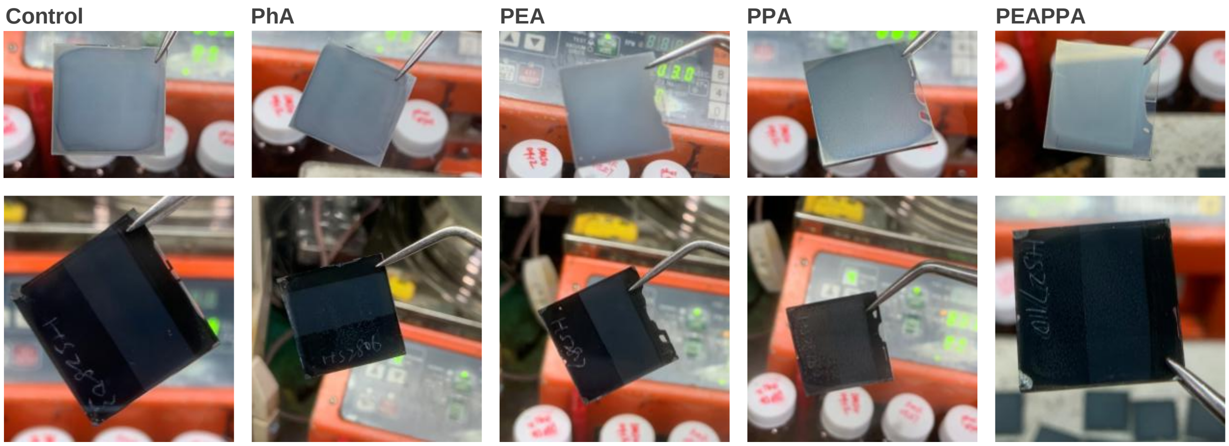


**Supplementary Fig. S12:** Photographs of the perovskite films fabricated on the FTO/PEDOT:PSS substrates with the view from the top surface (top) and glass (bottom) sides of the samples. The substrate layout is 25 mm by 25 mm.

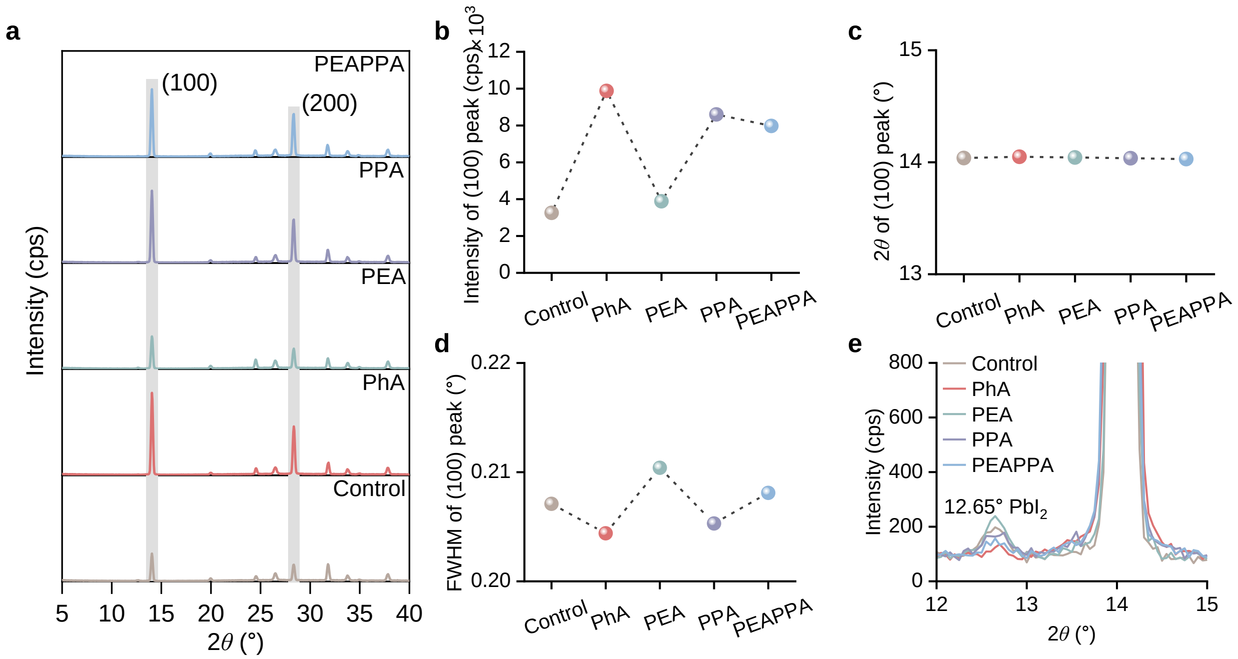


**Supplementary Fig. S13: a,** XRD patterns of control, PhA, PEA, PPA, and PEAPPA perovskite films. **b,** Intensity and **c,** 2$\theta$ values of the (100) peak of the films. **d,** Full width at half maximum (FWHM) of the corresponding (100) peaks. **e,** Magnified XRD with 2$\theta$ from 12 to 15° for showing the $PbI_2$ peaks. A thin layer of PMMA was coated onto the perovskite films to prevent oxidation during the measurement. In general, we observed that the films are dominated by the (100) and (200) peaks with 2$\theta$ values of 14.05 and 28.33°, respectively, with no considerable peak shift between the films. The d-spacing value of the (100) plane for all the samples is calculated to be 6.30 Å based on Bragg's Law, the wavelength of the X-ray is 1.5406 Å generated from the Cu $K_\alpha$ source. Based on the fitting of the XRD patterns, the obtained cell parameters are a = b = c = 6.29830 Å, α = β = γ = 90° with the cubic crystal system under the space group of $Pm\bar{3}m$ (COD number: 4128767) based on the fitting of the XRD patterns. The average crystallite size, estimated using the Scherrer formula (Dp = (0.94*λ)/(β*Cosθ), where, Dp = average crystallite size, β = Line broadening (FWHM) in radians, θ = Bragg angle, λ = X-ray wavelength), is about 40.35, 40.88, 39.72, 40.70, and 40.16 nm for the control, PhA, PEA, PPA, and PEAPPA perovskite films, respectively. No detectable low-dimensional phases form in any of the films examined even in the PEA case – a well-reported 2D Ruddlesden–Popper phase spacer cation –from the 1D and 2D XRD results. In addition, the PhA-containing films show considerably reduced $PbI_2$ signals with the 2$\theta$ value of 12.65°.

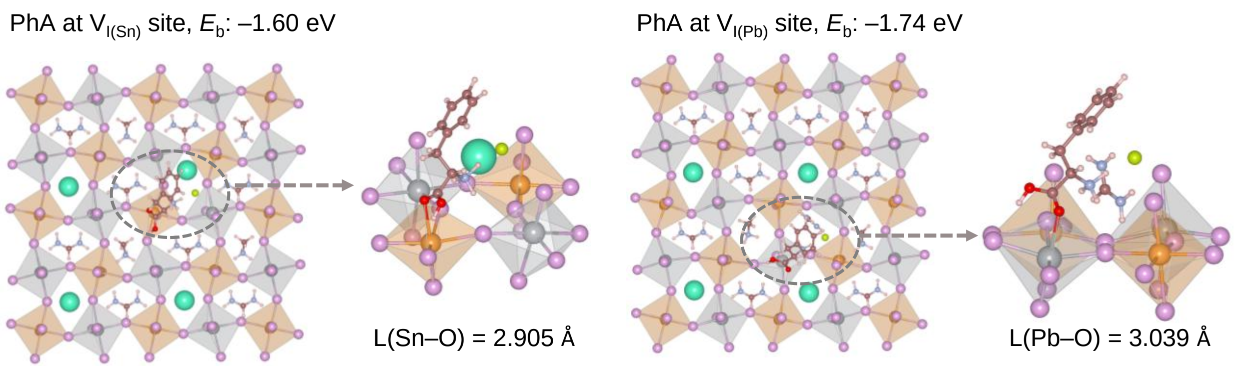


**Supplementary Fig. S14:** Adsorption configuration of PhA at the perovskite surface with $V_{I(Sn)}$ and $V_{I(Pb)}$ along with the resultant binding energies. Pb, grey; Sn, orange; Cs, green; N, blue; I, pink; Cl, apple green; O, red; C, brown; H, light pink.

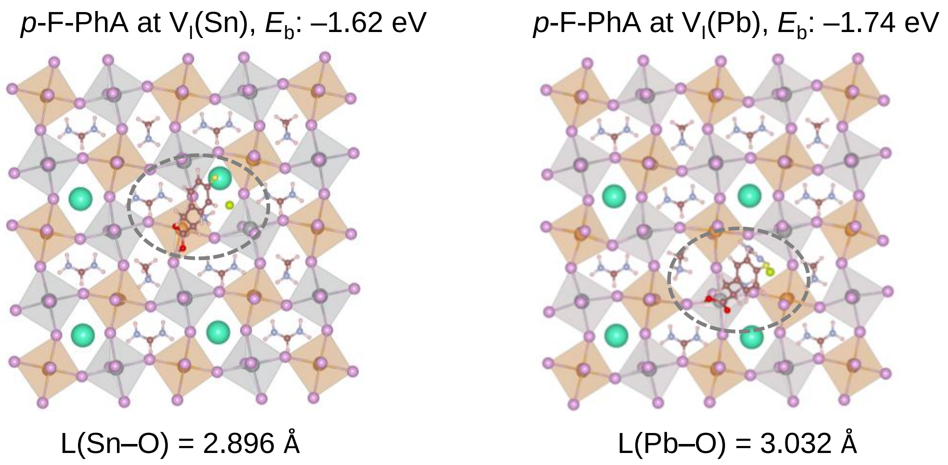


**Supplementary Fig. S15:** Adsorption configuration of pFPhA (4FPhA) at the perovskite surface with $V_{I(Sn)}$ and $V_{I(Pb)}$ along with the resultant binding energies. Pb, grey; Sn, orange; Cs, green; N, blue; I, pink; Cl, apple green; O, red; C, brown; H, light pink.

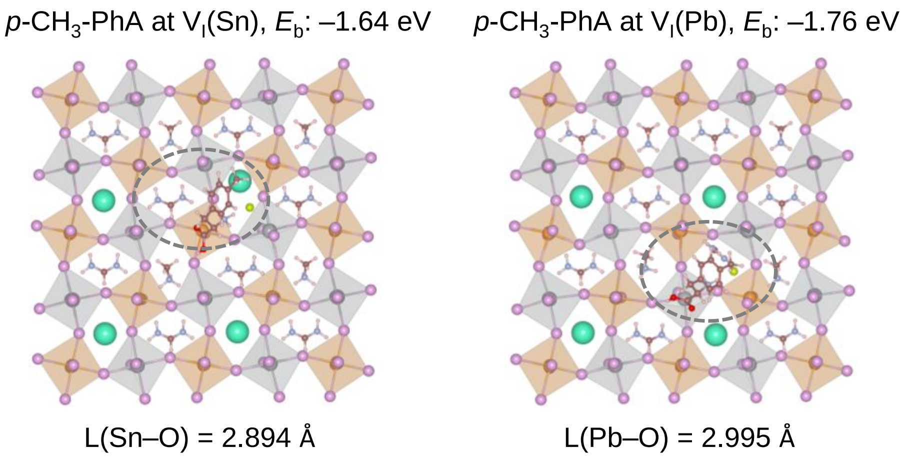


**Supplementary Fig. S16:** Adsorption configuration of pMePhA (4MePhA) at the perovskite surface with $V_{I(Sn)}$ and $V_{I(Pb)}$ along with the resultant binding energies. Pb, grey; Sn, orange; Cs, green; N, blue; I, pink; Cl, apple green; O, red; C, brown; H, light pink.

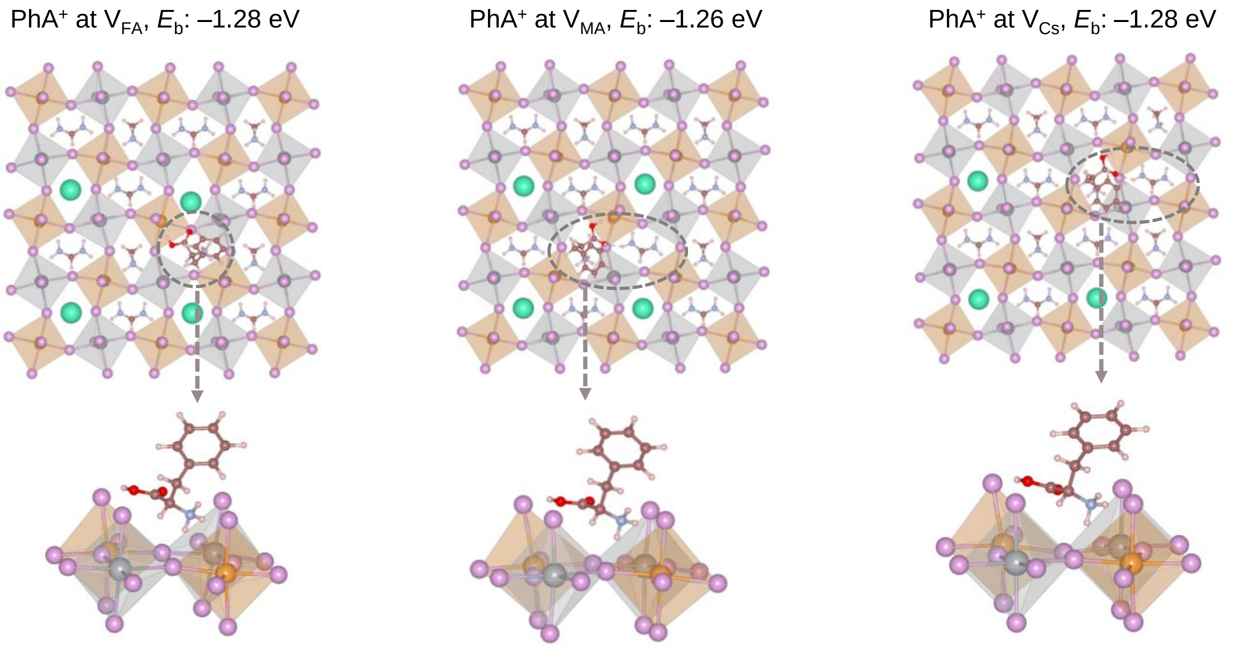


**Supplementary Fig. S17:** Adsorption configuration of $PhA^+$ at the perovskite surface with $V_{FA}$, $V_{MA}$, and $V_{Cs}$ along with the resultant binding energies. Pb, grey; Sn, orange; Cs, green; N, blue; I, pink; O, red; C, brown; H, light pink.

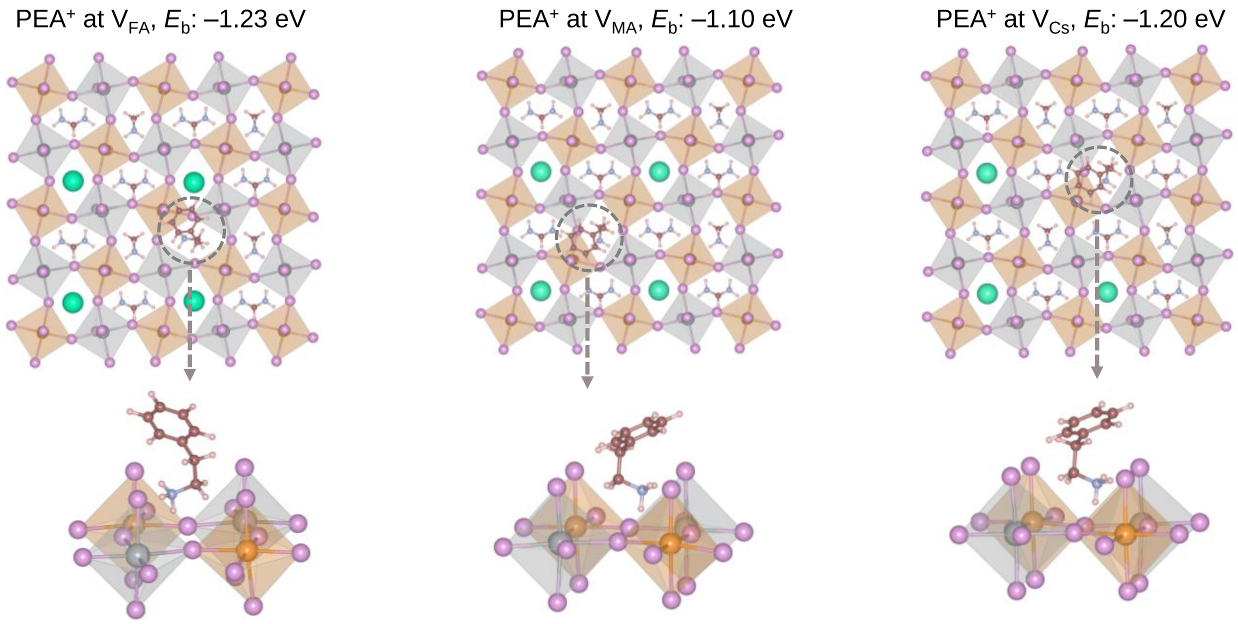


**Supplementary Fig. S18:** Adsorption configuration of PEA$^+$ at the perovskite surface with $V_{FA}$, $V_{MA}$, and $V_{Cs}$ along with the resultant binding energies. Pb, grey; Sn, orange; Cs, green; N, blue; I, pink; O, red; C, brown; H, light pink.

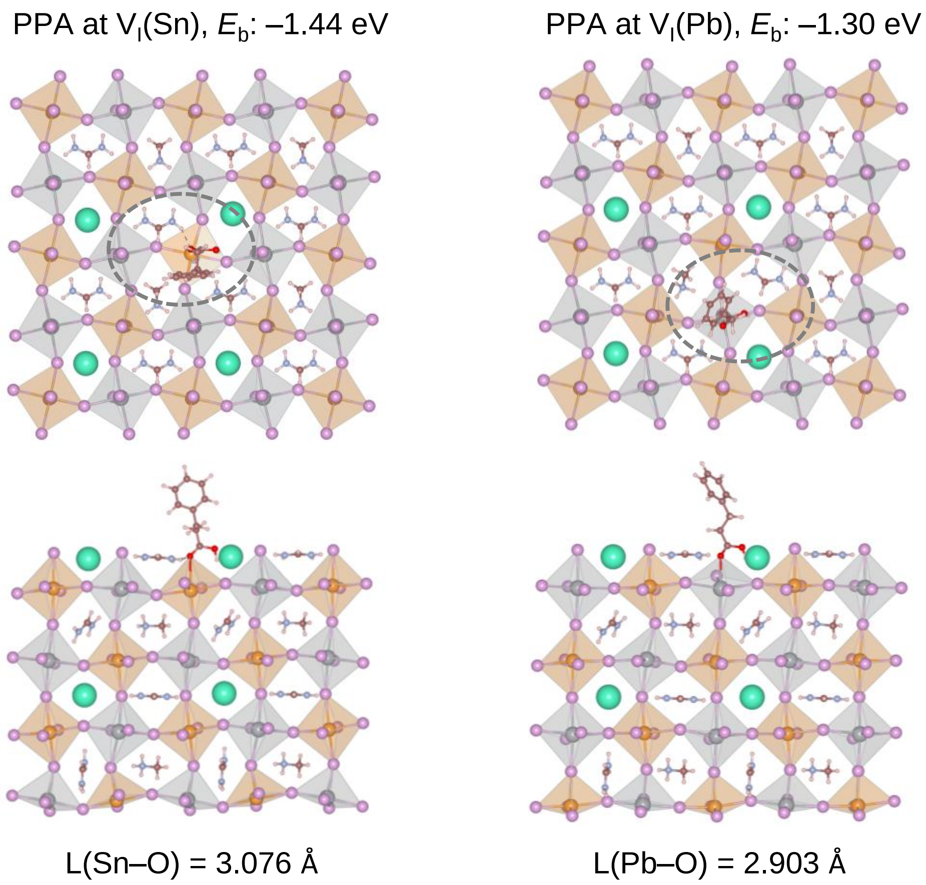


**Supplementary Fig. S19:** Adsorption configuration of PPA at the perovskite surface with $V_{I(Sn)}$ and $V_{I(Pb)}$ along with the resultant binding energies. Pb, grey; Sn, orange; Cs, green; N, blue; I, pink; O, red; C, brown; H, light pink. The length of Sn–O bond is longer than that of Pb–O, whereas a stronger interaction between PPA and uncoordinated Sn is found.

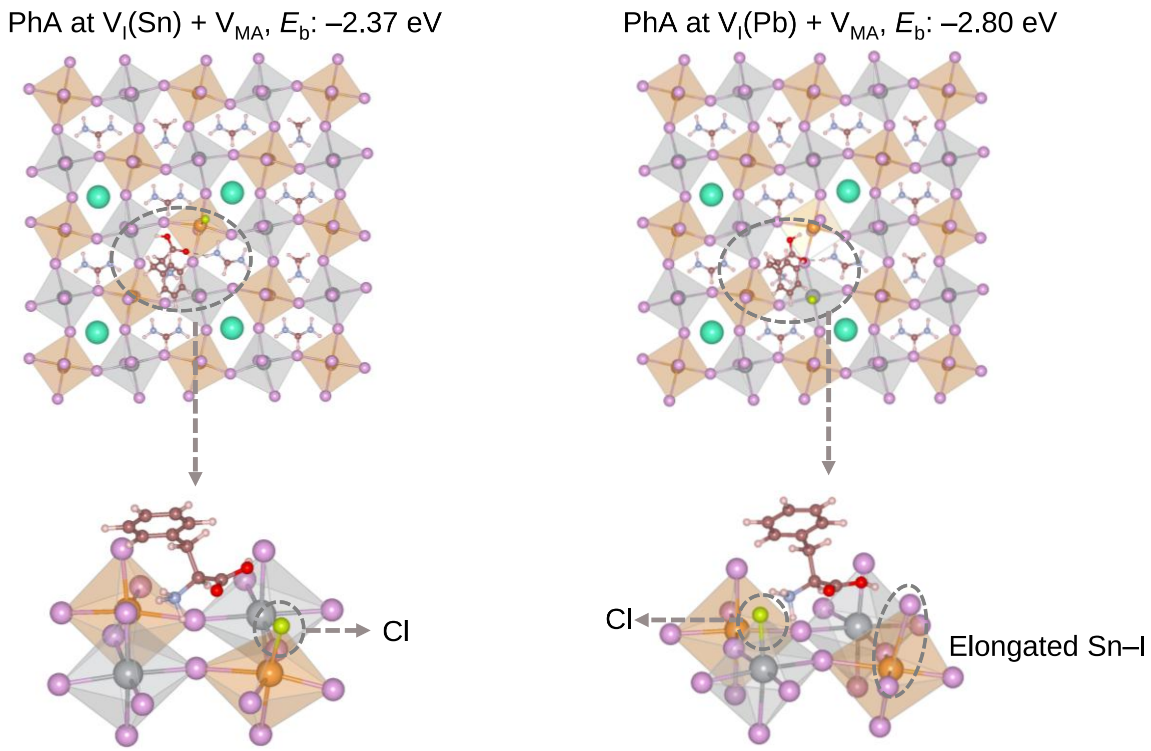


**Supplementary Fig. S20:** Adsorption configuration of PhA at the perovskite surface with the coexistence of neighbouring $V_{I(Sn/Pb)}$ and $V_{(A)}$ where $V_{MA}$ is chosen to show, along with the resultant binding energies. Pb, grey; Sn, orange; Cs, green; N, blue; I, pink; Cl, apple green; O, red; C, brown; H, light pink.

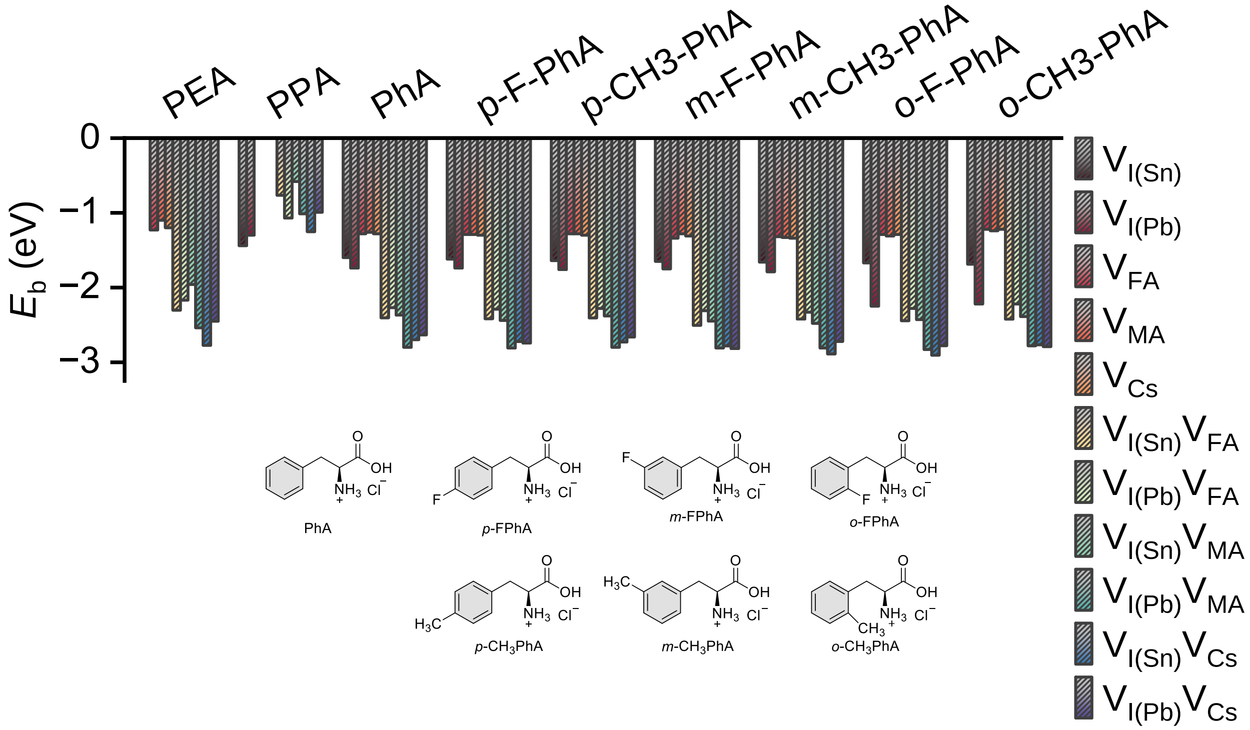


**Supplementary Fig. S21:** Calculated binding energies ($E_b$) between passivators and various vacancy defects. To extend the conclusion to the similar amino acid salts derivatives, we further evaluated the effect of *para*-, *meta*-, and *ortho-substitution* with F and $CH_3$ in PhA. It is found that the F or $CH_3$ substitution may improve the passivation effect, and the *ortho-substitution* performs the best for vacancies of iodine. When it comes to A-site defect passivation by PhA, the more negative binding energies than those by PEA indicate the improved passivation effect. Moreover, the substitution with F and $CH_3$ in PhA slightly further stabilized the defect system.

## UPS results

Effective passivation, especially under the context of facile Sn(II) oxidation, should be reflected in the electronic structure of the films. To this end, we conducted ultraviolet photoelectron spectroscopy (UPS) measurements (**Fig. 2d and S22**). For films deposited with the addition of PhA, the value obtained from the valence band (VB) region suggests an upshift of the Fermi level with a value of 0.91 eV away from the valence band maximum (VBM), compared to the value of 0.73 eV for the control films, which agrees with the higher binding energy shifts of the XPS results (**Supplementary Fig. S23 and Supplementary Table S1**) that suggested a larger occupation of higher electronic states. The values for PEA, PPA, and PEAPPA films are 0.73, 0.87, and 0.75 eV, respectively. These results suggest that the carboxylic acid provides the films with a more n-type feature while the ammonium group shows less impact on the surface electronic structure of the films. This could be because of the enhanced stability of the Sn(II)-based species, i.e., less degree of oxidation and self-p-doping, thanks to the effective binding of –COOH group, and the binding energy is slightly higher for PhA than that of PPA as unveiled by the DFT calculations. Incidentally, an n-type surface would benefit the electron extraction at the interface with the electron transport layer, $C_{60}$, in the p-i-n devices.

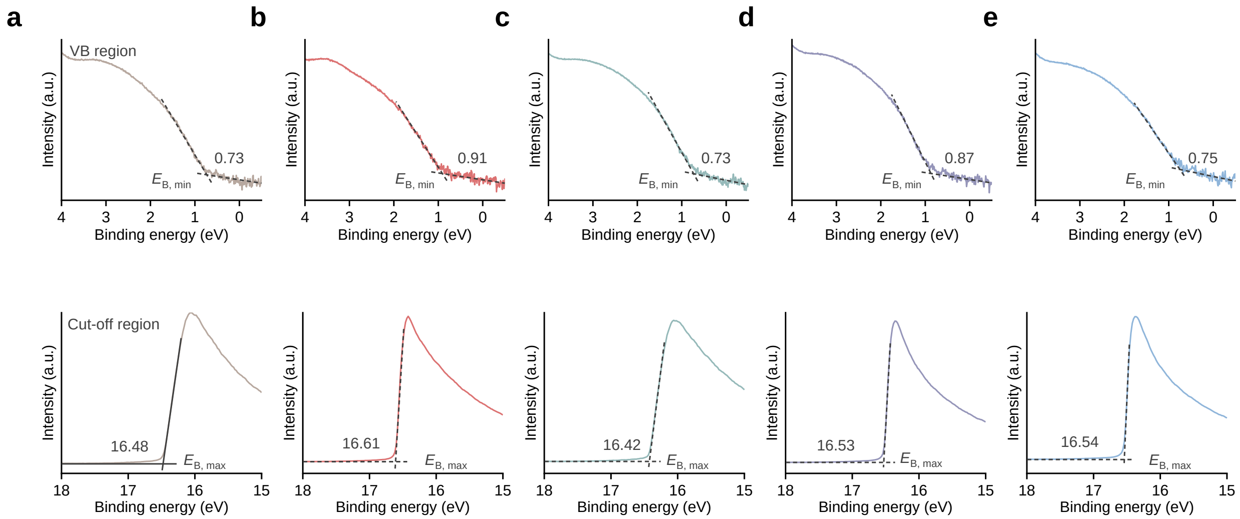


**Supplementary Fig. S22:** Valence band (VB, top, log scale) and secondary electron cut-off (bottom) regions of the UPS spectra for the **a,** control **b,** PhA, **c,** PEA, **d,** PPA, and **e,** PEAPPA films. The work function of the perovskite films is 21.22 eV – $E_{B, max}$, with $E_{B, max}$ determined from the secondary electron cut-off. The valence band maximum is 21.22 eV – ($E_{B, max}$ – $E_{B, min}$).

**XPS results**

For a better understanding of the perovskite films at an atomic level from the view of surface chemistry, we performed X-ray photoelectron spectroscopy (XPS) characterization on the films (**Supplementary Fig. S23**). By deconvoluting contributions from the Sn core levels, we fit the amounts of Sn(IV) as 19.2, 10.9, 18.9, 8.0, and 13.7% for the control, PhA-, PEA-, PPA-, and PEAPPA-modified films, respectively. The presence of less Sn(IV) in the PhA- and PPA-containing films could originate from the binding effect of the carboxylic acid to the Sn centres and/or improved film crystallinity as suggested by the NMR and XRD results. Compared to the control films, we observed considerable Sn core level shifts for the films fabricated with PhA and PPA to a higher binding energy, with a higher degree present for the Sn(IV) peaks, while the shift of the Pb peaks is relatively less noticeable (**Supplementary Table S1**). This was also observed in our previous study of mixed Sn–Pb perovskites[2]. In contrast, the films modified with PEA only show a small shift to lower energy for both the Sn and Pb peaks. These results suggest that the carboxylic acid group and the Sn species dominate the chemical interactions in the modified perovskite films, which is also suggested by the solution chemistry study.

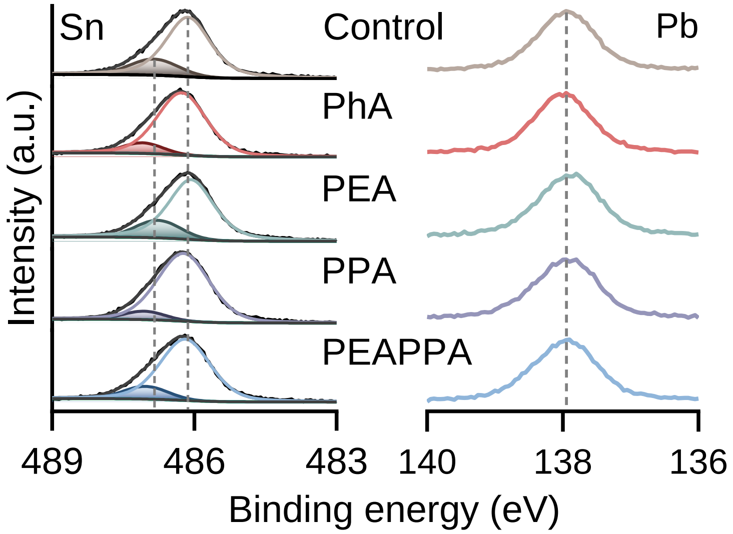


**Supplementary Fig. S23:** XPS spectra of the control and modified perovskite films for Pb $4f_{7/2}$ and Sn $3d_{5/2}$ core levels. For the Sn spectra here, the signal of each component was fitted based on the Shirley-type background. The shaded area represents the amount of $Sn^{4+}$.

**Supplementary Table S1:** The binding energy of the $Sn^{4+}$ and $Sn^{2+}$ peaks and $Sn^{4+}/(Sn^{2+} + Sn^{4+})$ ratio from the deconvolution of the $3d_{5/2}$ XPS peak manifolds. The binding energy of the $Pb^{2+}$ peak from the deconvolution of the $4f_{7/2}$ XPS peak manifolds.

| Samples | $Sn^{4+}$ (eV) | $\Delta Sn^{4+}$ (eV) | $Sn^{2+}$ (eV) | $\Delta Sn^{2+}$ (eV) | $Sn^{4+}/(Sn^{4+} + Sn^{2+})$ (%) | $Pb^{2+}$ (eV) | $\Delta Pb^{2+}$ |
|---|---|---|---|---|---|---|---|
| Control | 486.85 | – | 486.13 | – | 19.2 | 137.94 | – |
| PhA | 487.10 | 0.25 | 486.27 | 0.14 | 10.9 | 137.99 | 0.05 |
| PEA | 486.77 | –0.09 | 486.07 | –0.06 | 18.9 | 137.89 | –0.05 |
| PPA | 487.06 | 0.21 | 486.23 | 0.10 | 8.0 | 137.94 | 0 |
| PEAPPA | 487.03 | 0.26 | 486.19 | 0.06 | 13.7 | 137.96 | 0.02 |

**Supplementary Table S2:** Parameters extracted from the light intensity-dependent PLQE measurements. The external $k_{rad}$ resulting from our fits is significantly lower than observed in other (neat lead) perovskites. This could be partially due to a low escape probability but is not yet fully understood. The errors from fitting (calculated by inverting the second derivative matrix of fit quality) are large for some parameters. This is likely due to a high degree of multicollinearity between some variables, which could be improved by better-fitting approaches in the future.

| Sample | Control | PhA |
|---|---|---|
| $k_{trap,shallow}$ ($s^{-1}$) | $(2.3 \pm 2.9) \times 10^6$ | $(9.6 \pm 7.9) \times 10^4$ |
| $K_{trap,deep}$ ($s^{-1}$) | $(4.7 \pm 1.2) \times 10^4$ | $(1.4 \pm 1.3) \times 10^4$ |
| $E_{T,shallow}$ (eV) | 0.050 eV | |
| $k_{rad,ext}$ ($cm^3\ s^{-1}$) | $(3.3 \pm 0.1) \times 10^{-13}$ | |
| $K_{aug}$ ($cm^6\ s^{-1}$) | $10^{-28}$ (fixed) | |

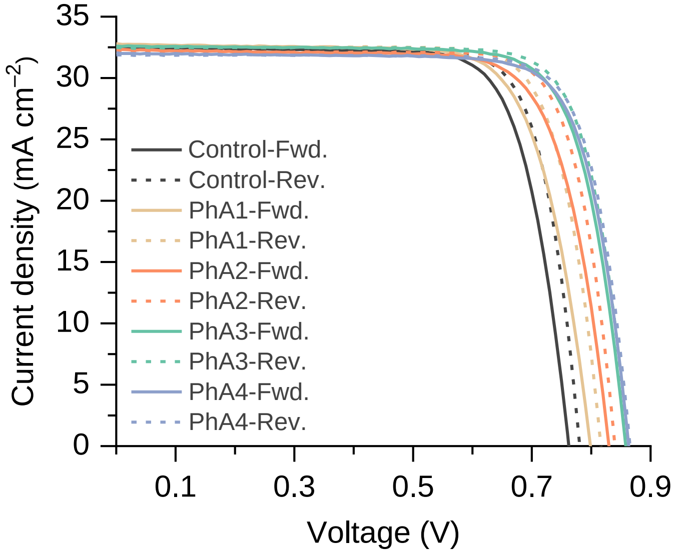


| Samples | $J_{SC}$ (mA cm$^{-2}$) | $V_{OC}$ (V) | FF | PCE (%) |
|---|---|---|---|---|
| Control-Fwd. | 32.49 | 0.76 | 0.76 | 18.80 |
| Control-Rev. | 32.30 | 0.78 | 0.79 | 19.82 |
| PhA1-Fwd. | 32.80 | 0.80 | 0.74 | 19.43 |
| PhA1-Rev. | 32.54 | 0.82 | 0.78 | 20.76 |
| PhA2-Fwd. | 32.35 | 0.83 | 0.75 | 20.19 |
| PhA2-Rev. | 32.12 | 0.84 | 0.79 | 21.33 |
| PhA3-Fwd. | 32.60 | 0.86 | 0.77 | 21.59 |
| PhA3-Rev. | 32.47 | 0.86 | 0.79 | 22.13 |
| PhA4-Fwd. | 32.06 | 0.86 | 0.78 | 21.49 |
| PhA4-Rev. | 31.88 | 0.87 | 0.79 | 21.85 |

**Supplementary Fig. S24:** Representative *J*–*V* curves and cell parameters of the devices fabricated with the perovskite films deposited without (control) and with the addition of 1, 2, 3, and 4 mol% of PhA. The unencapsulated devices were measured in the $N_2$-filled glovebox under the illumination of simulated AM1.5G.

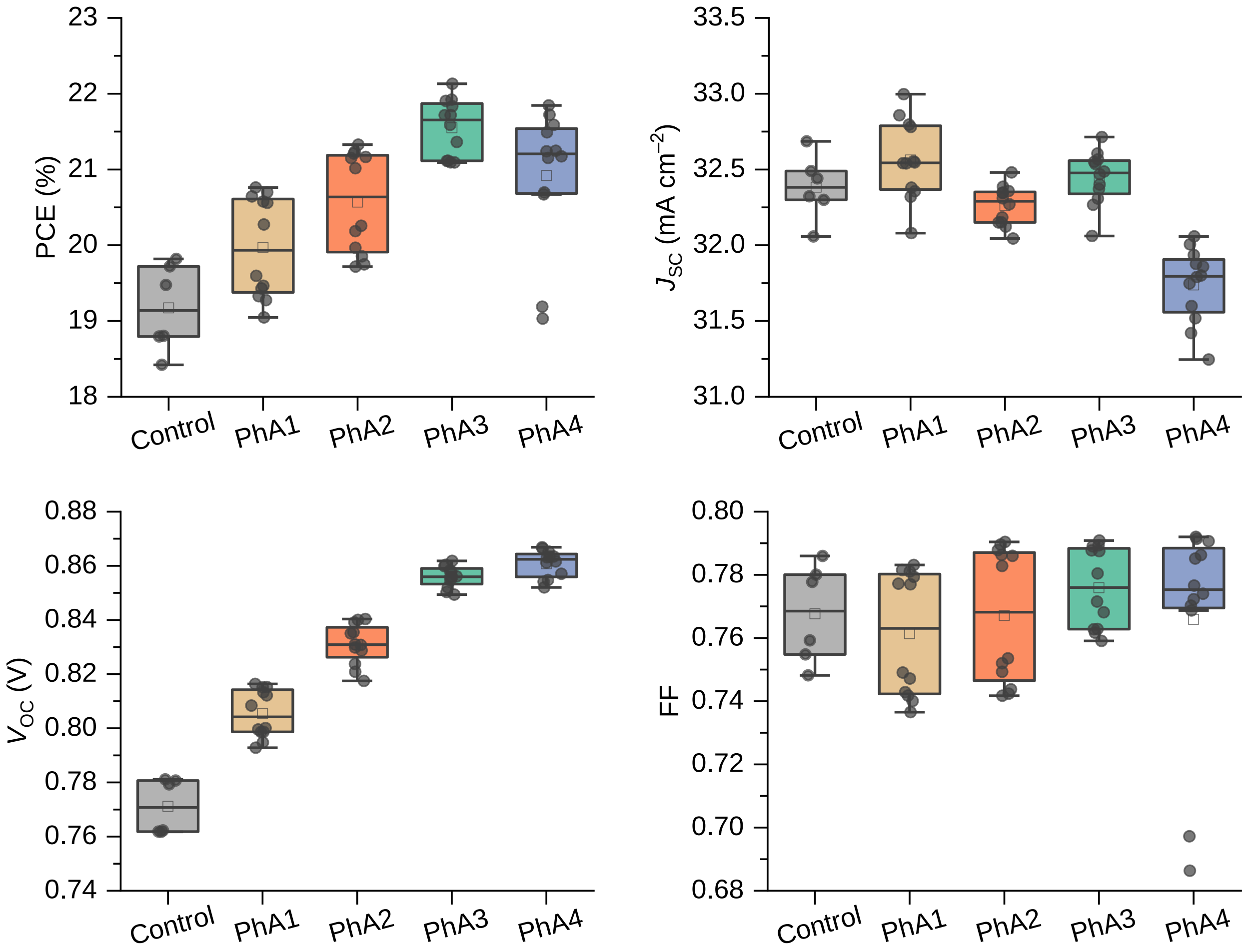


**Supplementary Fig. S25:** Distributions of the device parameters derived from forward and reverse *J–V* scans for the cells fabricated with the perovskite films deposited without (Control, three cells) and with the addition of 1, 2, 3, and 4 mol% of PhA (six cells for each). Data are mean ± s.e.m.

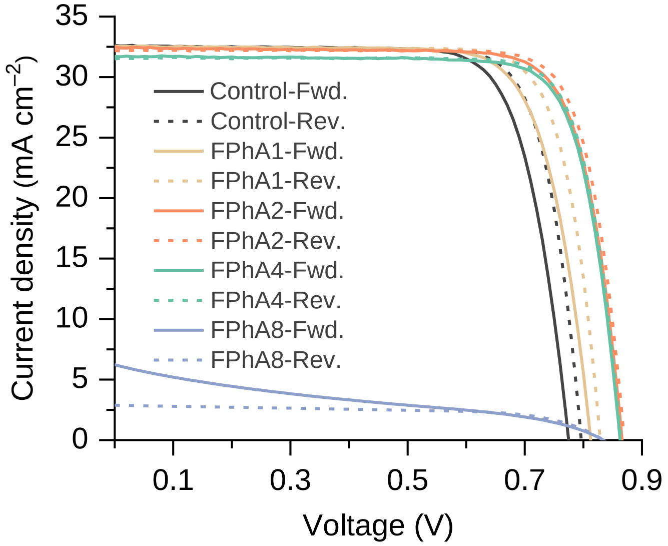


| Samples | $J_{SC}$ (mA cm$^{-2}$) | $V_{OC}$ (V) | FF | PCE (%) |
|---|---|---|---|---|
| Control-Fwd. | 32.62 | 0.77 | 0.76 | 19.27 |
| Control-Rev. | 32.33 | 0.80 | 0.79 | 20.43 |
| FPhA1-Fwd. | 32.55 | 0.81 | 0.76 | 20.22 |
| FPhA1-Rev. | 32.34 | 0.83 | 0.80 | 21.34 |
| FPhA2-Fwd. | 32.49 | 0.87 | 0.79 | 22.11 |
| FPhA2-Rev. | 32.19 | 0.87 | 0.81 | 22.57 |
| FPhA4-Fwd. | 31.70 | 0.86 | 0.80 | 21.75 |
| FPhA4-Rev. | 31.50 | 0.86 | 0.81 | 22.04 |
| FPhA8-Fwd. | 6.23 | 0.83 | 0.29 | 1.49 |
| FPhA8-Rev. | 2.88 | 0.83 | 0.62 | 1.49 |

**Supplementary Fig. S26:** Representative *J–V* curves and cell parameters of the devices fabricated with the perovskite films deposited without (control) and with the addition of 1, 2, 4, and 8 mol% of FPhA (4-fluoro-phenylalanine hydrochloride). The unencapsulated devices were measured in the $N_2$-filled glovebox under the illumination of simulated AM1.5G.

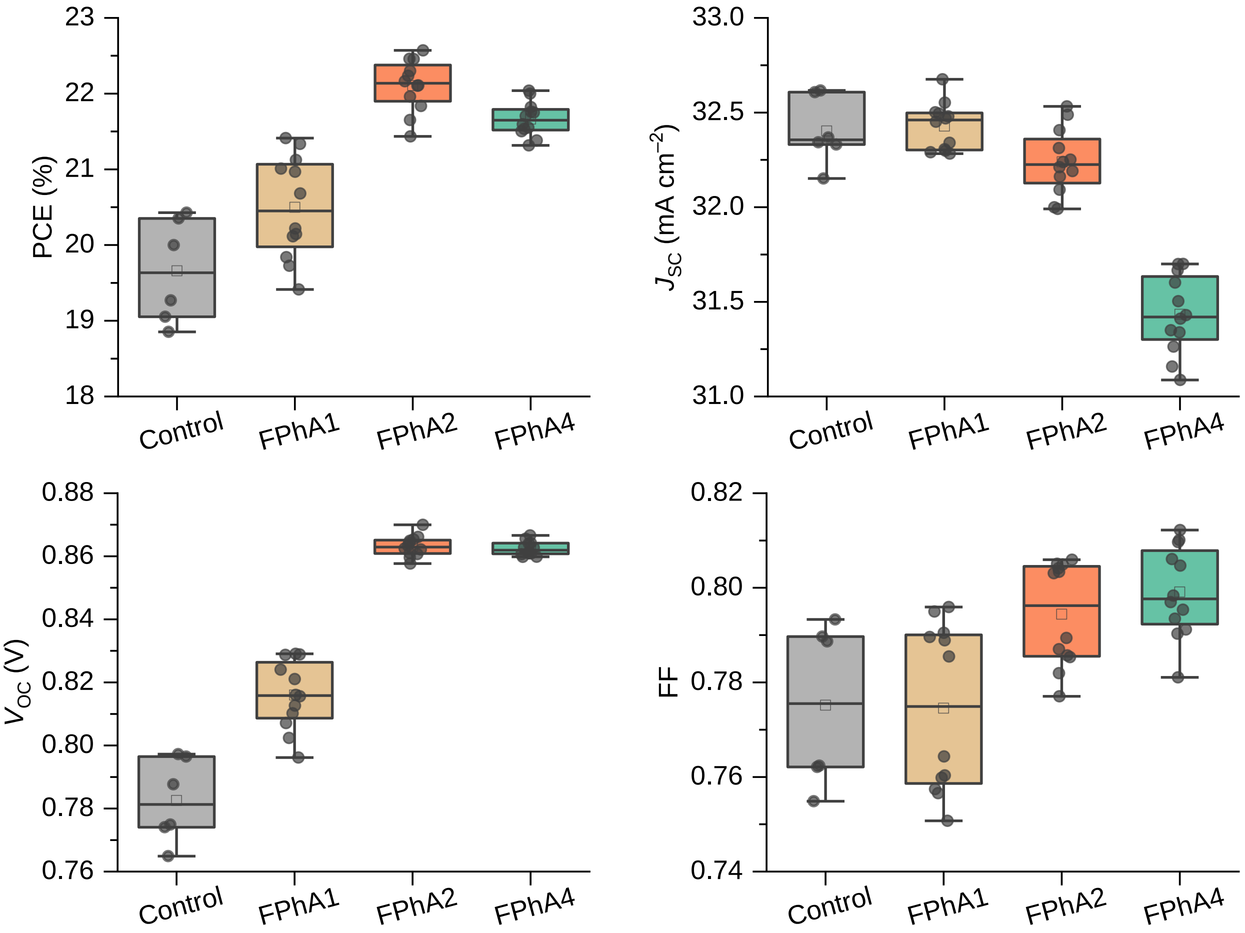


**Supplementary Fig. S27:** Distributions of the device parameters derived from forward and reverse *J–V* scans for the cells fabricated with the perovskite films deposited without (control, three cells) and with the addition of 1, 2, and 4 mol% of 4FPhA (six cells for each). The parameters for *J–V* scans of the 8 mol% 4FPhA-treated devices were not collected as the poor diode character of the PV devices shown in **Supplementary Fig. S26**. Data are mean ± s.e.m.

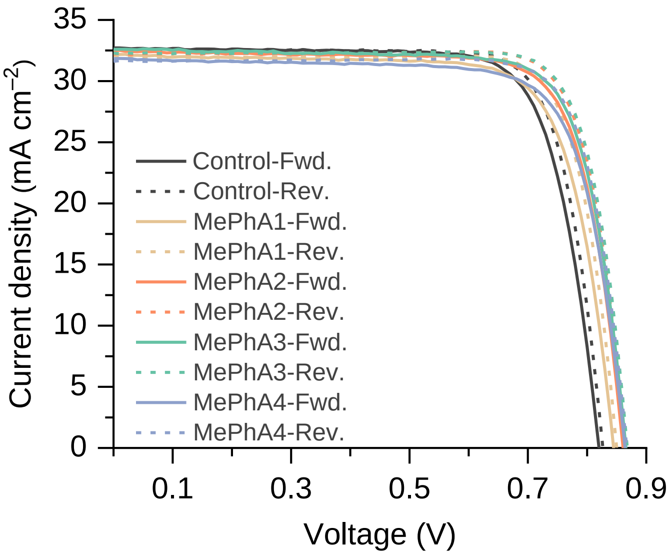


| Samples | $J_{SC}$ (mA cm$^{-2}$) | $V_{OC}$ (V) | FF | PCE (%) |
|---|---|---|---|---|
| **Control-Fwd.** | **32.71** | **0.82** | **0.76** | **20.48** |
| **Control-Rev.** | **32.56** | **0.83** | **0.79** | **21.18** |
| **MePhA1-Fwd.** | **32.20** | **0.84** | **0.76** | **20.60** |
| **MePhA1-Rev.** | **31.96** | **0.85** | **0.80** | **21.76** |
| **MePhA2-Fwd.** | **32.48** | **0.86** | **0.77** | **21.62** |
| **MePhA2-Rev.** | **32.20** | **0.86** | **0.81** | **22.49** |
| **MePhA3-Fwd.** | **32.59** | **0.87** | **0.78** | **21.90** |
| **MePhA3-Rev.** | **32.29** | **0.87** | **0.81** | **22.63** |
| **MePhA4-Fwd.** | **31.83** | **0.86** | **0.76** | **20.92** |
| **MePhA4-Rev.** | **31.61** | **0.87** | **0.80** | **21.98** |

**Supplementary Fig. S28:** Representative *J–V* curves and cell parameters of the devices fabricated with the perovskite films deposited without (control) and with the addition of 1, 2, 3, and 4 mol% of MePhA (4-methyl-phenylalanine hydrochloride). The unencapsulated devices were measured in the $N_2$-filled glovebox under the illumination of simulated AM1.5G.

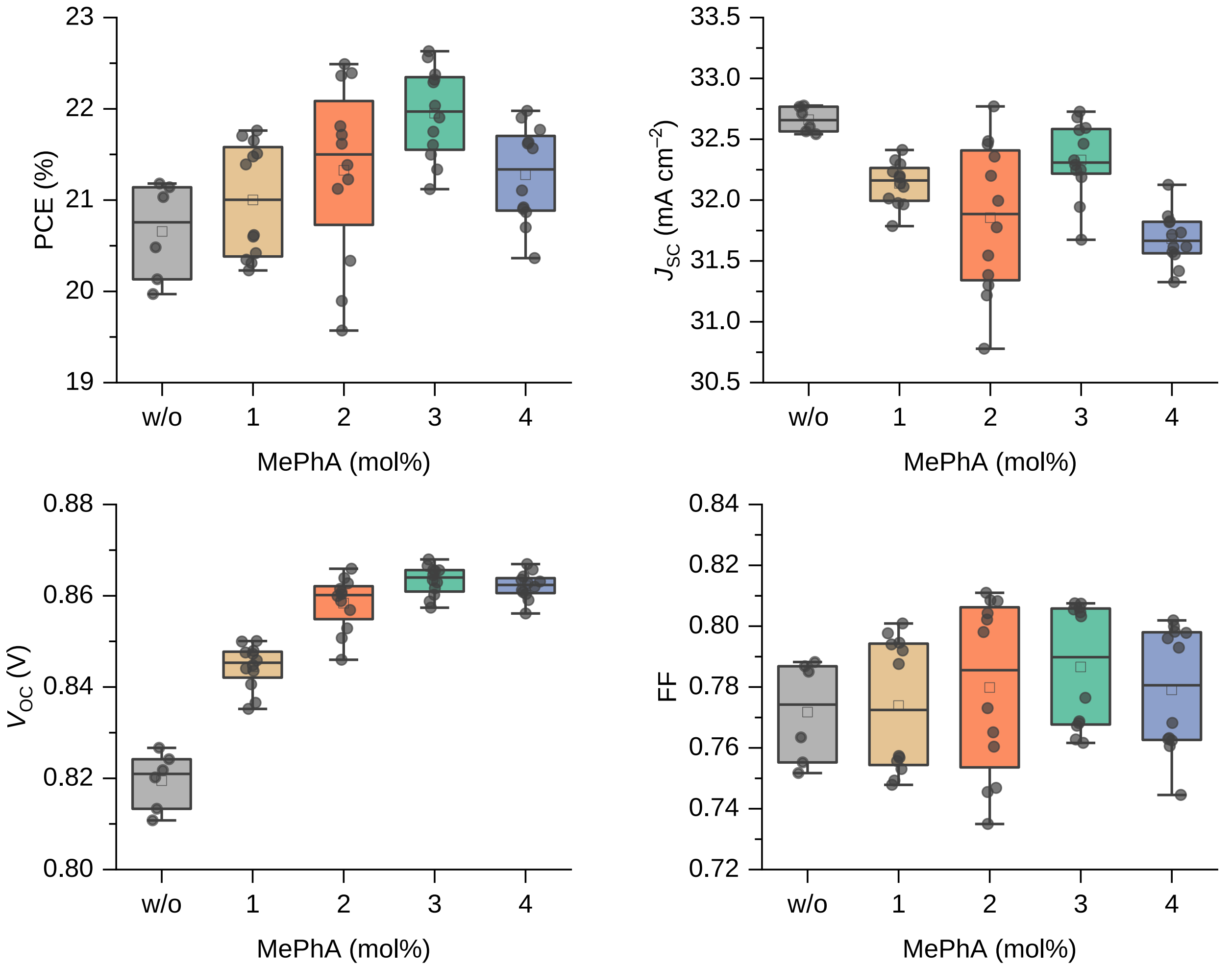


**Supplementary Fig. S29:** Distributions of the device parameters derived from forward and reverse *J–V* scans for the cells fabricated with the perovskite films deposited without (w/o, control, three cells) and with the addition of 1, 2, 3, and 4 mol% of 4MePhA (six cells for each). Data are mean ± s.e.m.

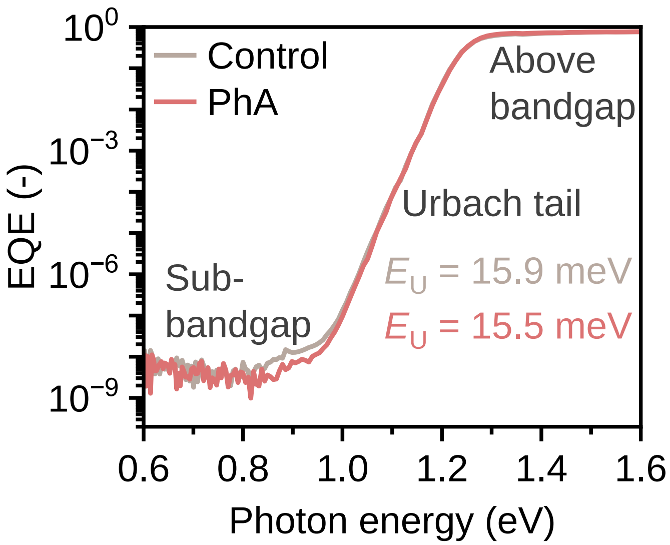


**Supplementary Fig. S30:** Semi-logarithmic plot of sensitive EQE spectra of the control and PhA narrow bandgap mixed Sn–Pb PSCs, divided into three regions: the above-bandgap, the band-edge (Urbach tail), and the sub-bandgap. The calculated Urbach energy is 15.9 and 15.5 meV for control and PhA-modified PSCs, respectively. Compared to the control sample, the PhA device shows a slightly lower EQE value at the sub-bandgap region (between photon energy of 0.8 – 1.0 eV).

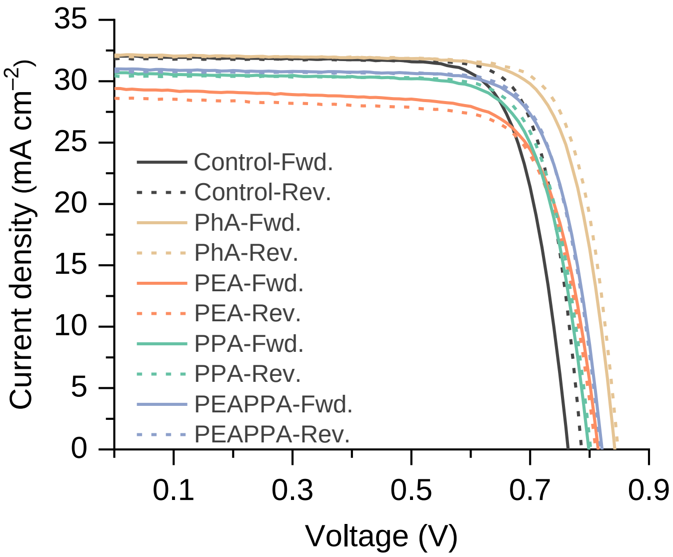


| Samples | $J_{SC}$ (mA cm$^{-2}$) | $V_{OC}$ (V) | FF | PCE (%) |
|---|---|---|---|---|
| Control-Fwd. | 32.04 | 0.76 | 0.76 | 18.62 |
| Control-Rev. | 31.82 | 0.79 | 0.79 | 19.81 |
| PhA-Fwd. | 32.14 | 0.84 | 0.77 | 20.85 |
| PhA-Rev. | 32.03 | 0.85 | 0.79 | 21.35 |
| PEA-Fwd. | 29.40 | 0.81 | 0.73 | 17.57 |
| PEA-Rev. | 28.61 | 0.81 | 0.75 | 17.29 |
| PPA-Fwd. | 30.67 | 0.80 | 0.75 | 18.44 |
| PPA-Rev. | 30.43 | 0.80 | 0.77 | 18.83 |
| PEAPPA-Fwd. | 31.00 | 0.82 | 0.76 | 19.37 |
| PEAPPA-Rev. | 30.78 | 0.82 | 0.78 | 19.57 |

**Supplementary Fig. S31:** Representative *J–V* curves and cell parameters of the devices fabricated with the perovskite films deposited without (control) and with the addition of PhA, PEA, PPA, and PEAPPA. The unencapsulated devices were measured in the $N_2$-filled glovebox under the illumination of simulated AM1.5G.

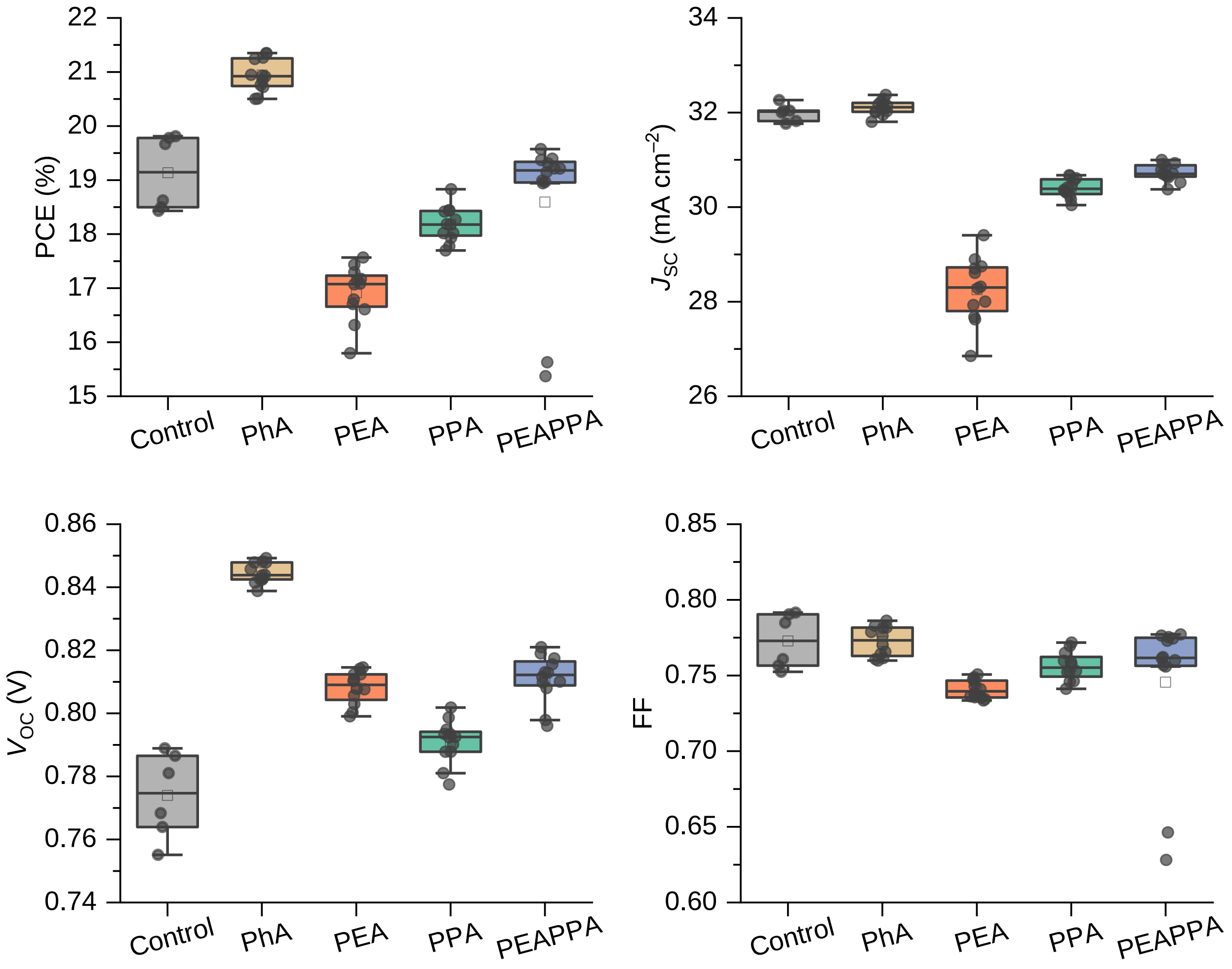


**Supplementary Fig. S32:** Distributions of the device parameters derived from forward and reverse *J*–*V* scans for the cells fabricated with the perovskite films deposited without (control, three cells) and with the addition of PhA, PEA, PPA, and PEAPPA (six cells for each). Data are mean ± s.e.m.

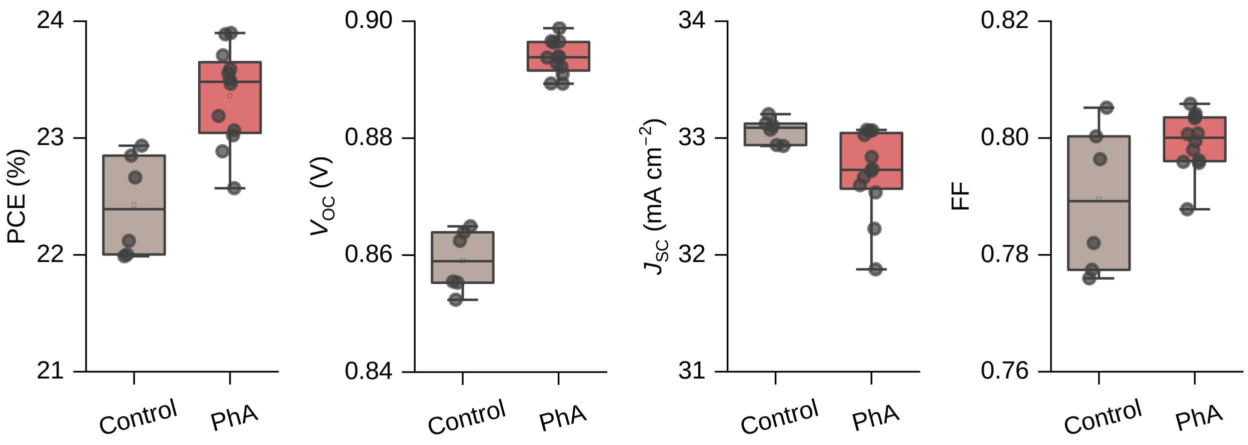


**Supplementary Fig. S33:** Statistical distribution of the PV parameters from the batch obtained the best-performing single-junction control (three cells) and PhA (six cells) Sn–Pb PSCs. Data are mean ± s.e.m.

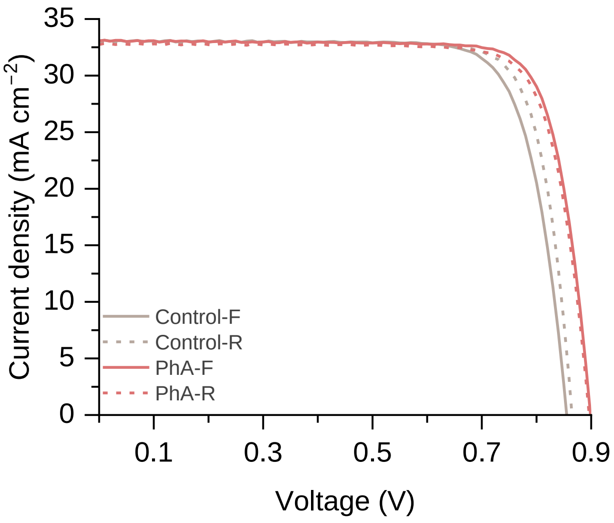


| Samples | $J_{SC}$ (mA cm$^{-2}$) | $V_{OC}$ (V) | FF | PCE (%) |
|---|---|---|---|---|
| Control-F | 33.07 | 0.86 | 0.78 | 22.12 |
| Control-R | 32.93 | 0.86 | 0.81 | 22.93 |
| PhA-F | 33.07 | 0.90 | 0.80 | 23.90 |
| PhA-R | 32.74 | 0.90 | 0.80 | 23.46 |

**Supplementary Fig. S34:** Representative *J–V* curves and cell parameters of the devices fabricated with the perovskite films deposited without (control) and with the addition of 3 mol% PhA. The unencapsulated devices were measured in the $N_2$-filled glovebox under the illumination of simulated AM1.5G.

**TPC results**

Despite the crystallinity being improved, the PhA-modified cells show statistically smaller $J_{SC}$ compared to the control cells, this could originate from the slightly reduced mobility of the resultant films, which should be attributed to the dielectric nature of the additive applied.

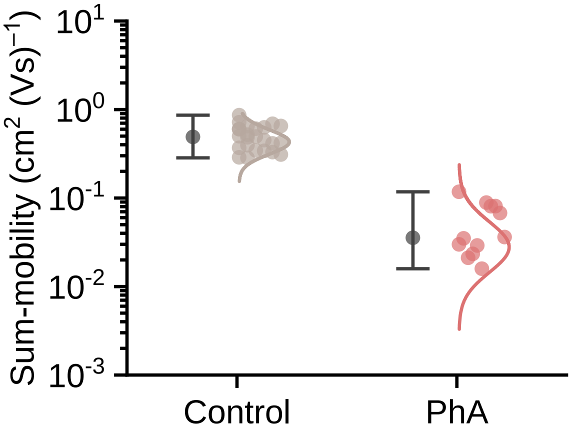


**Supplementary Fig. S35:** Long-range mobility of perovskite films estimated by transient photoconductivity measurements. The sum of mobilities ($\sum\mu$) is shown. Data are mean ± s.e.m.

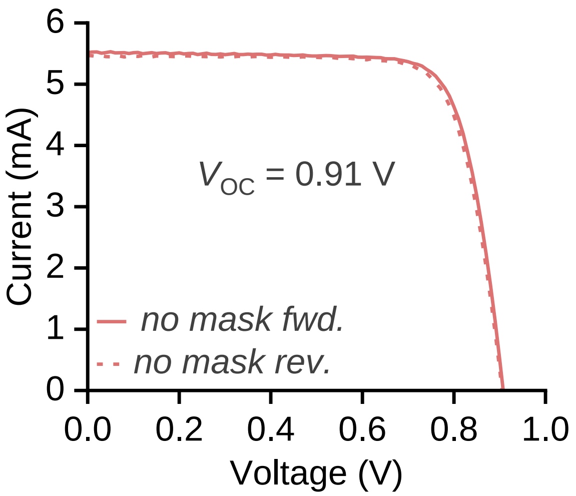


**Supplementary Fig. S36:** *I*–*V* curves for the champion Sn–Pb perovskite solar cell device measured with no optical mask, the estimated active area is around 0.16 $cm^2$.

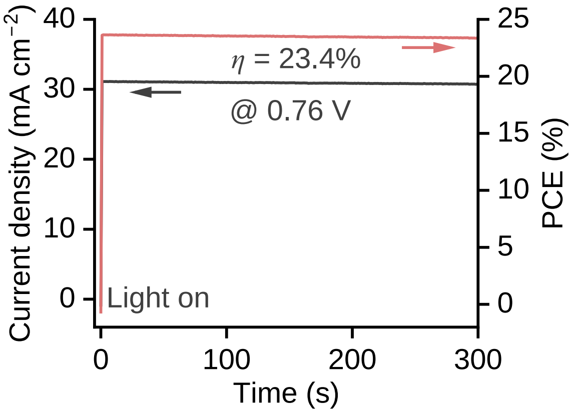


**Supplementary Fig. S37:** Steady-state power output of the PhA-modified Sn–Pb PSCs, measured by tracking the output current density at the fixed bias of 0.76 V under simulated AM1.5 100 mW cm$^{-2}$ illumination.

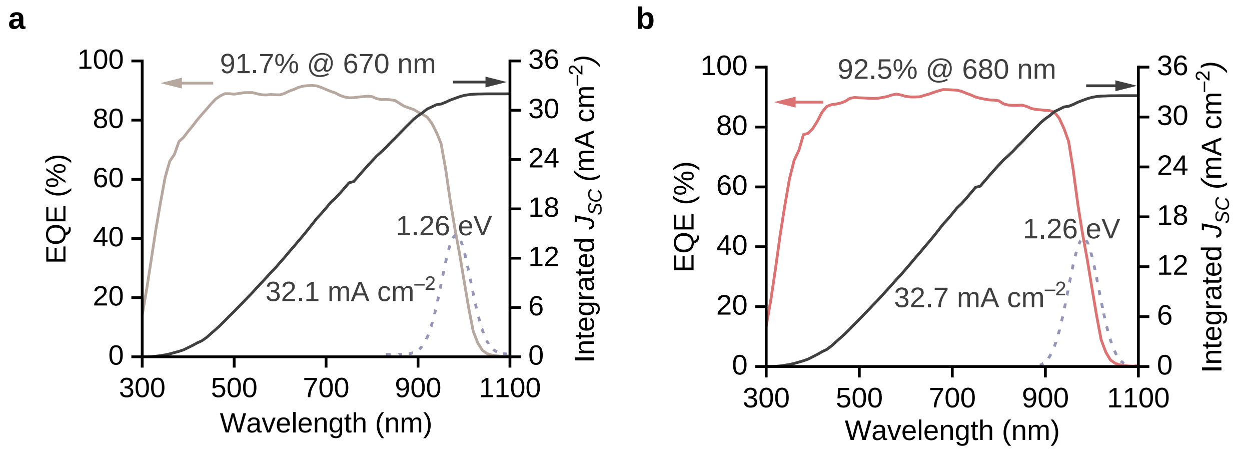


**Supplementary Fig. S38:** EQE spectrum and integrated $J_{SC}$ of representative **a,** control and **b,** PhA Sn–Pb PSC devices. –(dEQE/dλ) (purple dashed line) is shown to estimate the PV bandgap of the perovskite absorber.

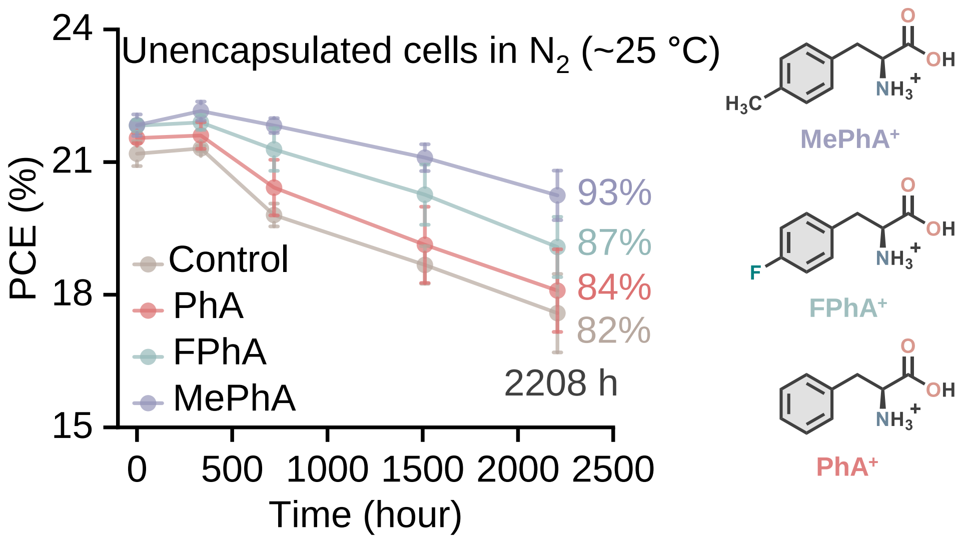


**Supplementary Fig. S39:** Shelf-stability of the unencapsulated cells with the architecture of FTO/PEDOT:PSS/perovskite/$C_{60}$ (20 nm)/BCP (8 nm)/Ag (100 nm) stored in a $N_2$-filled glovebox in the dark at room temperature (ISOS D-1)[5] for over three months (>2200 h), i.e., from Nov. 2022 until Feb. 2023. The cation structures of the chloride-based amino acid salt additives are shown on the right side of the plots. The error bar shows the average deviation of PCE values obtained from six devices for each condition. The devices were fabricated in the same batch and the amount of additives applied was 3 mol%. The 4FPhA and 4MePhA cells show slightly better efficiency than the PhA devices, and 4FPhA cells show statistically the best "shelf-stability" among the examined cases. As learned from our SEM experiments, we observe no distinct difference in the morphology of the perovskite films modified by PhA, 4FPhA, and 4MePhA. DFT calculations, however, suggest a slightly better defect passivation ability of 4FPhA and 4MePhA, compared to that of PhA, this could be one of the reasons responsible for small performance differences of the resultant modified devices. Data are mean ± s.e.m.

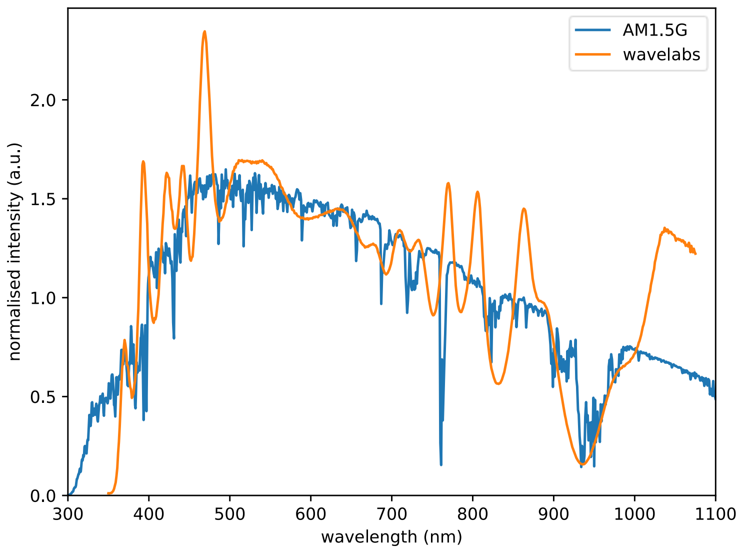


**Supplementary Fig. S40:** Spectra of the Wavelab SINUS-220 simulator, measured on the 10th of July 2024 (orange line), and AM1.5G, obtained from the NREL website (blue line).

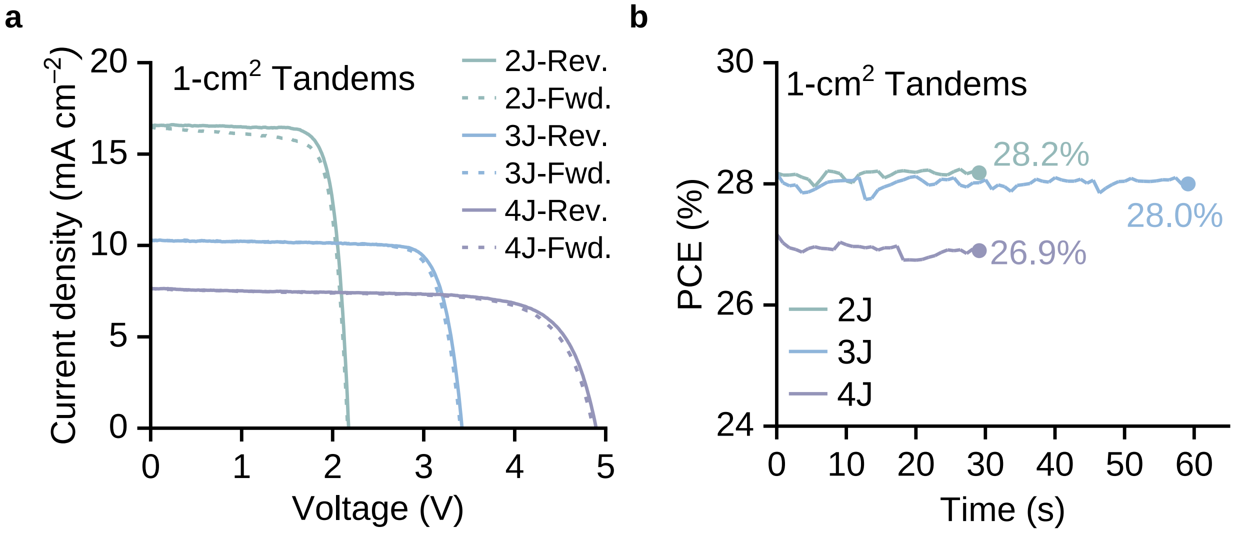


**Supplementary Fig. S41: a,** *J–V* curves and **b,** steady-state MPP tracking of the double-, triple-, and quadruple-junction tandems cells with the active area of 1 $cm^2$. In general, the *J–V* hysteresis observed may originate from the high density of the mobile ions in the perovskite materials originating from their ionic nature and potential charge accumulation and redistribution at the interfaces of the devices under forward and reverse scans. The level of hysteresis is also largely influenced by the scan rate of the *J–V* measurements, thus a steady-state measure on PCE is highlighted.

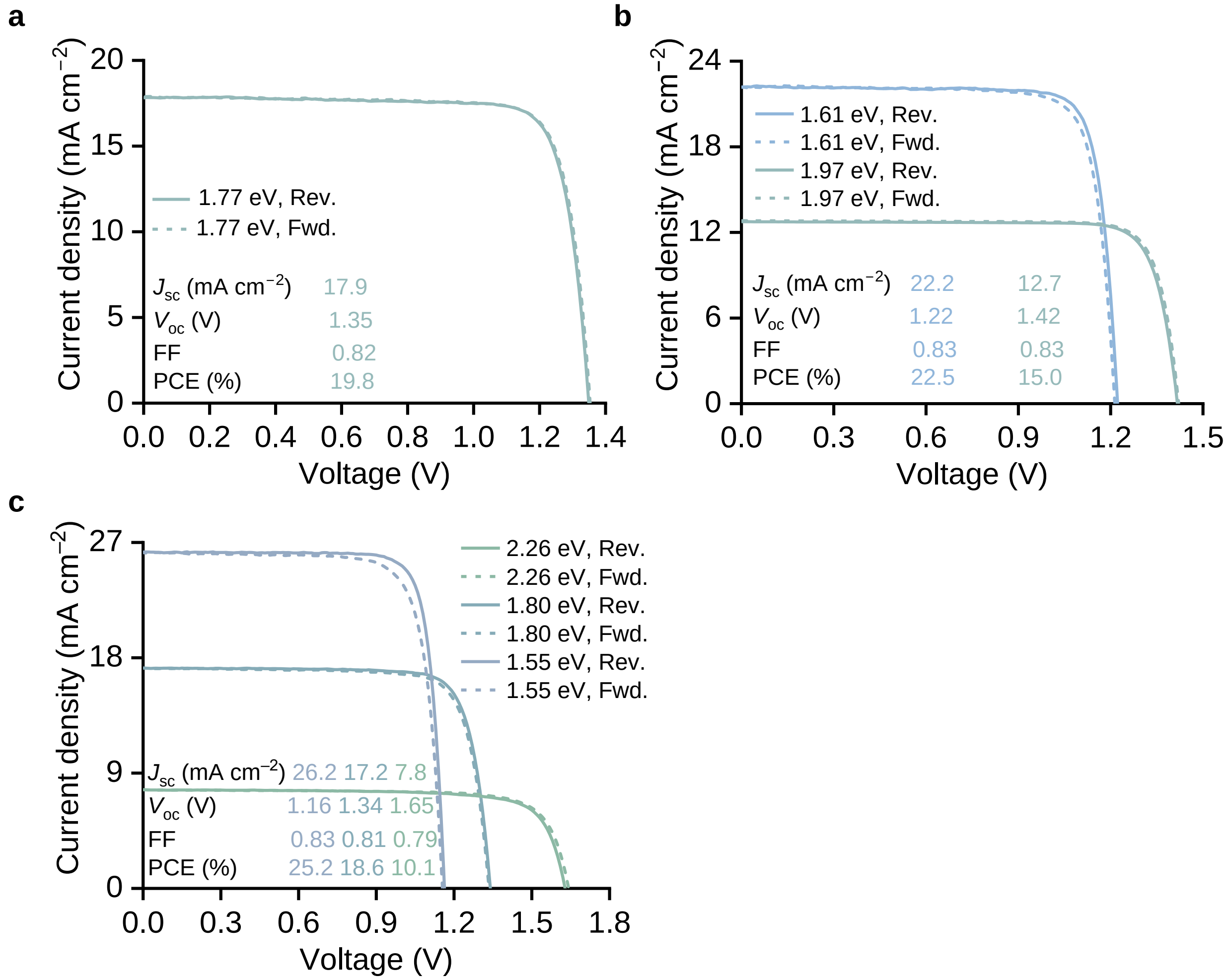


**Supplementary Fig. S42:** Representative *J–V* curves of single-junction neat Pb PSCs applied to the **a,** double-, **b,** triple-, and **c,** quadruple-junction cells. The PV parameters of the reverse scan are given. The bandgap values are determined by dEQE/dE given in **Supplementary Fig. S43**.

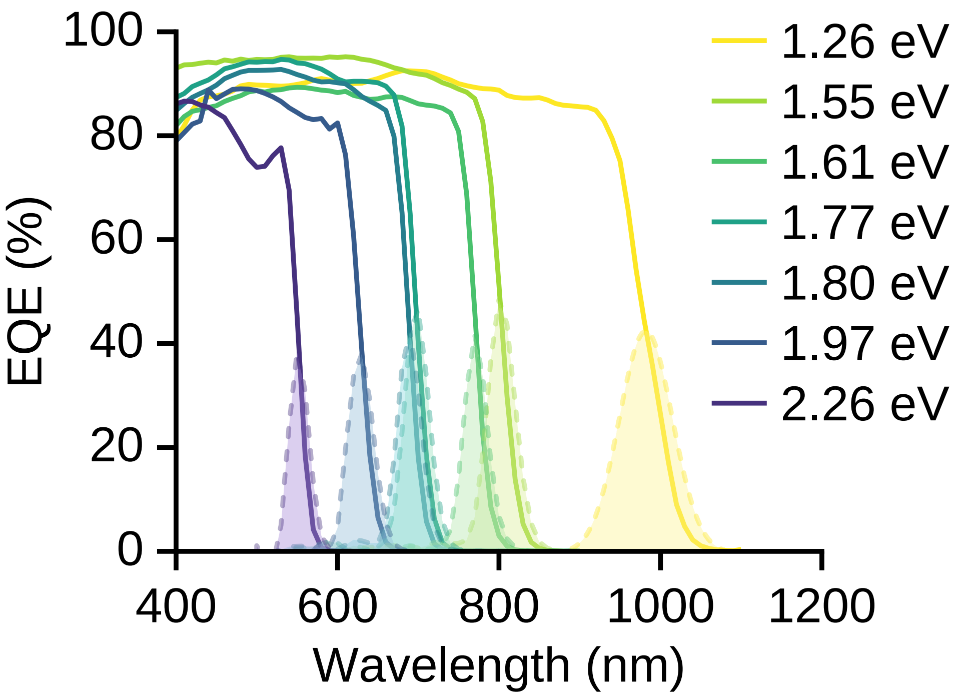


**Supplementary Fig. S43:** EQE spectra of 1.26 (32.7 mA $cm^{-2}$), 1.55 (25.3 mA $cm^{-2}$), 1.61 (21.8 mA $cm^{-2}$), 1.77 (18.3 mA $cm^{-2}$), 1.80 (17.3 mA $cm^{-2}$), 1.97 (12.9 mA $cm^{-2}$), and 2.26 eV (7.7 mA $cm^{-2}$) subcells applied for the fabrication of the tandem devices. The integrated current densities for the PSCs with individual bandgaps are given in the bracket after the bandgap values. The –(dEQE/dλ) was calculated to estimate the bandgap of the PSCs, shown with dotted lines. The absorber bandgap stacked in double- triple-, and quadruple-junction tandems is 1.77/1.26 eV, 1.97/1.61/1.26 eV, and 2.26/1.80/1.55/1.26 eV, respectively.

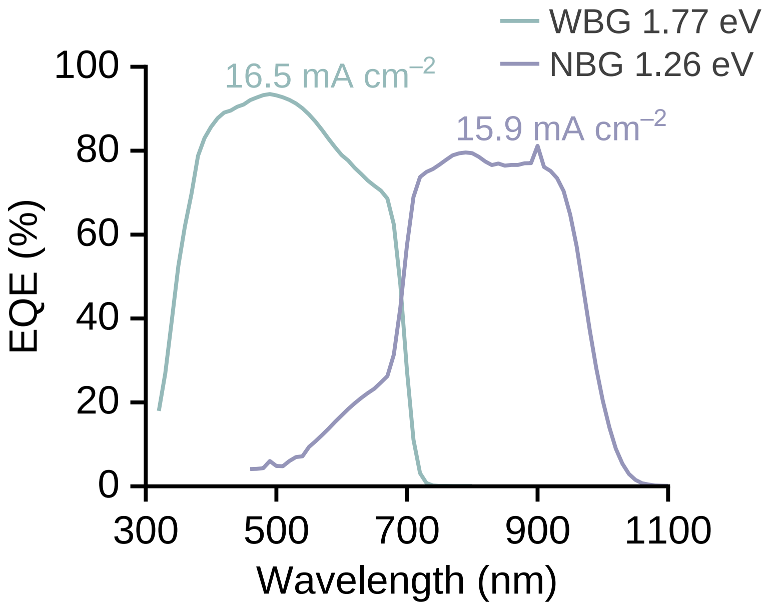


**Supplementary Fig. S44:** EQE spectra of 1.77 eV (16.5 mA $cm^{-2}$) and 1.26 (15.9 mA $cm^{-2}$) eV subcells in a double-junction tandem device. The estimated mismatch factor is 0.994 based on taking the junction with the lowest $J_{SC}$ in the stack for either our measured Wavelab solar simulator spectrum or standard AM1.5G light. Accurately measuring EQE responses of multijunction cells is generally challenging, due to the requirement to both optically and electrically bias the sub-cells. Determining the current limiting subjunction(s) in the multijunction cells reliably requires the ability to independently vary the irradiance of the different zones from the simulated sunlight and measure the intensity-dependent short-circuit current density from the multijunction cells[6].

## I-V CURVE

IEC 60904-3:2019 1.003 $cm^2$ (designated area)

WHSS

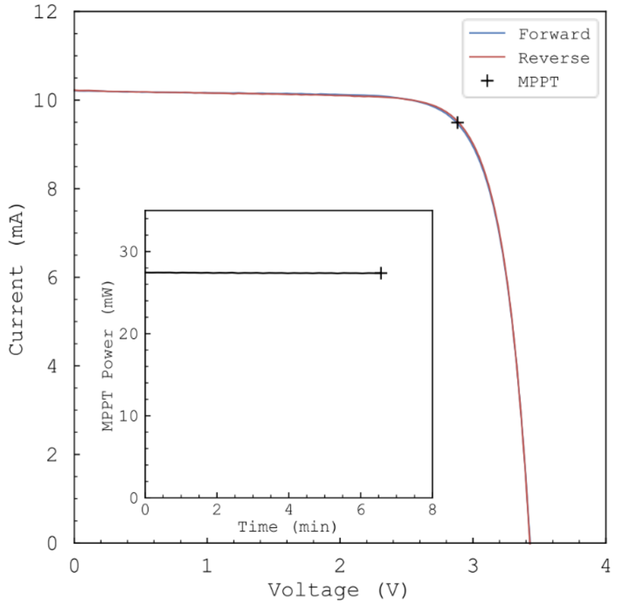


| | |
|---|---|
| Date | : 5 Jun 2024 |
| Sample No. | : 00DDEE(8-3) |
| Repeat Times | : 1 |

| Scan Mode | | Forward | Reverse | MPPT+ | |
|---|---|---|---|---|---|
| $I_{sc}$ | (mA) | 10.21 | 10.22 | - | |
| $V_{oc}$ | (V) | 3.430 | 3.428 | - | |
| $P_{max}$ | (mW) | 27.31 | 27.46 | **27.37** | |
| $I_{pmax}$ | (mA) | 9.48 | 9.50 | **9.49** | |
| $V_{pmax}$ | (V) | 2.882 | 2.892 | **2.884** | |
| FF | (%) | 78.0 | 78.4 | - | |
| Eff(da) | (%) | 27.22 | 27.37 | **27.28** | |
| DTemp. | (°C) | 25.0 | 25.0 | **25.0** | |
| MTemp. | (°C) | 25.1 | 25.1 | **25.1** | |
| DIrr. | ($mW/cm^2$) | 100.0 | 100.0 | **100.0** | |
| MIrr. | ($mW/cm^2$) | 100.0 | 100.0 | **100.0** | (top) |
| MIrr. | ($mW/cm^2$) | 100.0 | 100.0 | **100.0** | (mid) |
| MIrr. | ($mW/cm^2$) | 100.3 | 100.3 | **100.3** | (bot) |

| Ref. Device No. | Cal. Value (mA) | |
|---|---|---|
| CSI30+CM500S | 33.37 | (top) |
| CSI03+KG4-3t+A71 | 16.61 | (mid) |
| CSI30+RG9 | 39.99 | (bot) |

+: After MPPT 6.6 min

Total Illumination Time : 91 min

AIST

**Supplementary Fig. S45:** Certified results from the National Institute of Advanced Industrial Science and Technology (AIST, Japan) of a representative triple-junction cell with an active area of about 1 $cm^2$.

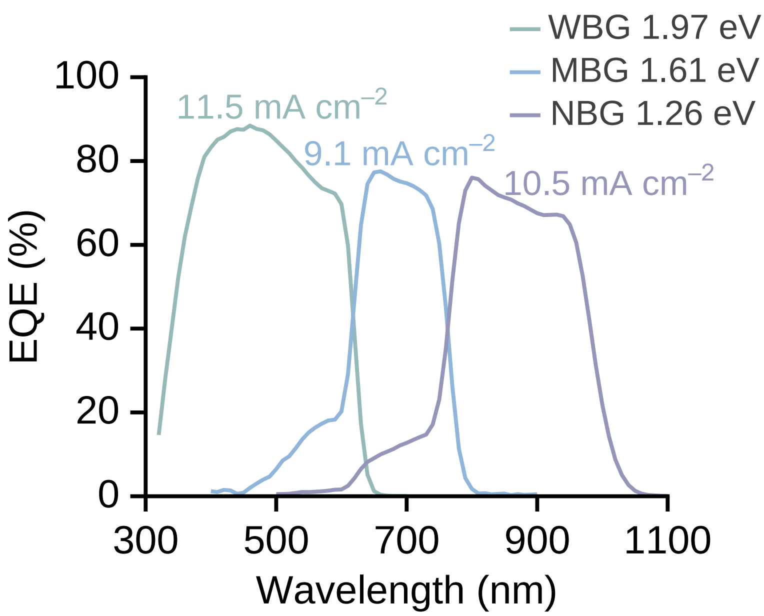


**Supplementary Fig. S46:** EQE spectra of 1.97 (11.5 mA cm$^{-2}$), 1.61 (9.1 mA cm$^{-2}$), and 1.26 eV (10.5 mA cm$^{-2}$) subcells in a triple-junction tandem device. The estimated mismatch factor is 0.997 based on taking the junction with the lowest $J_{SC}$ in the stack for either our measured Wavelab solar simulator spectrum or standard AM1.5G light.

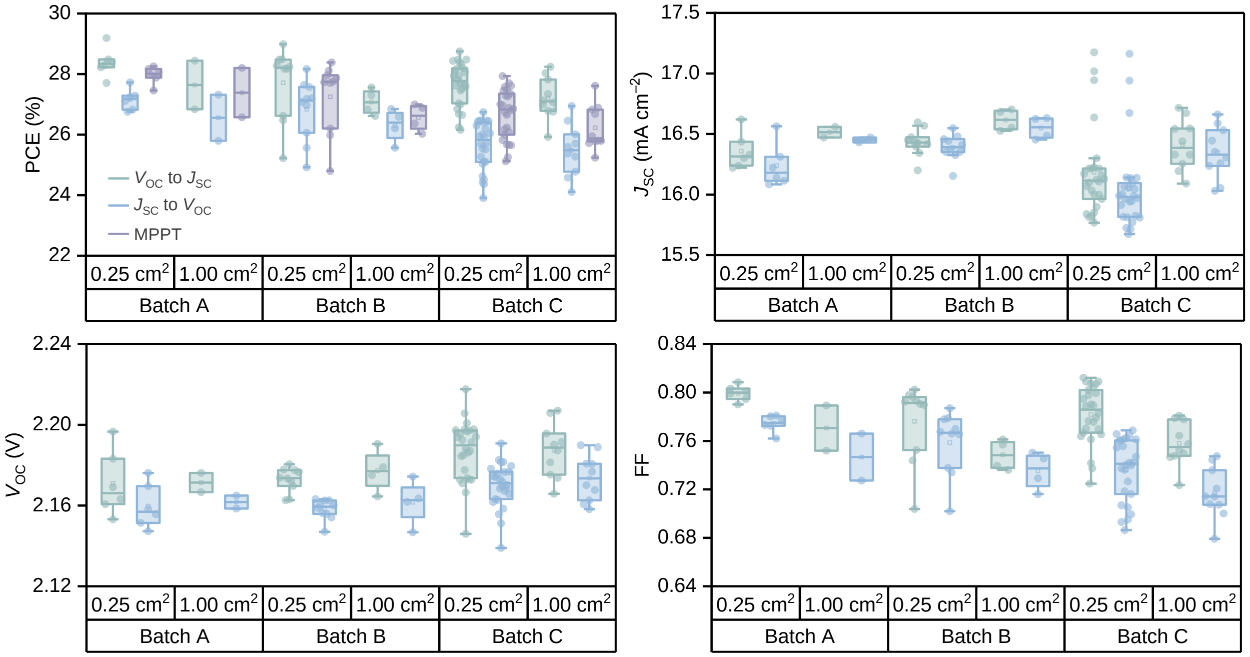


**Supplementary Fig. S47:** Statistical distributions of the PV device parameters for the double-junction all-perovskite tandem cells, fabricated in three different representative batches. The best PCE values obtained for the 0.25 and 1 cm$^2$ cells are 29.2 (steady-state 28.3%) and 28.4% (steady-state 28.2%), respectively. The best $V_{OC}$ values obtained for the 0.25 and 1 cm$^2$ cells are 2.218 V and 2.207 V, respectively. Data were collected from 58 cells. Data are mean ± s.e.m.

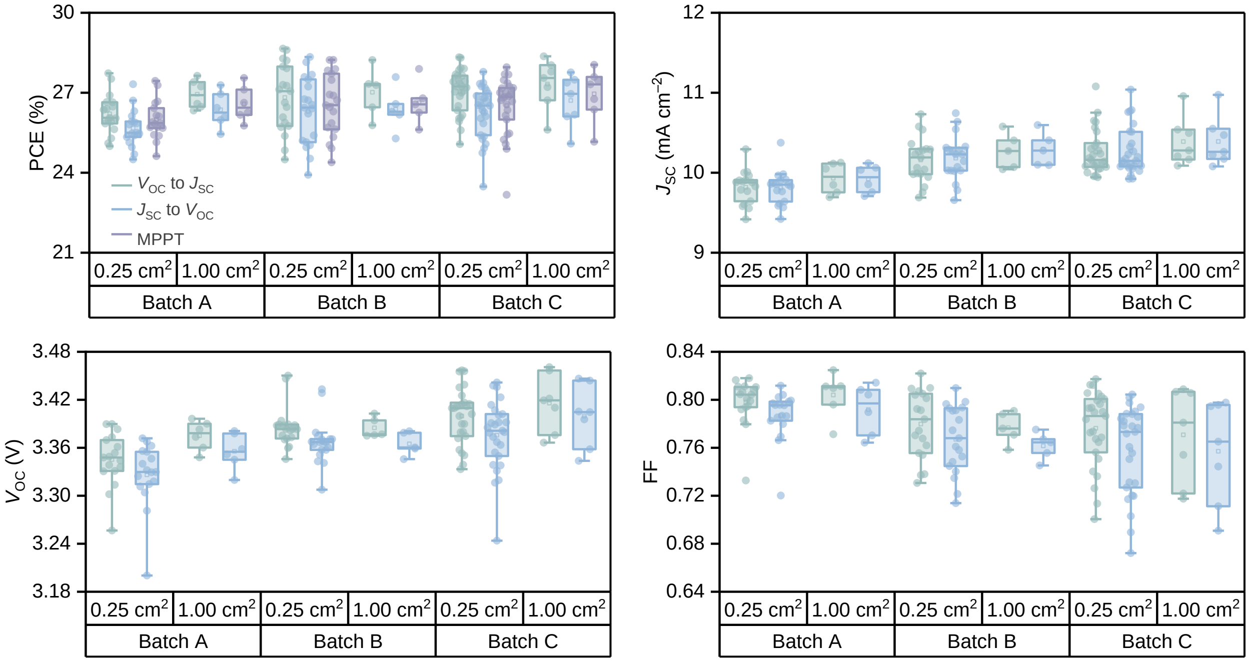


**Supplementary Fig. S48:** Statistical distributions of the PV device parameters for the triple-junction all-perovskite tandem cells, fabricated in three different representative batches. The best PCE values obtained for the 0.25 and 1 cm$^2$ cells are 28.7 (steady-state 28.2%) and 28.4% (steady-state 28.0%), respectively. The best $V_{OC}$ values obtained for the 0.25 and 1 cm$^2$ cells are 3.456 and 3.461 V, respectively. Data were collected from 81 cells. Data are mean ± s.e.m.

Test and Calibration Center of New Energy Device and Module,
Shanghai Institute of Microsystem and Information Technology,
Chinese Academy of Sciences (SIMIT)

MA 230017163974 · ilac-MRA · CNAS · 中国认可 国际互认 检测 TESTING CNAS L8490

## Measurement Report

**Report No. 24TR042903**

| | |
|---|---|
| **Client Name** | University of Oxford & Huazhong University of Science and Technology |
| **Client Address** | Parks Road, Oxford, OX1 3PU |
| **Sample** | Perovskite/Perovskite Tandem Solar Cell |
| **Manufacturer** | University of Oxford & Huazhong University of Science and Technology |
| **Measurement Date** | 29th April, 2024 |

| | | |
|---|---|---|
| **Performed by:** | Qiang Shi *Qiang Shi* | **Date:** 29/04/2024 |
| **Reviewed by:** | Wenjie Zhao *Wenjie Zhao* | **Date:** 29/04/2024 |
| **Approved by:** | Yucheng Liu *Yucheng Liu* | **Date:** 29/04/2024 |

**Address:** No.235 Chengbei Road, Jiading, Shanghai **Post Code:**201800
**E-mail:** solarcell@mail.sim.ac.cn **Tel:** +86-021-69976905

The measurement report without signature and seal are not valid.
This report shall not be reproduced, except in full, without the approval of SIMIT.

1 / 4

Report No. 24TR042903

| **Sample Information** | |
|---|---|
| Sample Type | Perovskite/Perovskite Tandem Solar Cell |
| Serial No. | 18# |
| Lab Internal No. | 24042901-3# |
| Measurement Item | I-V characteristic |
| Measurement Environment | 24.6±2.0℃,42.5±5.0%R.H |

| **Measurement of I-V characteristic** | |
|---|---|
| **Reference cell** | PVM 1121 |
| **Reference cell Type** | mono-Si, WPVS, calibrated by NREL (Certificate No. ISO 2098) |
| **Calibration Value/Date of Calibration for Reference cell** | 143.95mA/ Feb. 2024 |
| **Measurement Conditions** | Standard Test Condition (STC):<br>Spectral Distribution: AM1.5 according to IEC 60904-3 Ed.3,<br>Irradiance: 1000±50W/m², Temperature: 25±2℃ |
| **Measurement Equipment/ Date of Calibration** | AAA Steady State Solar Simulator (YSS-T155-2M) / July.2023<br>IV test system (ADCMT 6246) / June. 2023<br>SR Measurement system (CEP-25ML-CAS) / April.2023<br>Measuring Microscope (MF-B2017C) / July.2023 |
| **Measurement Method** | I-V measurement:<br>Logarithmic sweep in both directions (Voc to Isc and Isc to Voc) during one flash based on IEC 60904-1:2020.<br>Spectral Mismatch factor was calculated according to IEC 60904-7 and I-V correction according to IEC 60891.<br>MPP-Tracking:<br>Tracking for 300 seconds by P&O method, the reported Pmax represents the average value of the total data. |
| **Measurement Uncertainty** | Area: 1.0%(k=2); Isc: 2.0%(k=2); Voc: 1.0%(k=2);<br>Pmax: 2.7%(k=2); Eff:2.8%(k-2) |

2 / 4

Report No. 24TR042903

====Measurement Results ====

| | Forward Scan (Isc to Voc) | Reverse Scan (Voc to Isc) | MPP-Tracking |
|---|---|---|---|
| Area | | 4.98 mm² | |
| Isc | 0.815 mA | 0.808 mA | / |
| Voc | 2.182 V | 2.184 V | / |
| Pmax | 1.470 mW | 1.482 mW | 1.457 mW |
| Ipm | 0.769 mA | 0.774 mA | 0.775 mA |
| Vpm | 1.912 V | 1.914 V | 1.880 V |
| FF | 82.64 % | 83.95 % | / |
| Eff(aa) | 29.51 % | 29.76 % | 29.26 % |

- Spectral Mismatch Factor: $SMM_{top}$=0.9989, $SMM_{bot}$=0.9954.
- Designated illumination area defined by a thin metal mask was measured by the measuring microscope.
- The mask is smaller than glass substrate. Responses from outside of the assigned area were not considered.
- Test results listed in this measurement report refer exclusively to the mentioned measured sample.
- The results apply only at the time of the test, and do not imply future performance.

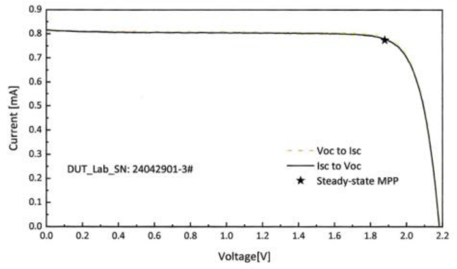


**Fig.1** I-V curves of the measured sample

3 / 4

Report No. 24TR042903

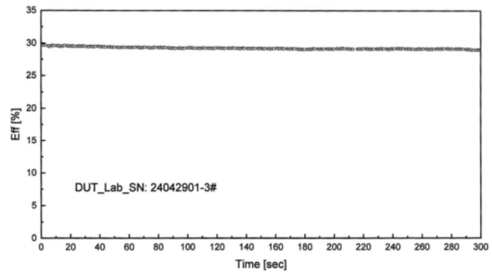


**Fig.2** Steady-state maximum power output of the measured sample

-------End of Report-------

4 / 4

**Supplementary Fig. S49:** Certified performance of double-junction cells fabricated with PhA-modified Sn–Pb subcells, the active device area is 0.0498 cm².

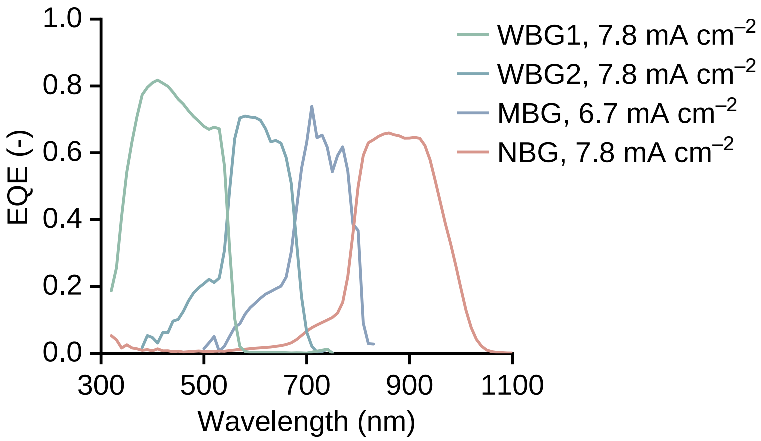


**Supplementary Fig. S50:** EQE spectra of WBG1 (7.8 mA $cm^{-2}$), WBG2 (7.8 mA $cm^{-2}$), MBG (6.7 mA $cm^{-2}$), and NBG (7.8 mA $cm^{-2}$) eV sub-cells in a representative quadruple-junction device.

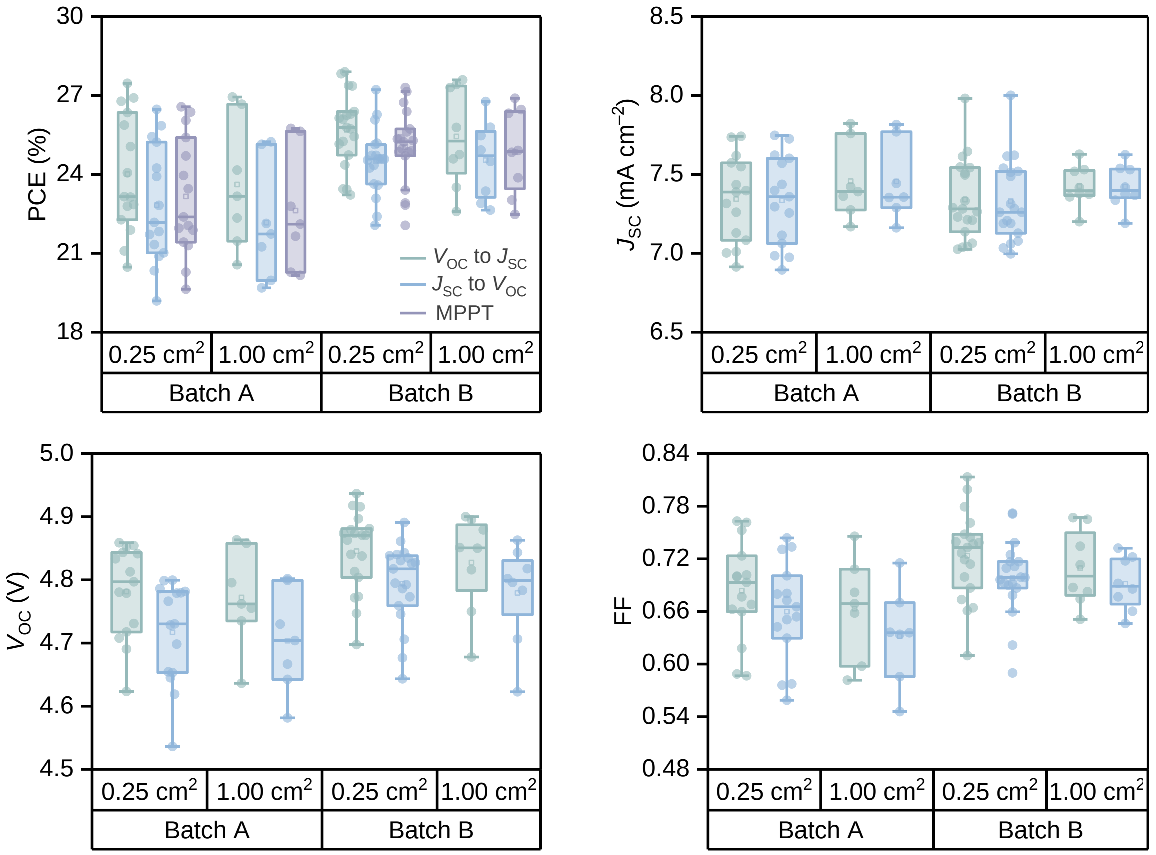


**Supplementary Fig. S51:** Statistical distributions of the PV device parameters for the quadruple-junction all-perovskite tandem cells, fabricated in two different representative batches. The best PCE values obtained for the 0.25 and 1 cm$^2$ cells are 27.9 (steady-state 27.4%) and 27.4% (steady-state 26.9%), respectively. The best $V_{OC}$ values obtained for the 0.25 and 1 cm$^2$ cells are 4.94 and 4.90 V, respectively. Data were collected from 49 cells.

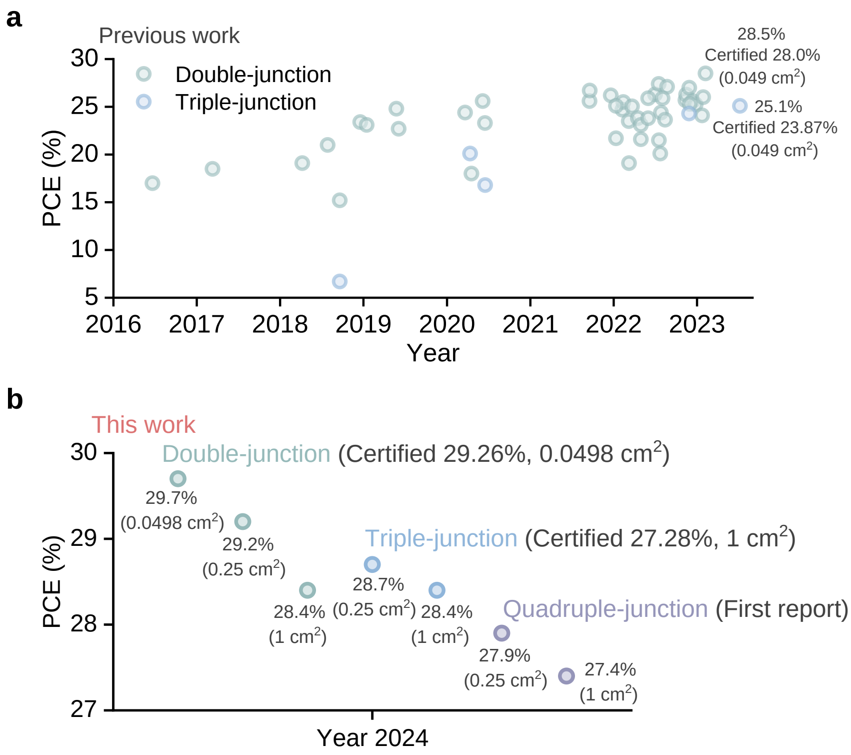


**Supplementary Fig. S52:** Efficiency progress of all-perovskite tandem solar cells with the data collected from **a,** the previous report[4] (the record PCE values are given[7,8].) and **b,** our current work. The active area of the devices is provided in the bracket.

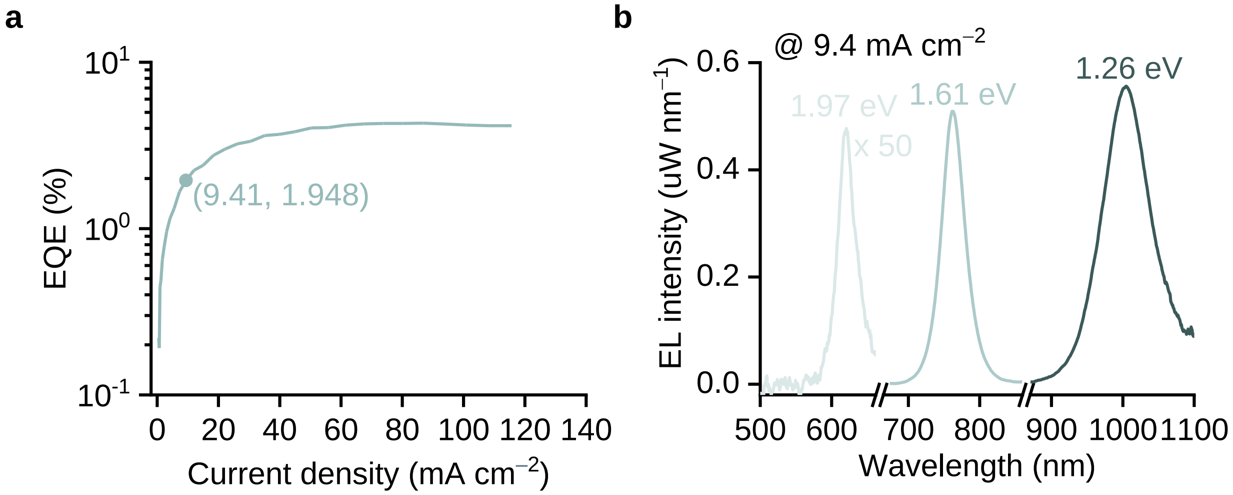


**Supplementary Fig. S53:** $EQE_{EL}$ characterizations. **a,** $EQE_{EL}$ values of a representative triple-junction cell measured as a function of applied current densities. **b,** EL spectra of the triple-junction cell measured at the current density of about 9.4 mA cm$^{-2}$, a value close to the device current density obtained under simulated AM1.5G illumination. The EL intensities of the MBG and NBG subcells could be slightly overestimated considering the contribution of the photons emitted from the subcell(s) stacked in front.

**Supplementary Table S3:** The measured $EQE_{EL}$ values of the triple-junction cells and values interpreted from the measurement or theoretical limit. The triple-junction device applied for the $EQE_{EL}$ measurements shows a PCE of 26.6% ($V_{OC}$: 3.43 V, $J_{SC}$: 9.8 mA cm$^{-2}$, FF: 0.79).

| Samples | $EQE_{EL}$ (%) @ 9.4 mA cm$^{-2}$ | $V_{OC}$ estimated (V) | $V_{OC}$ loss (mV) | 95% of the $V_{OC}$ limit[9] (V) |
|---|---|---|---|---|
| Triple-junction cell | 1.95 | 3.46 | 498.4 | 3.76 |
| 1.97-eV subcell | 0.01 | 1.40 | 251.7 | 1.57 |
| 1.61-eV subcell | 0.48 | 1.18 | 137.7 | 1.25 |
| 1.26-eV subcell | 1.46 | 0.88 | 109.0 | 0.94 |

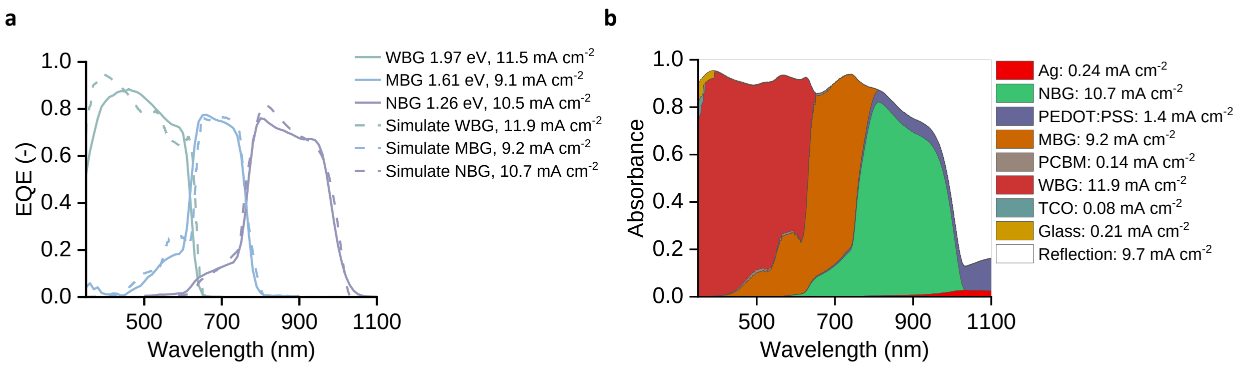


**Supplementary Fig. S54:** Optical modelling to fit the EQE spectra. **a,** Measured and optically simulated EQE spectra of the triple-junction cells. **b,** Simulated absorbance of triple-junction cells using the thickness of 250, 500, and 700 nm for the WBG, MBG, and NBG perovskite subabsorbers, respectively.

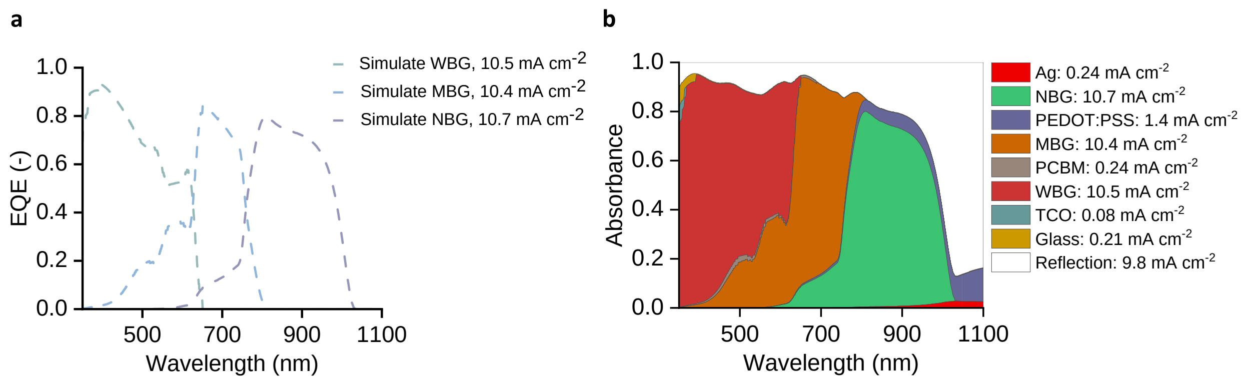


**Supplementary Fig. S55:** Optical modelling to optimise the thickness of the wide-bandgap subabsorber. **a,** Simulated EQE spectra of the triple-junction cells. **b,** Simulated absorbance of triple-junction cells using the thickness of 177, 500, and 700 nm for the WBG, MBG, and NBG perovskite subabsorbers, respectively.

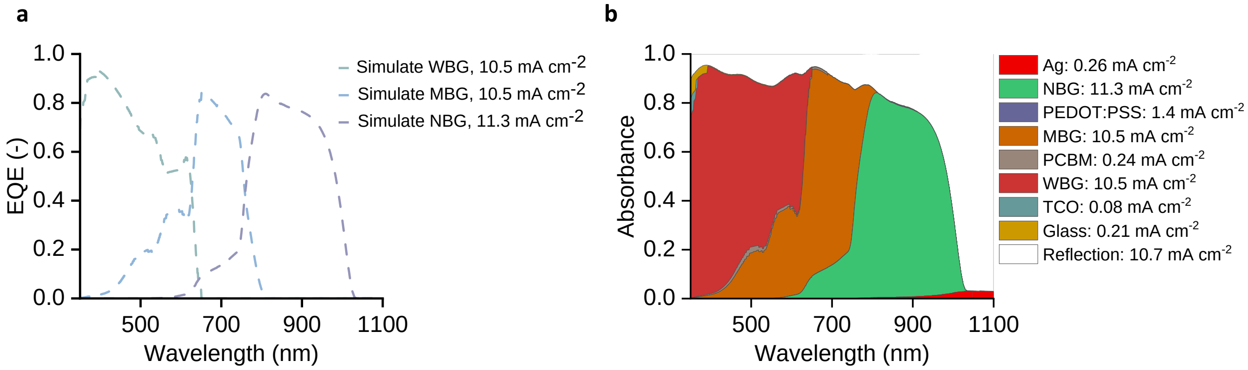


**Supplementary Fig. S56:** Optical modelling by removing PEDOT:PSS. **a,** Simulated EQE spectra of the triple-junction cells. **b,** Simulated absorbance of triple-junction cells using the thickness of 177, 500, and 700 nm for the WBG, MBG, and NBG perovskite subabsorbers, respectively. The improved optical response of the NBG subcell benefits from the removal of the PEDOT:PSS layer in the device stack.

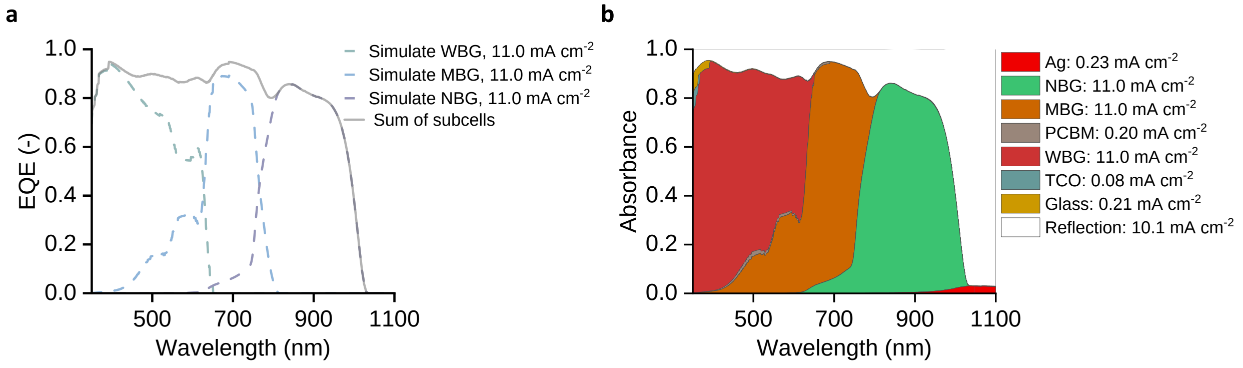


**Supplementary Fig. S57:** Optical modelling to fine-tune all absorber layer thicknesses. **a,** Simulated EQE spectra of the triple-junction cells. **b,** Simulated absorbance of PEDOT:PSS-free triple-junction cells using the thickness of 203, 727, and 966 nm for the WBG, MBG, and NBG perovskite subabsorbers, respectively. The sum of the optical response of the optimal cell suggests EQE values of over 90% on average for the WBG and MBG subcells, leaving room for improvement of the NBG subcell with the current maximum response of no more than 85% EQE value.

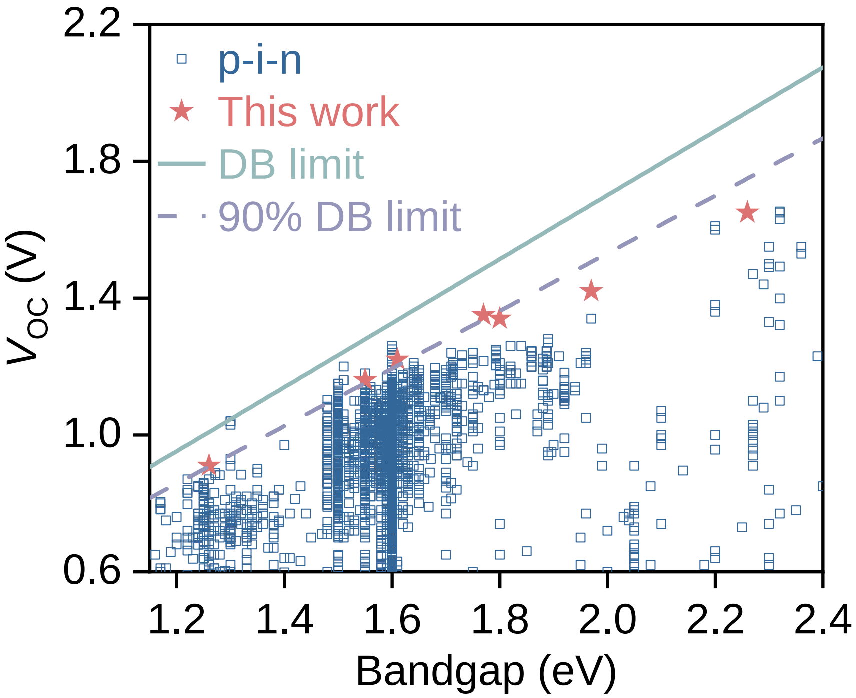


**Supplementary Fig. S58:** Bandgap-dependent $V_{OC}$ for p-i-n single-junction PSCs. The cell data were acquired from an open-access perovskite solar cell database[10]. In the plot, the DB (detailed balance) limit (solid line), 90% of the DB limit (dashed line), the highest $V_{OC}$ of this work (solid star) and open-source data[10] (open brackets) are shown. Interestingly, the "$V_{OC}$-loss" for the 1.97 eV mixed halide perovskite and the 2.26 eV neat bromide-based perovskite is approximately the same. This suggests that halide segregation may not be the primary factor limiting the $V_{OC}$ of the widest bandgap PSCs, but rather the high defect density in Br-rich perovskites and increased surface recombination losses at the perovskite/charge extraction layer interfaces, in part, due to increased mismatch of the energy levels. Identifying and overcoming these losses is important to unlock the potential of the multijunction cells further.

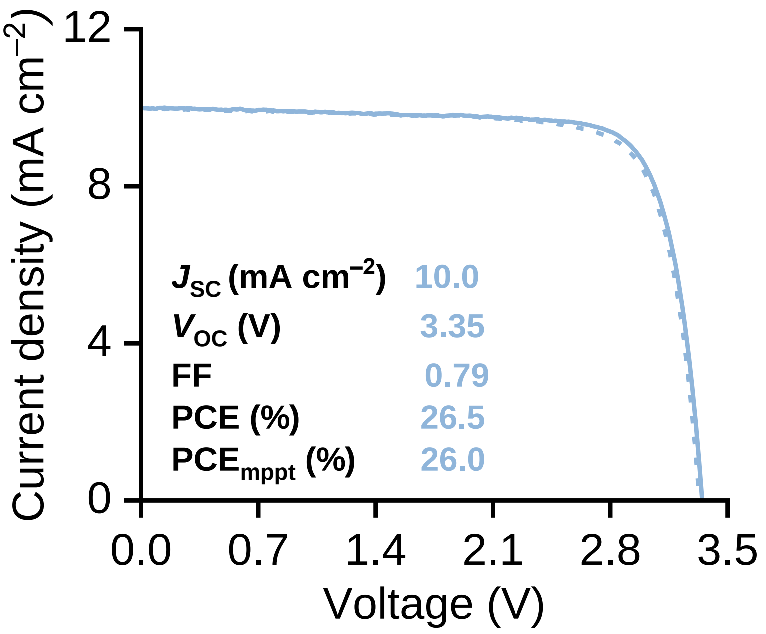


**Supplementary Fig. S59:** *J*–*V* curves of the triple-junction cells subjected to the MPP tracking test. The result is given in **Fig. 4g**. The top contact of the device is Cr (2.5 nm)/Au (20 nm)/Ag (100 nm)

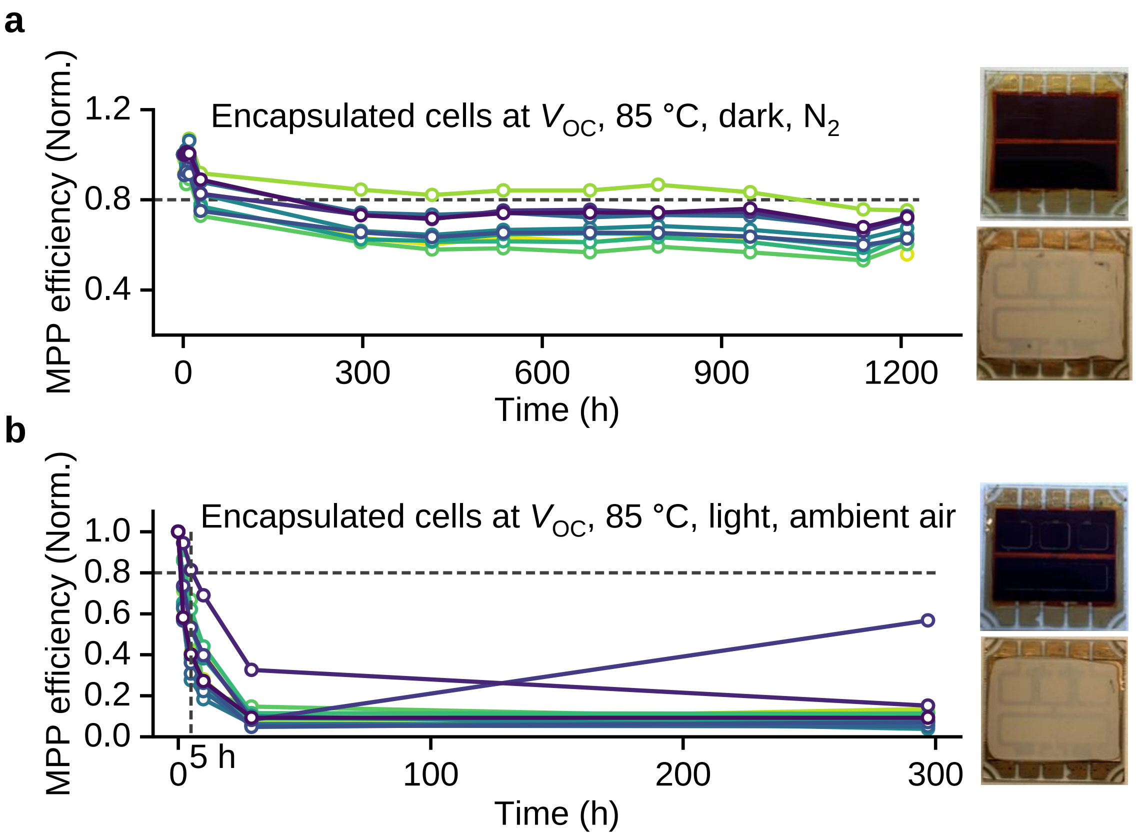


**Supplementary Fig. S60:** Thermal stability of encapsulated triple-junction cells with the top contact of Cr (2.5 nm)/Au (90 nm). All devices were aged under 85 °C at open-circuit voltage under **a,** dark ($N_2$-filled glovebox, $O_2$ < 10 ppm, $H_2O$ < 0.1 ppm) and **b,** simulated sunlight (~76 mW $cm^{-2}$ irradiances, ambient air, no UV filter was applied) conditions. The photographs of one of the aged cells for each condition are shown on the right side. All the device PV parameters decrease concurrently as the ageing time increases. Under elevated temperature and light ageing conditions, good wide bandgap single-junction PSCs last for hundreds to thousands of hours[11].

a

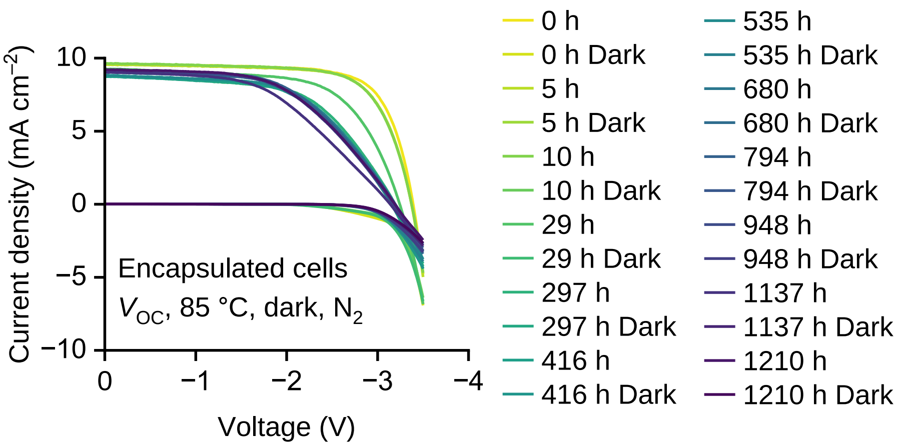


b

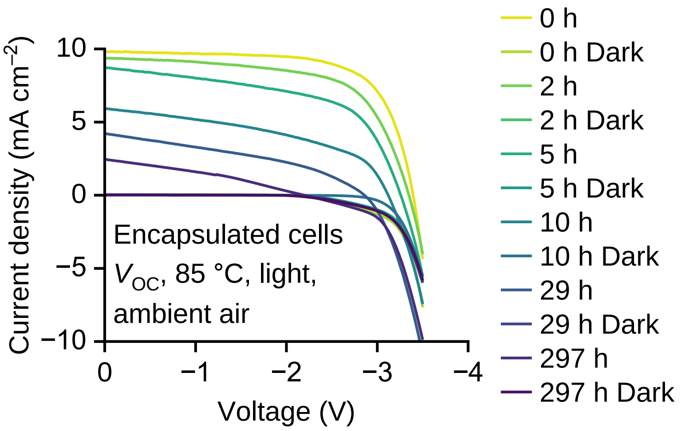


**Supplementary Fig. S61:** *J–V* curve evolution of the representative triple-junction devices subjected to the 85 °C **a,** dark and **b,** light ageing tests. Both the illuminated and dark *J–V* curves are shown. For clarity, only reverse *J–V* scans are shown. The top contact of the devices is Cr (2.5 nm)/Au (90 nm). As suggested by previous studies investigating Sn–Pb perovskites and all-perovskite tandem solar cells[12,13], there is an urgent need to replace the acidic PEDOT:PSS with unreactive/less reactive materials[4], for example, PTAA or hole-selective SAMs, for more stable Sn–Pb perovskites and their multijunction tandem cells. However, cells fabricated with PTAA and SAMs exhibit either lower efficiency or reduced reproducibility compared to PEDOT:PSS cells, suggesting further investigations for potential improvements. Apart from the origin related to the narrow bandgap Sn–Pb perovskite subjunction, we believe that the degradation of the multijunction cells is a result of a global contribution from all the loss of materials and reactions at the perovskite interfaces (as suggested by the photo images of the aged devices) and bulk, for example, the notorious light-induced halide segregation[14] in the Br-rich wide-bandgap (~2 eV) perovskite as we studied before[8]. To draw a comprehensive picture of the degradation routes of multijunction cells, we expect further extensive and inclusive studies from our group and the others in the community in the future.

## References

1 Hu, S. *et al.* Optimized carrier extraction at interfaces for 23.6% efficient tin–lead perovskite solar cells. *Energy Environ. Sci.* **15**, 2096–2107 (2022). https://doi.org/10.1039/d2ee00288d

2 Hu, S. *et al.* Synergistic surface modification of tin–lead perovskite solar cells. *Adv. Mater.* **35**, 2208320 (2023). https://doi.org/https://doi.org/10.1002/adma.202208320

3 Hu, S. *et al.* Mixed lead-tin perovskite films with >7 μs charge carrier lifetimes realized by maltol post-treatment. *Chem. Sci.* **12**, 13513–13519 (2021). https://doi.org/10.1039/d1sc04221a

4 Hu, S. *et al.* Narrow Bandgap Metal Halide Perovskites for All-Perovskite Tandem Photovoltaics. *Chem. Rev.* **124**, 4079–4123 (2024). https://doi.org/10.1021/acs.chemrev.3c00667

5 Khenkin, M. V. *et al.* Consensus statement for stability assessment and reporting for perovskite photovoltaics based on ISOS procedures. *Nat. Energy* **5**, 35–49 (2020). https://doi.org/10.1038/s41560-019-0529-5

6 Song, T., Mack, C., Williams, R., Friedman, D. J. & Kopidakis, N. How Should Researchers Measure Perovskite-Based Monolithic Multijunction Solar Cells' Performance? A Calibration Lab's Perspective. *Sol. RRL* **6**, 2200800 (2022). https://doi.org/https://doi.org/10.1002/solr.202200800

7 Lin, R. *et al.* All-perovskite tandem solar cells with 3D/3D bilayer perovskite heterojunction. *Nature* **620**, 994–1000 (2023). https://doi.org/10.1038/s41586-023-06278-z

8 Wang, J. *et al.* Halide homogenization for low energy loss in 2-eV-bandgap perovskites and increased efficiency in all-perovskite triple-junction solar cells. *Nat. Energy* **9**, 70–80 (2023). https://doi.org/10.1038/s41560-023-01406-5

9 Rühle, S. Tabulated values of the Shockley–Queisser limit for single junction solar cells. *Sol. Energy* **130**, 139–147 (2016). https://doi.org/https://doi.org/10.1016/j.solener.2016.02.015

10 Jacobsson, T. J. *et al.* An open-access database and analysis tool for perovskite solar cells based on the FAIR data principles. *Nat. Energy* **7**, 107–115 (2022). https://doi.org/10.1038/s41560-021-00941-3

11 Lin, Y.-H. *et al.* Bandgap-universal passivation enables stable perovskite solar cells with low photovoltage loss. *Science* **384**, 767–775 (2024). https://doi.org/10.1126/science.ado2302

12 Chen, B. *et al.* Bifacial all-perovskite tandem solar cells. *Sci. Adv.* **8**, eadd0377 (2022). https://doi.org/10.1126/sciadv.add0377

13 Rombach, F. *et al.* Disentangling Degradation Pathways of Narrow Bandgap Lead-Tin Perovskite Material and Photovoltaic Devices. *Research Square* (2024). https://doi.org/https://doi.org/10.21203/rs.3.rs-4502930/v1

14 Hoke, E. T. *et al.* Reversible photo-induced trap formation in mixed-halide hybrid perovskites for photovoltaics. *Chem. Sci.* **6**, 613–617 (2015). https://doi.org/10.1039/C4SC03141E
