# Supplementary material for: Steering perovskite precursor solutions for multijunction photovoltaics
Source: Nature. 2024 Dec 23;639(8053):93–101. doi: 10.1038/s41586-024-08546-y (PMC11882461; doi:10.1038/s41586-024-08546-y)
Supplement: Supplementary file 2 — Reporting Summary [file 41586_2024_8546_MOESM2_ESM.pdf]

nature portfolio

Corresponding author(s): Henry J. Snaith

Solar Cells Reporting Summary

Nature Portfolio wishes to improve the reproducibility of the work that we publish. This form is intended for publication with all accepted papers reporting the characterization of photovoltaic devices and provides structure for consistency and transparency in reporting. Some list items might not apply to an individual manuscript, but all fields must be completed for clarity.

For further information on Nature Research policies, including our data availability policy, see Authors & Referees.

Experimental design

Please check the following details are reported in the manuscript, and provide a brief description or explanation where applicable.

1. Dimensions

Area of the tested solar cells

- [x] Yes
- [ ] No

Aperture areas used in the study are 1.0 cm2 (Fig. S41, S45), 0.25 cm2 (Fig. 4c, 4f, S42, S59, S61), 0.0985 cm2 (Fig. 4c, S24, S26, S28, S31, S34,), and 0.0498 cm2 (Fig. S49)

Explain why this information is not reported/not relevant.

Method used to determine the device area

- [x] Yes
- [ ] No

Defined by the shadow mask.

Explain why this information is not reported/not relevant.

2. Current-voltage characterization

Current density-voltage (J-V) plots in both forward and backward direction

- [x] Yes
- [ ] No

Fig. 4c, S24, S26, S28, S31, S34, S36, S41, S42, S45, S49, S59

Voltage scan conditions

- [x] Yes
- [ ] No

The scan rate was approximately 0.1-0.3 V/s for both scan directions

Explain why this information is not reported/not relevant.

Test environment

- [x] Yes
- [ ] No

Solar cells were measured in ambient air with encapsulation.

Explain why this information is not reported/not relevant.

Protocol for preconditioning of the device before its characterization

- [ ] Yes
- [x] No

Provide a description of the protocol.

No preconditioning protocol was used

Stability of the J-V characteristic

- [x] Yes
- [ ] No

Stability measurements were performed by tracking the maximum power point (Fig. 4d, 4g, S41, S45, S49) or stabilized steady state (Fig. 4d, S37).

Explain why this information is not reported/not relevant.

3. Hysteresis or any other unusual behaviour

Description of the unusual behaviour observed during the characterization

- [ ] Yes
- [x] No

Provide a description of hysteresis or any other unusual behaviour observed during the characterization.

Negligible hysteresis

Related experimental data

- [x] Yes
- [ ] No

Fig. 4c, S24, S26, S28, S31, S34, S36, S41, S42, S45, S49, S59

Explain why this information is not reported/not relevant.

4. Efficiency

External quantum efficiency (EQE) or incident photons to current efficiency (IPCE)

- [x] Yes
- [ ] No

Fig. S30, S38, S43, S44, S46, S50

Explain why this information is not reported/not relevant.

A comparison between the integrated response under the standard reference spectrum and the response measure under the simulator

- [x] Yes
- [ ] No

The integrated short-circuit current from the standard AM1.5 G spectrum matches the short-circuit current from the J-V measurements within 5%.

Explain why this information is not reported/not relevant.

For tandem solar cells, the bias illumination and bias voltage used for each subcell

☒ Yes ☐ No

For double-junction cells, a 850-nm LED bias was used to measure the response from the 1.77 eV front sub-cell and a 455-nm LED bias was used to measure the response from the 1.26 eV back sub-cell. For triple-junction cells, 850- and 740-nm LED bias were used to measure the response from the 1.97 eV front sub-cell, 850- and 455-nm LED bias were used to measure the response from the 1.61 eV middle sub-cell, and 740- and 455-nm LED bias were used to measure the response from the 1.26 eV back sub-cell. For quadruple-junction dells, 585, 740 and 850 nm LED lights were used to measure the EQE response from the WBG1 (2.26 eV) subcell. 455, 740, and 850 nm LED lights were used to measure the EQE response from the WBG2 subcell. 455, 585, and 850 nm LED lights were used to measure the EQE response from the MBG subcell. 455, 585, and 740 nm lights were used to measure the EQE response from the NBG subcell. No bias voltage was used during the measurement.

Explain why this information is not reported/not relevant.

5. Calibration

Light source and reference cell or sensor used for the characterization

☒ Yes ☐ No

AM1.5G solar radiance was generated by a Wavelabs SINUS-220 solar simulator and calibrated with WPVS reference cell (monocrystalline silicon solar cell, provided and certified by Fraunhofer ISE) matched its 1-sun certified value.

Explain why this information is not reported/not relevant.

Confirmation that the reference cell was calibrated and certified

☒ Yes ☐ No

The solar simulator was calibrated with WPVS reference cell (monocrystalline silicon solar cell, provided and certified by Fraunhofer ISE) matched its 1-sun certified value.

Explain why this information is not reported/not relevant.

Calculation of spectral mismatch between the reference cell and the devices under test

☐ Yes ☒ No

Provide a value of the spectral mismatch and/or a description of how it has been taken into account in the measurements.

Estimated mismatch factor is less than 1 and was hence not applied.

6. Mask/aperture

Size of the mask/aperture used during testing

☒ Yes ☐ No

Mask size 0.0498, 0.0985, 0.25, and 1 cm2

Explain why this information is not reported/not relevant.

Variation of the measured short-circuit current density with the mask/aperture area

☒ Yes ☐ No

All short-circuit current densities were measured always with masks.

Explain why this information is not reported/not relevant.

7. Performance certification

Identity of the independent certification laboratory that confirmed the photovoltaic performance

☒ Yes ☒ No

National Institute of Advanced Industrial Science and Technology (AIST, Japan) and Shanghai Institute of Microsystem and Information Technology (SIMIT, China) calibrated the tandem cells.

Explain why this information is not reported/not relevant.

A copy of any certificate(s)

☒ Yes ☐ No

Certificate copies should be provided in the Supplementary information. Please state the supplementary item number.

Fig. S45, S49

8. Statistics

Number of solar cells tested

☒ Yes ☐ No

Over 200 devices were tested.

Explain why this information is not reported/not relevant.

Statistical analysis of the device performance

☒ Yes ☐ No

Fig. S25, S27, S29, S32, S33, S47, S48, S51. The number of cells used for each variation was presented under the figure captions.

Explain why this information is not reported/not relevant.

9. Long-term stability analysis

Type of analysis, bias conditions and environmental conditions

☒ Yes ☐ No

The long-term stability was performed by operating the encapsulated cell at the maximum power point under continuous simulated AM1.5G illumination at room temperature in ambient air (Fig. 4g), and by testing JV curves and statistic plots for the cells aged under 85 °C at open-circuit voltage under dark (Fig. S60, S61, N2-filled glovebox, O2 < 10 ppm, H2O < 0.1 ppm) and simulated sunlight (Fig. S60, S61, ~76 mW cm–2 irradiances, ambient air, no UV filter was applied) conditions.

Explain why this information is not reported/not relevant.
